# German guidelines for the diagnosis and treatment of squamous-cell carcinoma and adenocarcinoma of the esophagus—version 4.0

**DOI:** 10.1016/j.esmogo.2024.100112

**Published:** 2025-01-06

**Authors:** M.P. Ebert, W. Fischbach, S. Hollerbach, J. Höppner, D. Lorenz, M. Stahl, M. Stuschke, O. Pech, U. Vanhoefer, C. Bruns, C. Ell, M. Follmann, U. Goerling, L. Grenacher, J. Haardt, A.H. Hölscher, R. Hummel, W.T. Knoefel, J. Körber, R. Langer, P. Lenz, F. Lordick, S. Lorenzen, A.G. Meining, J. Menzel, H.-J. Meyer, N.H. Nicolay, M. Nothacker, U. Nöthlings, H. Schmidberger, M. Schmidt, T. Seufferlein, P. Thuss-Patience, J. Trojan, A. Weimann, L. Klug, P. Lynen, T. Zhan, Q. Xiao, R. Porschen

**Affiliations:** 1 II. Medizinische Klinik, Medizinische Fakultät Mannheim, Universitätsmedizin, Universität Heidelberg, Mannheim, Germany; 2 DKFZ-Hector Krebsinstitut an der Universitätsmedizin Mannheim, Mannheim, Germany; 3 Molecular Medicine Partnership Unit, European Molecular Biology Laboratory (EMBL), Heidelberg, Germany; 4 Deutsche Gesellschaft zur Bekämpfung der Krankheiten von Magen, Darm und Leber sowie von Störungen des Stoffwechsels und der Ernährung (Gastro-Liga) e. V., Giessen, Germany; 5 Klinik für Gastroenterologie, Allgemeines Krankenhaus Celle, Celle, Germany; 6 Universitätsklinik für Allgemein- und Viszeralchirurgie, Universitätsklinikum OWL, Campus Lippe, Detmold, Germany; 7 Chirurgische Klinik I, Allgemein-, Viszeral- und Thoraxchirurgie, Klinikum Darmstadt, Darmstadt, Germany; 8 Klinik für Internistische Onkologie und onkologische Palliativmedizin, Evang. Huyssensstiftung, Evang. Kliniken Essen-Mitte, Essen, Germany; 9 Klinik und Poliklinik für Strahlentherapie, Universitätsklinikum Essen, Essen, Germany; 10 Klinik für Gastroenterologie und Interventionelle Endoskopie, Krankenhaus Barmherzige Brüder, Regensburg, Germany; 11 Klinik für Hämatologie und Onkologie, Katholisches Marienkrankenhaus, Hamburg, Germany; 12 Klinik und Poliklinik für Allgemein-, Viszeral-, Tumor- und Transplantationschirurgie, Universitätsklinikum Köln, Köln, Germany; 13 Medizinische Klinik II/IV, Sana Klinikum Offenbach, Offenbach, Germany; 14 Deutsche Krebsgesellschaft e.V., Berlin, Germany; 15 Charité Comprehensive Cancer Center, Charité—Universitätsmedizin Berlin, Campus Charité Mitte, Berlin, Germany; 16 Conradia Radiologie, München, Germany; 17 Deutsche Gesellschaft für Ernährung e.V. (DGE), Bonn, Germany; 18 Klinik für Allgemein-, Viszeral- und Unfallchirurgie - Sektionsleiter Contilia Zentrum für Speiseröhrenerkrankungen, Elisabeth-Krankenhaus Essen, Essen, Germany; 19 Klinik und Poliklinik für Allgemeine Chirurgie Viszeral-, Thorax- und Gefäßchirurgie, Universitätsmedizin Greifswald, Greifswald, Germany; 20 Klinik für Allgemein-, Viszeral- und Kinderchirurgie, Universitätsklinikum Düsseldorf, Düsseldorf, Germany; 21 Klinik für Gastroenterologie, Rehabilitationsklinik Nahetal, Bad Kreuznach, Germany; 22 Med. Campus III, Klinik für Allgemein- und Viszeralchirurgie, Kepler Universitätsklinikum, Linz, Austria; 23 Zentrale Einrichtung Palliativmedizin, Universitätsklinikum Münster, Münster, Germany; 24 Universitäres Krebszentrum Leipzig, Universitätsklinikum Leipzig, Leipzig, Germany; 25 III. Medizinische Klinik, Klinikum rechts der Isar, TU München, München, Germany; 26 Medizinische Klinik und Poliklinik II, Universitätsklinikum Würzburg, Würzburg, Germany; 27 Medizinische Klinik II, Klinikum Ingolstadt, Ingolstadt, Germany; 28 Deutsche Gesellschaft für Chirurgie, Berlin, Germany; 29 Klinik und Poliklinik für Strahlentherapie, Universitätsklinikum Leipzig, Leipzig; 30 AWMF-Institut für Medizinisches Wissensmanagement, Berlin, Germany; 31 Institut für Ernährungs- und Lebensmittelwissenschaften, Universitätsklinikum Bonn, Bonn, Germany; 32 Klinik für Radioonkologie und Strahlentherapie, Universitätsmedizin Mainz, Mainz, Germany; 33 Klinik und Poliklinik für Nuklearmedizin, Universität zu Köln, Medizinische Fakultät und Universitätsklinik Köln, Köln, Germany; 34 Klinik für Innere Medizin I, Universitätsklinikum Ulm, Ulm, Germany; 35 Klinik für Innere Medizin – Hämatologie, Onkologie und Palliativmedizin, Klinikum im Friedrichshain, Berlin, Germany; 36 Medizinische Klinik I, Universitätsklinikum Frankfurt, Frankfurt, Germany; 37 Klinik für Allgemein-, Viszeral- und Onkologische Chirurgie, Klinikum St. Georg, Leipzig, Germany; 38 Deutsche Gesellschaft für Gastroenterologie, Verdauungs- und Stoffwechselkrankheiten e.V. (DGVS), Berlin, Germany; 39 Gastroenterologische Praxis am Kreiskrankenhaus Osterholz, Osterholz-Scharmbeck, Germany

**Keywords:** esophageal carcinoma, Barrett’s esophagus, perioperative chemotherapy, neoadjuvant chemoradiation, multimodal therapy, immune therapy

## Abstract

This guideline for the diagnosis and treatment of squamous-cell carcinoma and adenocarcinoma of the esophagus was developed and managed by the German Guideline Program in Oncology (GGPO) of the Association of the Scientific Medical Societies in Germany (AWMF), German Cancer Society (DKG), and German Cancer Aid (DKH). The guideline commission comprised multidisciplinary experts from various professional associations and organizations involved in the management of esophageal cancer, as well as a patient representative. Quality of the evidence is presented using Oxford evidence-based medicine system, and recommendations were graded in a formal consensus process using a recommendation grading scheme.

## Highlights

• This is the latest release of the evidence-based German guidelines for the diagnosis and treatment of esophageal cancers.
• The guidelines cover diagnosis, pathology, nutritional care, curative and palliative therapy, and psycho-oncology.
• Interdisciplinary approaches for curative therapy are discussed.

## Executive summary and list of recommendations

### Primary diagnosis and staging

**Recommendation 1 (Expert Consensus, EC):** All patients with new-onset dysphagia, gastrointestinal (GI) bleeding, recurrent aspiration, recurrent vomiting, dyspepsia, weight loss, and/or inappetence should be referred for early endoscopy [esophagogastrodudenoscopy (EGD)].

*Consensus strength: strong consensus*

**Recommendation** **2** **(EC):** Biopsies should be taken from all suspicious lesions during esophageal biopsy. In Barrett’s esophagus, additional four-quadrant biopsies should be taken. Suspect areas should be preserved separately and examined histopathologically.

*Consensus strength: strong consensus*

**Recommendation** **3** **(EC):** High-resolution video endoscopy has the highest sensitivity and specificity for the detection of neoplasms of the upper GI tract and should therefore be used as a standard diagnostic procedure.

*Consensus strength: strong consensus*

**Recommendation** **4:** Chromoendoscopy (Lugol’s solution) or computer-assisted digital (filter) procedures ought to be used in patients at risk for esophageal cancer (= anamnestic squamous-cell carcinoma of the mouth/nose/pharynx/bronchial system, esophagus).

*Level of evidence: 2a. Grade of recommendation: B.*

*Consensus strength: strong consensus*

**Recommendation** **5:** Chromoendoscopy as well as new computer-assisted digital (filter, contrast, and artificial intelligence) techniques ought to be used to improve the detection of dysplasia/early carcinoma of the esophagus.

*Level of evidence: 1b. Grade of recommendation: B*

*Consensus strength: strong consensus*

**Recommendation** **6:** Endoscopic ultrasound (EUS) ought to be part of the staging in patients with curative therapy intention.

*Level of evidence: 1b. Grade of recommendation: B*

*Consensus strength: strong consensus*

**Recommendation** **7:** The assessment of a ‘complete remission’ after neoadjuvant tumor treatment is not possible with sufficient sensitivity and specificity using the current examination methods [endoscopy/biopsy, EUS-fine needle biopsy (FNP), computed tomography (CT)/magnetic resonance imaging (MRI), and positron emission tomography (PET)/CT].

*Level of evidence2a. Consensus strength: strong consensus*

**Recommendation** **8** **(EC):** B-scan ultrasonography ought to be used as the first imaging modality to exclude liver metastases.

*Consensus strength: consensus*

**Recommendation** **9** **(EC):** B-scan ultrasonography of the neck can be used adjunctively to exclude cervical lymph node metastases for staging.

*Consensus strength: consensus*

**Recommendation 10** **(EC):** The determination of circulating tumor markers for diagnosis or therapy monitoring of esophageal cancer shall not be carried out.

*Consensus strength: strong consensus*

**Recommendation 11** (**EC):** The X-ray swallow shall not be used to diagnose esophageal cancer.

*Consensus strength: strong consensus*

**Recommendation 12** **(EC):** For the diagnosis of local tumor complications (fistulas), an X-ray examination with oral, water-soluble contrast medium can be carried out.

*Consensus strength: strong consensus*

**Recommendation 13:** Patients with newly diagnosed esophageal cancer ought to undergo multidetector CT (MDCT) of the neck/thorax and abdomen (with multiplanar reconstructions and wall distension with oral negative contrast) and additional intravenous (i.v.) contrast for primary staging.

*Level of evidence: 4. Grade of recommendation: B*

*Consensus strength: consensus*

**Recommendation 14:** In locally advanced tumors (cT 2-4 and cN+), a PET/CT examination may additionally be used for M-staging if the patient is potentially curatively treatable or the result has clinical consequences.

*Level of evidence: 1b. Grade of recommendation: 0*

*Consensus strength: strong consensus*

**Recommendation 15:** Flexible bronchoscopy ought to be carried out for locally advanced tumors in contact with the tracheobronchial system at the level of—or above—the carina.

*Level of evidence: 4. Grade of recommendation: B*

*Consensus strength: strong consensus*

**Recommendation 16** **(EC):** For staging of esophageal cancer, rigid endoscopy of the upper air and food passages should not be carried out.

*Consensus strength: consensus*

**Recommendation 17:** Diagnostic laparoscopy may be carried out for adenocarcinomas of the distal esophagus and esophagogastric junction (EGJ) to exclude metastases to the liver and/or peritoneum in advanced stages (especially in the case of cT3, cT4 category).

*Level of evidence: 1b. Grade of recommendation: 0*

*Consensus strength: strong consensus*

### Pathology

**Recommendation 18** **(EC):** Dysplasia/intraepithelial neoplasia (IEN) should be graded according to the current World Health Organization (WHO) classification into negative, unclear/questionable, low-grade dysplasia (LGD) or high-grade dysplasia (HGD).

*Consensus strength: strong consensus*

**Recommendation 19** **(EC):** In case of histological diagnosis of IEN/dysplasia in Barrett’s esophagus, the process of a competent (documented) pathological second opinion should be carried out in the sense of a four-eyes-principle. In case of dissent or uncertainty regarding the diagnosis of dysplasia, an external opinion should be obtained.

*Consensus strength: strong consensus*

**Recommendation** **20** **(EC):** The histopathological findings on the biopsy material shall include the following information:

• Type of neoplastic lesion [LGD/low-grade IEN (LGIEN), HGD/high-grade IEN (HGIEN), carcinoma], in particular whether an invasive carcinoma is present [in the case of HGD/HGIEN: classification on the biopsy material as Tis according to the International Union Against Cancer (Union Internationale Contre le Cancer; UICC)].
• Histological type according to WHO (in particular differentiation between squamous cell versus adenocarcinoma)
• For invasive adenocarcinomas: differentiation grade (grading) according to current WHO classification
• For lesions in the distal esophagus: is a goblet cell-containing Barrett’s mucosa present?

*Consensus strength: strong consensus*

**Recommendation** **21 (EC):** The histological classification and staging of esophageal cancers should be carried out according to the current WHO and TNM (tumor–node–metastasis) classification of the UICC. The pathological-anatomical assessment shall always be carried out completely and in a standardized form.

*Consensus strength: strong consensus*

**Recommendation** **22** **(EC):** The histopathological findings on local excisional data [endoscopic resection (ER)] shall include the following information:

• Size of the neoplastic lesion (in three dimensions if possible)
• Type of neoplastic lesion (LGD/LGIEN, HGD/HGIEN, carcinoma)—in particular, whether an invasive carcinoma is present (in the case of HGD/HGIEN: classification on the resectate as pTis according to UICC)
• In case of carcinoma detection: histological type according to WHO (especially differentiation squamous-cell versus adenocarcinoma, other rare types)
• In case of invasive adenocarcinoma: degree of differentiation (grading) according to current WHO classification
• Maximum depth of infiltration: pT1a (m1, m2, m3, m4)/pT1b (sm1, sm2, sm3) plus infiltration depth in μm (or higher pT category)
• Lymphatic vessel and/or vein invasion (L0 versus L1, V0 versus V1)
• Summary assessment of the risk of lymph node metastasis: low-risk versus high-risk resection margins with regard to the neoplasia (for ER in toto circular and basal resection margin for ‘piecemeal’ ER basal resection margin, as here the circular resection margin must usually be evaluated histopathologically as RX)

*Consensus strength: strong consensus*

**Recommendation** **23** **(EC):** The histopathological findings on surgical resected specimens shall include the following information:

• Size of the neoplastic lesion (in three dimensions if possible)
• Localization of the tumor center in relation to the EGJ and whether the tumor crosses the EGJ (if possible)
• Type of neoplastic lesion (LGD/LGIEN, HGD/HGIEN, carcinoma)—in particular, whether a carcinoma is present (for HGD/HGIEN: classification as pTis according to UICC)
• If carcinoma is detected: histological type according to current WHO classification (especially differentiation between squamous-cell versus adenocarcinoma, other rare types)
• Degree of differentiation (grading)
• Maximum depth of infiltration (pT)
• Lymphatic and/or venous invasion (L0 versus L1, V0 versus V1)
• Resection margins: oral, aboral, and circumferential: R0 versus R1
• Status of regional lymph nodes according to current UICC classification (pN) and ratio of number of affected and examined lymph nodes (…/…lymph nodes).

*Consensus strength: strong consensus*

**Recommendation** **24:** The histopathological findings on resected specimens ought to include additional statements on the regression score after preoperative therapy (neoadjuvant therapy).

*Level of evidence: 2b. Grade of recommendation: B*

*Consensus strength: strong consensus*

**Recommendation** **25** **(EC):** Before the use of palliative systemic tumor therapy in adenocarcinoma, the human epidermal growth factor receptor 2 (HER2) status should be determined as a positive predictive factor for a potential therapy with trastuzumab. The histopathological determination on tumor tissue should be carried out in a quality-assured manner.

*Consensus strength: strong consensus*

### Nutritional medical care

**Recommendation** **26** **(EC):** Nutritional medical care of patients with esophageal cancer should be an integral part of oncological diagnostics, therapy, and follow-up and should be an interdisciplinary task.

*Consensus strength: strong consensus*

### Curative therapy

**Recommendation** **27** **(EC):** Therapy recommendations should be made in an interdisciplinary tumor conference. Staging information, patient comorbidities, nutritional status, and patient preference should be taken into account as the basis for the therapy recommendation.

*Consensus strength: strong consensus*

**Recommendation** **28** **(EC):**

(i) If HGIEN or mucosal carcinoma (L0, V0, no ulceration, grading G1/G2) is detected in Barrett’s esophagus, endoscopic resection should be carried out, as this provides staging of the lesion with the question of deep infiltration in addition to therapy.
(ii) Therefore, an endoscopic complete resection with curative intention should be aimed for.
(iii) In patients with superficial submucosal infiltration of adenocarcinoma and no risk criteria (pT1sm1; <500 μm deep invasion, L0, V0, G1/2, <20 mm, no ulceration), endoscopic resection can be regarded as a sufficient alternative to surgery.
(iv) After successful resection of neoplasms in Barrett’s esophagus, the non-neoplastic Barrett’s mucosa should be thermally ablated to decrease the rate of metachronous neoplasms.

*Consensus strength: strong consensus*

**Recommendation** **29** **(EC):**

(i) If there is evidence of HGIEN or mucosal carcinoma (L0, V0, no ulceration, grading G1/G2, infiltration depth m1/m2) in the squamous epithelium, endoscopic en bloc resection should be attempted, as this will provide staging of the lesion with the question of deep infiltration in addition to therapy.
(ii) Therefore, resection with curative intention and R0 resection should be aimed for.

*Consensus strength: strong consensus*

**Recommendation** **30** **(EC):**

(i) If endoscopically nonlocalizable LGIEN is detected in Barrett’s esophagus and confirmed by a reference pathologist, follow-up visits shall occur at 6 months and then annually.
(ii) Radiofrequency ablation (RFA) of the entire Barrett’s segment to prevent progression of the LGIEN may be carried out.
(iii) If HGIEN cannot be localized endoscopically, an ablative procedure ought to be used.

*Consensus strength: strong consensus*

**Recommendation** **31** **(EC):** Local recurrence confined to the mucosa (crT1a cN0 cM0) after previous endoscopic resection of mucosal carcinoma in Barrett’s esophagus can be treated again endoscopically. If R0 resection is not possible with this, a surgical procedure should be chosen.

*Consensus strength: consensus*

**Recommendation** **32** **(EC):** After successful endoscopic therapy of HGIEN or early carcinoma, regular control endoscopies should be carried out (after 3 months, then every 6 months for 2 years, and then annually).

*Consensus strength: strong consensus*

**Recommendation** **33** **(EC):** Surgical treatment of esophageal tumors should be carried out in hospitals with at least 20 complex esophageal surgeries per year and a center for surgeons who are experienced in this type of procedure.

*Consensus strength: consensus*

**Recommendation** **34** **(EC):** Before planned esophagectomy, a risk analysis of important organ functions of the patient should be carried out. In case of functional inoperability despite oncological resectability, other therapeutic procedures should be used.

*Consensus strength: strong consensus*

**Recommendation** **35** **(EC):** The goal of surgical resection for squamous-cell carcinoma and adenocarcinoma is complete removal of the tumor (oral, aboral, and circumferential) and regional lymph nodes.

*Consensus strength: strong consensus*

**Recommendation** **36** **(EC):** In case of localization of the tumor

• in the EGJ [adenocarcinoma of the esophagogastric junction (AEG) type III], a transhiatal extended gastrectomy with distal esophageal resection should be carried out.
• in the EGJ (AEG type II), a transhiatal extended gastrectomy with distal esophagectomy, a right transthoracic subtotal esophagectomy, and alternatively a transhiatal abdomino-cervical subtotal esophagectomy can be carried out. Extensive infiltration of the lower esophagus favors esophagectomy more, whereas extensive infiltration of the subcardiac stomach favors gastrectomy more. In case of long-distance infiltration of both organs, a total esophagogastrectomy may be necessary.
• in the distal (including AEG type I) and middle thoracic esophagus, a right transthoracic subtotal esophagectomy should be carried out.
• in the upper thoracic esophagus, the extent of resection should be extended to the cervical region in order to maintain the safety distance to the oral region.
• in the cervical esophagus, the indication for a surgical procedure should be discussed in comparison with definitive radiochemotherapy, with a detailed benefit/risk assessment (see also Recommendation 58). The surgical procedure can be either a total esophagectomy or, in suitable cases, a cervical esophagectomy via a cervical approach with upper sternotomy.

*Consensus strength: consensus*

**Recommendation** **37** **(EC):** The extent of the lymphadenectomy depends on the location of the primary tumor, whereby three fields (abdominal, thoracic, and cervical) are distinguished. Two-field lymphadenectomy (abdominal, thoracic) is the standard approach.

*Consensus strength: strong consensus*

**Recommendation** **38** **(EC):** Reconstruction after transhiatal extended gastrectomy and distal esophagectomy should include end-to-side Roux-en-Y esophagojejunostomy. Subtotal esophagectomy should be followed by gastric elevation with high intrathoracic esophagogastrostomy, and total esophagectomy with cervical anastomosis. In case of unsuitable gastric interposition or after total esophagogastrectomy, colonic interposition should be carried out.

*Consensus strength: consensus*

**Recommendation** **39** **(EC):** Esophagectomy and reconstruction of the esophagus should be carried out minimally invasive or in combination with open procedures (hybrid technique) if there are no contraindications against this approach.

*Consensus strength: strong consensus*

**Recommendation** **40** **(EC):** In case of preoperative evidence of distant metastases, surgery shall not be carried out. In case of intraoperative findings of previously unknown, very limited distant metastases, these can be removed together with the primary tumor.

*Consensus strength: consensus*

**Recommendation** **41** **(EC):** Screening for malnutrition should be carried out as part of the preoperative risk stratification.

*Consensus strength: strong consensus*

**Recommendation** **42** **(EC):** Regardless of nutritional status, nutritional counseling should be offered concomitantly during neoadjuvant therapy.

*Consensus strength: strong consensus*

**Recommendation** **43** **(EC):** Patients with severe malnutrition, i.e. high metabolic risk, should receive nutritional therapy before surgery, even if surgery has to be postponed.

*Level of evidence: 1a. Grade of recommendation: A*

*Consensus strength: strong consensus*

**Recommendation** **44** **(EC):** After esophageal resection, enteral nutrition should be started within 24 h due to metabolic risk, if the patient’s clinical condition allows this. Parenteral supplementation may be recommended if <50% of the energy can be supplied by enteral means.

*Consensus strength: strong consensus*

**Recommendation** **45** **(EC):** In the case of an intraoperatively proven R1 resection, the possibility of a curative resection should first be examined independently of preoperative therapy. If this is not possible, post-operative radiochemotherapy should be carried out after discussion in the interdisciplinary tumor conference. In the case of a post-operatively detected R1 resection, radiochemotherapy should be given because the conditions for a postresection are unfavorable. In individual cases, a ‘wait and see’ strategy may be recommended.

*Consensus strength: strong consensus*

**Recommendation** **46** **(EC):** In case of a locoregional R2 resection, post-operative radiochemotherapy can be carried out after discussion in the interdisciplinary tumor conference.

*Consensus strength: strong consensus*

**Recommendation** **47** **(EC):** In case of an isolated local recurrence after curatively intended surgery, surgery can be carried out again after discussion in an interdisciplinary tumor conference. Careful evaluation of operability and resectability should be carried out by a treatment team experienced in esophageal surgery. Alternatively, radiochemotherapy should be offered if there has been no previous irradiation in the recurrent area or if there is sufficient normal tissue tolerance.

*Consensus strength: strong consensus*

**Recommendation** **48** **(EC):** If neoadjuvant therapy is planned, a risk analysis of important organ functions and a screening for malnutrition should be carried out in patients before starting therapy.

*Consensus strength: strong consensus*

**Recommendation** **49:** Preoperative radiotherapy alone may not be recommended in operable patients with resectable esophageal cancer.

*Level of evidence: 2a. Grade of recommendation: 0*

*Consensus strength: strong consensus*

**Recommendation** **50:** For localized adenocarcinomas of the esophagus and EGJ of category cT2, preoperative chemotherapy may be given and continued post-operatively.

*Level of evidence: 1b. Grade of recommendation: 0*

*Consensus strength: consensus*

**Recommendation** **51:** In operable patients with a locally advanced adenocarcinoma of the esophagus or EGJ (category cT3/T4 resectable or category cN1-3), perioperative chemotherapy or preoperative radiochemotherapy should be given.

*Level of evidence: 1a. Grade of recommendation: A*

*Consensus strength: consensus*

**Recommendation** **52:** The use of neoadjuvant chemotherapy alone without simultaneous radiotherapy for squamous-cell carcinoma of the esophagus cannot be recommended.

*Level of evidence: 1a. Grade of recommendation: B*

*Consensus strength: strong consensus*

**Recommendation** **53** **(EC):** In operable patients with cT2 squamous-cell carcinoma of the esophagus, preoperative radiochemotherapy followed by complete resection can be carried out.

*Consensus strength: strong consensus*

**Recommendation** **54:** In operable patients with a locally advanced squamous-cell carcinoma of the esophagus (category cT3/T4 resectable or category cN1-3), preoperative radiochemotherapy followed by complete resection should be carried out. See also Recommendation 59 ‘Indication for definitive radiochemotherapy’.

*Level of evidence: 1a. Grade of recommendation: A*

*Consensus strength: consensus*

**Recommendation** **55:** Self-expanding metal stents (SEMS) ought not to be used due to an increased complication rate with planned neoadjuvant radiochemotherapy or as a bridge to surgery.

*Level of evidence: 4. Grade of recommendation: B*

*Consensus strength: strong consensus*

**Recommendation** **56** **(EC):** After completion of a preoperative therapy, a new exclusion of distant metastases should be carried out. Restaging of the local findings can be carried out for planning the surgery.

*Consensus strength: strong consensus*

**Recommendation** **57** **(EC):** If clinical signs of tumor progression occur during preoperative therapy, symptom-based diagnostics should be carried out. If endoscopic or imaging evidence of local tumor progression is present, surgery should be carried out early.

*Consensus strength: strong consensus*

**Recommendation** **58** **(EC):** The clinical utility of [^18^F]2-fluoro-2-deoxy-D-glucose (FDG)–PET for response assessment of chemotherapy or radiochemotherapy before surgery is controversial, which is why FDG–PET/CT should not be routinely carried out in this setting.

*Consensus strength: strong consensus*

**Recommendation** **59:** Definitive radiochemotherapy should be given irrespective of the histological entity of the esophageal cancer if the tumor is deemed surgically/endoscopically unresectable at an interdisciplinary tumor conference or if a patient is functionally inoperable, or declines surgery after detailed explanation.

*Level of evidence: 1b. Grade of recommendation: A*

*Consensus strength: strong consensus*

**Recommendation** **60:** In patients with localized squamous-cell carcinoma of the cervical esophagus, definitive radiochemotherapy should be preferred over primary surgical resection.

*Consensus strength: strong consensus*

**Recommendation** **61:** In patients with resectable squamous-cell carcinoma of the intrathoracic esophagus of category cT3/cT4, definitive radiochemotherapy ought to be carried out as an alternative to surgical resection. See also Recommendation 54.

*Level of evidence: 1a. Grade of recommendation: B*

*Consensus strength: consensus*

**Recommendation** **62** **(EC):** In case of tumor persistence or local recurrence without distant metastases after radiochemotherapy, salvage surgery can be attempted with curative intent. Careful evaluation of operability and resectability should be carried out by a treatment team experienced in esophageal surgery.

*Consensus strength: strong consensus*

**Recommendation** **63** **(EC):** Antibodies and small molecules shall not be used in preoperative therapy.

*Consensus strength: consensus*

**Recommendation** **64:** After R0 resection of squamous-cell carcinoma, adjuvant radiotherapy or radiochemotherapy should not be carried out.

*Level of evidence: 1a (radiotherapy); 4 (radiochemotherapy). Grade of recommendation: B*

*Consensus strength: consensus*

**Recommendation** **65:** After primary R0 resection of a non-pretreated adenocarcinoma in the EGJ, adjuvant radiochemotherapy or chemotherapy may be carried out if there is an increased risk of recurrence (pN1-3).

*Level of evidence: 1b. Grade of recommendation: 0*

*Consensus strength: strong consensus*

**Recommendation** **66:** If residual tumor cells can still be detected histologically in the resection specimen (≥ypT1 or ≥ypN1) following neoadjuvant radiochemotherapy and R0 resection of squamous-cell carcinoma in the esophagus or adenocarcinoma in the esophagus or gastroesophageal (GE) junction, adjuvant immunotherapy with nivolumab ought to be carried out for 1 year.

*Level of evidence: 2. Grade of recommendation: B*

*Consensus strength: strong consensus*

**Recommendation** **67** **(EC):** Patients with esophageal cancer who have undergone potentially curative treatment should be offered structured follow-up care, provided that treatment decisions can be derived from this. In other cases, symptom-oriented follow-up care should be provided.

*Consensus strength: strong consensus*

**Recommendation** **68** **(EC):** In the first 6 months, regular follow-up checks of the nutritional status including dietary advice should be carried out. Supplementation of oral energy intake with drinkable solution or even tube feeding via an initially left fine-needle catheter jejunostomy can be recommended.

*Consensus strength: strong consensus*

**Recommendation** **69** **(EC):** Patients with esophageal cancer should be motivated—within their means—to engage in physical activity. After completing primary therapy, all patients capable of rehabilitation should be offered follow-up treatment. The rehabilitative therapy should include medical, nursing, and educational, training and psychosocial measures adapted to the individual rehabilitation needs. In order to reduce the fatigue syndrome caused by cancer or tumor therapy, endurance training should be carried out that is geared to the individual’s ability to cope with stress.

*Consensus strength: consensus*

### Palliative therapy

**Recommendation** **70:** All patients should be offered palliative care following the diagnosis of a non-curable cancer, regardless of whether tumor-specific therapy is undertaken.

*Level of evidence: 1. Grade of recommendation: A*

*Consensus strength: strong consensus*

**Recommendation** **71:** Patients with metastatic or locally advanced adenocarcinoma of the esophagus and EGJ that cannot be treated curatively should be offered systemic therapy. The therapeutic goal is to prolong survival and maintain quality of life.

*Level of evidence: 1a. Grade of recommendation: A*

*Consensus strength: strong consensus*

**Recommendation** **72** **(EC):** Before initiation of palliative systemic therapy in adenocarcinoma of the esophagus, HER2 status should be determined as a predictive factor for therapy with trastuzumab and programmed death-ligand 1 (PD-L1) combined positive score (CPS) should be determined as a predictive factor for therapy with an immune checkpoint inhibitor.

*Consensus strength: strong consensus*

**Recommendation** **73:** If HER2 status is negative and PD-L1 CPS < 5, a platinum (oxaliplatin or cisplatin)/fluoropyrimidine-containing two- or three-drug combination should be used.

*Level of evidence: 1a. Grade of recommendation: A*

*Consensus strength: strong consensus*

**Recommendation** **74:** In case of negative HER2 status and an elevated PD-L1 CPS cut-off value (for nivolumab PD-L1 CPS ≥ 5, for pembrolizumab PD-L1 CPS ≥ 10), a platinum (oxaliplatin or cisplatin)/fluoropyrimidine combination should be used together with one of the mentioned immune checkpoint inhibitors.

*Level of evidence: 1b. Grade of recommendation: A*

*Consensus strength: strong consensus*

**Recommendation** **75:** For HER2-overexpressing tumors [immunohistochemistry (IHC)3+ or IHC2+ and FISH+], first-line cisplatin/fluoropyrimidine-based chemotherapy should be supplemented with trastuzumab.

*Level of evidence: 2. Grade of recommendation: A*

*Consensus strength: strong consensus*

**Recommendation** **76** **(EC):** Before initiation of palliative systemic therapy of squamous-cell carcinoma, PD-L1 CPS should be determined as a predictive factor for therapy with an immune checkpoint inhibitor.

*Consensus strength: strong consensus*

**Recommendation** **77** **(EC):** Patients with metastatic or locally advanced squamous-cell carcinoma of the esophagus with a PD-L1 CPS < 10 and a PD-L1 tumor proportion score (TPS) < 1% that cannot be treated curatively may be offered palliative systemic chemotherapy. The therapeutic goal is to maintain quality of life. Here, a combination therapy of a platinum derivative with a fluoropyrimidine or a taxane can be used.

*Consensus strength: strong consensus*

**Recommendation** **78:** In patients with metastatic or locally advanced squamous-cell carcinoma of the esophagus that is not curatively treatable and has a PD-L1 CPS ≥ 10 or PD-L1 TPS ≥ 1%, platinum/fluoropyrimidine chemotherapy should be used in conjunction with an immune checkpoint inhibitor (pembrolizumab PD-L1 CPS ≥ 10, nivolumab PD-L1 TPS ≥ 1%).

*Level of evidence: 2. Grade of recommendation: A*

*Consensus strength: strong consensus*

**Recommendation** **79:** In patients with metastatic or locally advanced, non-curatively treatable squamous-cell carcinoma of the esophagus with a PD-L1 TPS ≥1%, the combination of nivolumab/ipilimumab can be used as the sole immunotherapy.

*Level of evidence: 2. Grade of recommendation: B*

*Consensus strength: strong consensus*

**Recommendation** **80:** In patients with metastatic or locally advanced adenocarcinoma of the esophagus not amenable to curative treatment and adequate general condition, second- and third-line systemic therapy ought to be given.

*Level of evidence: 1b. Grade of recommendation: B*

*Consensus strength: strong consensus*

**Recommendation** **81:** In patients with metastatic or locally advanced, non-curatively treatable adenocarcinoma of the esophagus and sufficient general condition, the high-frequency microsatellite instability (MSI-high) and/or mismatch repair deficiency (dMMR) status should be determined in the event of tumor progression under or recurrence after first-line therapy. Due to the high efficacy of immune checkpoint inhibitors in tumors with MSI-high or with a dMMR, these patients should be offered treatment with a checkpoint inhibitor after failure of first-line therapy if no immunotherapy has been used previously.

Cave: Consider off-label use

*Level of evidence: 2. Grade of recommendation: B*

*Consensus strength: consensus*

**Recommendation** **82:** Patients with metastatic or locally advanced squamous-cell carcinoma of the esophagus that cannot be treated curatively and sufficient general condition ought to receive second-line therapy with an immune checkpoint inhibitor, if there has been no previous immunotherapy.

*Level of evidence: 2. Grade of recommendation: B*

*Consensus strength: strong consensus*

**Recommendation** **83** **(EC):** Percutaneous radiotherapy of esophageal cancer—if necessary in combination with simultaneous chemotherapy—can be used for local symptoms (e.g. bleeding, stenosis, compression) as part of multidisciplinary care.

*Consensus strength: consensus*

**Recommendation** **84:** Palliative brachytherapy ought to be offered as part of the multidisciplinary care of patients with esophageal cancer to relieve dysphagia, in combination with percutaneous radiochemotherapy, or stent implantation when appropriate.

*Level of evidence: 1a. Grade of recommendation: B*

*Consensus strength: strong consensus*

**Recommendation** **85:** A self-expanding metal stent ought to be used for rapid relief of dysphagia in patients with esophageal cancer.

*Level of evidence: 1a. Grade of recommendation: B*

*Consensus strength: consensus*

**Recommendation** **86:** With an inserted SEMS, simultaneous percutaneous radiotherapy ought to be avoided because it is associated with an increased complication rate.

*Level of evidence: 4. Grade of recommendation: B*

*Consensus strength: strong consensus*

**Recommendation** **87** **(EC):** Intraluminal thermoablative therapy in patients with exophytic esophageal cancer in the palliative setting can be considered.

Additive brachytherapy or radiotherapy after local tumor ablation may prolong the dysphagia-free interval.

*Consensus strength: strong consensus*

### Psycho-oncology

**Recommendation 1 (EC):** The psycho-oncological care of patients with esophageal cancer should be an integral part of oncological diagnostics, therapy, and aftercare and pose an interdisciplinary task for all professional groups involved in oncology.

*Consensus strength: strong consensus*

## Scope and purpose

The German guideline for the diagnosis and treatment of esophageal cancer, first published in 2015, was managed by the German Guideline Program in Oncology (GGPO), covering the entire spectrum of prevention, diagnosis, and treatment of esophageal carcinoma, and aiming to enable standardization in prevention, diagnosis, therapy, palliation, and aftercare and thus pursue the goal of improving treatment outcomes. Editors of this guideline are from GGPO of the Association of the Scientific Medical Societies (AWMF), German Cancer Society (DKG), and the German Cancer Aid (DKH). The contents of the guideline are reviewed on the basis of up-to-date study data and publications, surveys on the quality and contents of the guideline, and feedback from the guideline group.

This guideline addresses physicians for internal medicine, gastroenterology, hematology and oncology, surgery, radiology, radiotherapy, pathology, nuclear medicine, and palliative medicine. Furthermore, this guideline is used to inform general care physicians and oncology professionals, professional groups involved in the care of patients with esophageal cancer, patient counselling organizations, self-help groups, as well as decision makers and cost bearers in the health care system. The approach of the guideline is interdisciplinary and cross-sectoral, as both inpatient/partial inpatient and outpatient care structures are included.

## Methodology

The methodological procedure for the development of the guideline is described in the guideline report, which is available online, e.g. the website of the German Guideline Program in Oncology (https://www.leitlinienprogramm-onkologie.de/leitlinien/oesophaguskarzinom/) and the website of the AWMF (http://www.awmf.org/). To update this guideline, a systematic literature search (search period September 2019 to March 2022) was carried out in PubMed and the Cochrane Library, with subsequent evidence independently assessed by the User-Group-Med. Guideline Development e.V./CGS Clinical Guideline Services. There are two types of recommendation in this guideline: evidenced-based recommendations and consensus-based recommendation. For all evidence-based recommendations, the evidence level of studies and grade of recommendation and consensus strength are described, and for the consensus-based recommendation, only the consensus strength is described.

### Level of evidence

To classify the risk of bias of the identified studies, the 2009 version of the Oxford Centre for Evidence-based Medicine system was used in this guideline (Supplementary Table S1, available at https://doi.org/10.1016/j.esmogo.2024.100112). The literature assessment for this guideline was carried out according to the evidence classification of the Oxford Centre for Evidence-based Medicine 2011 (Supplementary Table S2, available at https://doi.org/10.1016/j.esmogo.2024.100112).

### Grade of recommendation

The German Guideline Program in Oncology provides a formal consensus process for assigning recommendation grades by guideline authors, including moderated, nominal group processes or structured consensus conferences or DELPHI votes carried out by the AWMF. As part of these processes a patient representative, Barbara Kade, took part in the consensus conferences with her own voting rights. The respective votes are assigned to the recommendations according to the three categories presented in Table 1.

**Table 1:** Scheme of recommendation grading

Grade of recommendation	Description	Expression
A	Strong recommendation	Should/should not
B	Recommendation	Ought to/ought not to
0	Open recommendation	May/may not

The results of the respective votes (consensus strength) presented in this guideline are assigned according to Table 2.

**Table 2:** Consensus strength

Consensus strength	Percentage of consent
Strong consensus	>95% of those entitled to vote
Consensus	>75%-95% of those entitled to vote
Majority consensus	>50%-75% of those entitled to vote
Dissent	<50% of those entitled to vote

### Statement

Statements are presentations or explanations of specific facts or questions without an immediate call for action. They are adopted in accordance with the procedure for recommendations within the framework of a formal consensus process and can be based either on study results or on expert opinions (expert consensus).

### Expert consensus (EC)

EC refers to recommendations that are decided on the basis of expert consensus of the guideline group. No symbols or letters were used for the graduation of EC; the strength of recommendation for (expert) consensus-based recommendations results from the wording used (shall/should/can) according to the gradation in grade of recommendation.

## Background

Histopathologically, esophageal carcinoma comprises two main subtypes: adenocarcinoma and squamous-cell carcinoma. Projections from the Robert Koch Institute indicate an upward trend in esophageal cancer diagnoses, with an estimated 6100 newly diagnosed cases on men and 1800 cases on women, which corresponds to a share of 3.5% in men and 1.2% in women of all malignant neoplasms. Meanwhile, patients diagnosed with esophageal carcinoma had poor prognosis, with a relative 5-year survival rate of 22%-24%.^1^

The diagnosis and therapy of esophageal carcinoma is challenging and often includes multiple disciplines. In particular, the anatomical location of the esophagus in relation to the bronchial system and the lungs places considerable technical demands on the surgical and therapeutic procedure. Therefore, a multidisciplinary approach is required in order to guide patients to a stage-appropriate treatment after initial diagnosis. This includes in particular the decision as to whether patients should undergo surgery alone, be offered a combined approach of neoadjuvant preoperative radiochemotherapy plus surgery, or undergo radiochemotherapy alone. In addition, new diagnostic procedures (e.g. PET–CT) have been introduced into the staging diagnosis of esophageal cancer, the significance of which has not yet been clearly defined and established. On the other hand, a further challenge in treating patients with esophageal carcinoma, in particular for patients with squamous cell histology, is the fact that patients often have multiple comorbidities in relation to accompanying alcohol and tobacco consumption.

## Risk factors and prevention

**Figure 1:**
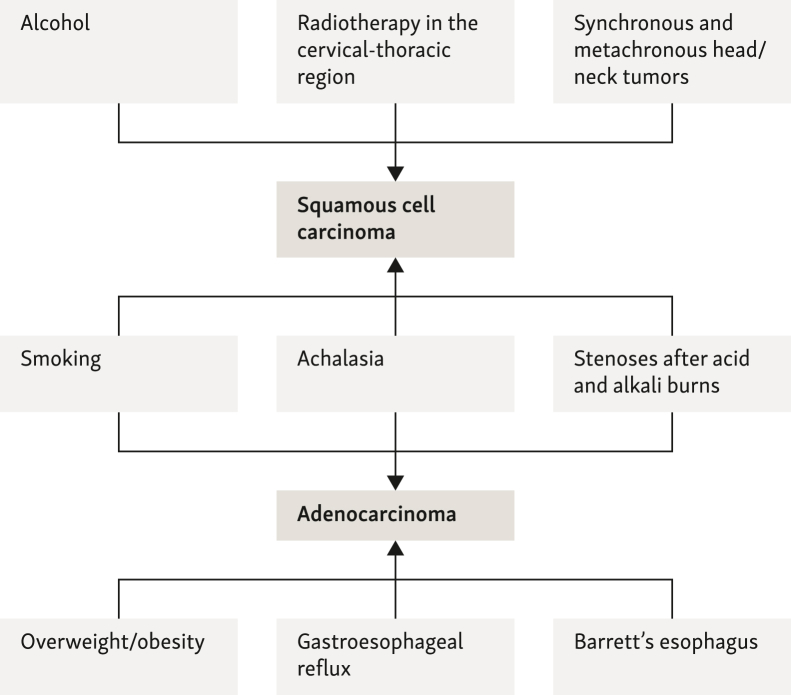
Known risk factors for the development of esophageal cancer.

The recognized risk factors for the development of esophageal cancer, as determined by EC in this guideline, have been summarized in Figure 1. Of note, some risk factors such as smoking,^2^, ^3^, ^4^, ^5^ achalasia,^6^, ^7^, ^8^ and stenoses after acid and alkali burns^9^ are shared by both squamous-cell carcinomas and adenocarcinomas of the esophagus and EGJ, while alcohol consumption,^10^, ^11^, ^12^, ^13^, ^14^ previous radiotherapy to the cervicothoracic region,^15^, ^16^, ^17^, ^18^, ^19^, ^20^ and synchronous and metachronous head and neck tumors^21^, ^22^, ^23^ are more specific for subsequent squamous-cell carcinoma of the esophagus. Meanwhile, the established risk factors for the development of esophageal adenocarcinoma include obesity,^14^^,^^24^, ^25^, ^26^, ^27^, ^28^ GE reflux disease,^29^, ^30^, ^31^, ^32^, ^33^, ^34^, ^35^ and Barrett’s esophagus.^36^, ^37^, ^38^, ^39^, ^40^, ^41^, ^42^

Yet in this guideline, no recommendation can be made for the prevention of the development of esophageal cancer with medication. Despite extensive research into the potential protective effects of acetylsalicylic acid and non-steroidal anti-inflammatory drugs (NSAIDs), findings remain controversial.^43^, ^44^, ^45^, ^46^, ^47^ No recommendation can be made for antioxidants as dietary supplements either.^48^ Physical exercise may be recommended from general health and preventive standpoints.^49^ The fact that a high intake of fruits and vegetables may contribute to reducing the risk of esophageal cancer has strong consensus.^50^ The relationship between the consumption of meat and meat products and the risk of esophageal cancer remains inconclusive, with conflicting results in studies.^51^, ^52^, ^53^, ^54^, ^55^

## Primary diagnosis and staging

### Primary diagnosis

**Recommendation 1 (EC):** All patients with new-onset dysphagia, GI bleeding, recurrent aspiration, recurrent vomiting, dyspepsia, weight loss, and/or inappetence should be referred for early endoscopy (EGD).

*Consensus strength: strong consensus*

**Recommendation 2 (EC):** Biopsies should be taken from all suspicious lesions during esophageal biopsy. In Barrett’s esophagus, additional four-quadrant biopsies should be taken. Suspect areas should be preserved separately and examined histopathologically.

*Consensus strength: strong consensus*

**Recommendation 3 (EC):** High-resolution video endoscopy has the highest sensitivity and specificity for the detection of neoplasms of the upper GI tract and should therefore be used as a standard diagnostic procedure.

*Consensus strength: strong consensus*

Dysphagia symptoms occur more frequently with increasing age.^56^ History and clinical examination often already give important clues to neurogenic, degenerative, pharyngeal or drug-triggered dysphagia, ENT disorders, recurrent aspiration/pneumonia, psychogenic syndromes, and other non-tumor-related dysphagic complaints. Examples of the latter causes are reflux esophagitis, hiatal hernias, rings, eosinophilic esophagitis, diverticula (including Zenker diverticula), subepithelial tumors (leiomyomas, GIST), or rare processes. Therefore, worldwide, as a ‘Good Clinical Practice’ (GCP) convention, patients with alarm symptoms (progressive/recurrent dysphagia, GI bleeding, weight loss, recurrent vomiting, recurrent aspiration pneumonia, inappetence) are advised to undergo high-resolution video endoscopy of the upper digestive tract with biopsy sampling at an early stage.

Advantages of high-resolution EGD include direct visualization and localization with sizing of suspicious lesions, biopsy sampling, surface analysis of seen changes, and the ability to use additional optical enhancement techniques [including HDTV resolution, magnification endoscopy, chromoendoscopy, computer-processed virtual chromoendoscopy, virtual surface-contrast enhancement, and, as the newest method, endoscopy with supporting techniques of artificial intelligence (AI)]. The chip-based standard endoscopy procedure is widely available today and has high safety.^57^

EGD has the highest sensitivity and specificity for the detection of neoplasms of all stages in the upper digestive tract. In the endoscopy report, the distance of the oral and aboral tumor margin in centimeters from the incisors, the endoscopic aspect of the tumor (e.g. Paris classification in cases of v.a. early cancer), and the localization of the EGJ and upper esophageal sphincter should be documented. In the case of highly stenosing, high-seated tumors, a thin-caliber special endoscope (4-5 mm diameter) may be helpful to avoid passage-related complications (perforation, bleeding).

### Advanced diagnostics

**Recommendation 4:** Chromoendoscopy (Lugol’s solution) or computer-assisted digital (filter) procedures ought to be used in patients at risk for esophageal cancer (= anamnestic squamous-cell carcinoma of the mouth/nose/pharynx/bronchial system, esophagus).

Level of evidence: 2a. Grade of recommendation: B

*Consensus strength: strong consensus*

**Recommendation 5:** Chromoendoscopy as well as new computer-assisted digital (filter, contrast, and AI) techniques ought to be used to improve the detection of dysplasia/early carcinoma of the esophagus.

*Level of evidence: 1b. Grade of recommendation: B*

*Consensus strength: strong consensus*

Video endoscopic examination with targeted biopsy is obligatory for the detection of esophageal cancer. In case of high-grade malignant stenosis that cannot be passed even with a pediatric gastroscope, forceps biopsy from the proximal tumor area is useful for carcinoma detection. If necessary, it can be combined with brush cytology. In squamous-cell carcinoma, topical staining with Lugol’s solution (iodine alkali) can increase the yield of neoplastic lesions by ∼30% (neoplastic tissue is low in glycogen and is therefore not stained).^58^, ^59^, ^60^, ^61^ Especially in high-risk patients (alcoholics, heavy smokers) and patients with already known squamous-cell carcinoma in the oropharynx (high risk of synchronous or metachronous lesions in the esophagus), chromoendoscopy is useful.^62^, ^63^, ^64^ In addition to malignant changes, inflammatory mucosal changes are also excluded from staining, and the specificity of chromoendoscopy with Lugol’s (iodine alkali) solution is consequently relatively low. Lugol staining is time-consuming and may cause esophageal spasms and pain. Patients with squamous-cell carcinoma of the head and neck and esophagus should be monitored during follow-up for the development of synchronous and metachronous secondary cancers.^65^ For this purpose, ancillary methods such as narrow-band imaging (NBI) and chromoendoscopy should be used liberally.

The computer-assisted, endoscopically applicable digital filter methods such as NBI,^66^—or digital ‘post-processing’ methods used by other manufacturers such as FICE (Fujinon Intelligent Chromoendoscopy) and iSCAN—aim to improve the visualization of surfaces or capillaries by digitally changing the color spectrum and thus use the neovascularization that occurs during carcinogenesis as a diagnostic criterion for detecting early neoplasia (‘virtual chromoendoscopy’). A meta-analysis revealed a 34% higher diagnostic yield with advanced imaging compared to white-light endoscopy. This improvement was also demonstrated for virtual chromoendoscopy in a subgroup analysis.^63^^,^^67^ Additionally, NBI was found to be more specific than Lugol’s staining with the same high sensitivity.^68^ The use of virtual chromoendoscopy procedures is therefore recommended for the detection of squamous-cell carcinoma.

The detection rates for precancerous lesions and for dysplasia/IEN are also improved in adenocarcinomas (AEG) and in dysplastic Barrett’s esophagus through the consistent use of classic chromoendoscopy and advanced additional digital procedures in endoscopy of the upper GI tract. These procedures should therefore be used generously—and routinely—in practice. These currently include the local application of acetic acid by spraying, classic chromoendoscopy with spraying of dyes (methylene blue, indigo carmine). Among the digital procedures, the virtual chromoendoscopy procedures (NBI, FICE, iSCAN) are particularly helpful, with the latest LED light endoscopes containing additional surface contrast enhancements (e.g. TXI mode), and individual centers also use sophisticated confocal laser endomicroscopy (CLE). Several studies show that both simple spray techniques and existing technical procedures for more precise/better contrasted mucosal surface observation improve the detection of early neoplasia in high-risk patients.^69^^,^^70^

The use of the NBI method can simplify the histological diagnosis of intestinal metaplasia in Barrett’s esophagus with few targeted biopsies compared to the high definition (HD) white-light method with randomized biopsies.^71^, ^72^, ^73^, ^74^ Multicenter randomized controlled trials (RCTs) are available for *in vivo* endomicroscopy (eCLE),^72^ which indicate that this method can significantly improve the detection of dysplasia and early carcinomas in Barrett’s esophagus compared to high definition white-light endoscopy (HDWLE).

The latest endoscopic add-on procedures are the first to use AI methods to improve the detection of mucosal lesions in Barrett’s esophagus and other lesions in the upper GI tract.^74^, ^75^, ^76^ By training the system with deep learning algorithms and using neural networks, the AI can independently detect dysplastic mucosal changes during endoscopy and display them to the examiner in real time. Some systems then also provide an assessment of the lesion found. RCT studies are not yet available for any procedure for squamous-cell carcinoma or Barrett’s dysplasia/adenocarcinoma. New study results are certainly to be expected in the near future; this procedure appears to be very promising. However, a concrete recommendation cannot be made at present.

### Staging of esophageal cancer

**Recommendation 6:** Endoscopic ultrasound (EUS) ought to be part of the staging in patients with curative therapy intention.

*Level of evidence: 1b. Grade of recommendation: B*

*Consensus strength: strong consensus*

**Figure 2:**
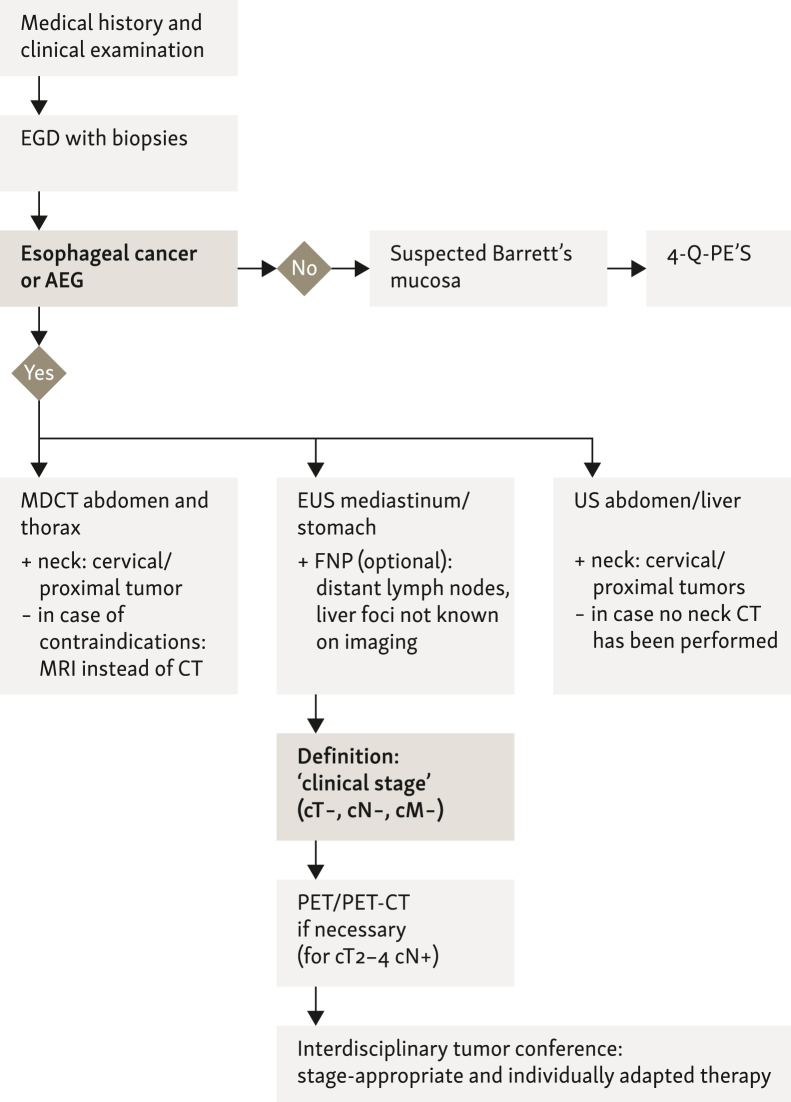
Algorithm for staging esophageal cancer. 4-Q-PE’S, 4 quadrant probe excision; AEG, adenocarcinoma of the esophagastric junction; CT, computed tomography; EGD, esophagogastrodudenoscopy; EUS, endoscopic ultrasound; FNP, fine needle biopsy; MDCT, multi-detector computed tomography; MRI, magnetic resonance imaging; PET, positron emission tomography; US, ultrasound.

The prognosis of an esophageal cancer patient correlates with local tumor infiltration depth (T category) and degree of lymphatic seeding (N category). Due to its high local spatial resolution, endosonography (EUS) has the highest accuracy for assessing local infiltration depth (T category) and is suitable for evaluating metastases in regional lymph nodes in squamous-cell carcinomas of the esophagus and in adenocarcinomas of the EGJ (AEG). Due to its relatively good accuracy—especially for a higher T category (sensitivity 91%-92%, specificity 94%-99%; Table 3)—and for local N-staging (Table 3), it is the endoscopic staging method of first choice (Figure 2). Consistent EUS tumor staging for esophageal cancer results in improved survival rates for patients.^77^^,^^78^ The selected studies of EUS for esophageal cancer are summarized in Table 3. However, it should be noted that using EUS for evaluating HGD and early carcinoma (pT1) in Barret’s esophagus can lead to over-staging (in ≤10%) and a relatively high false-negative rate.^79^

**Table 3:** Overview of staging results of endoscopic ultrasound (EUS) for the T and N categories of esophageal cancer (sensitivity/specificity by EUS/EUS-FNP)

Meta-analyses on EUS	Number of patients/studies	Sensitivity/specificity T category	Sensitivity/specificity N category
Thosani et al. (2012)^81^	1019/19	Early carcinomas^a^ (T1a/T1b): T1a: 85%/87% T1b: 86%/86%	n.s.
Puli et al. (2008)^80^	2585/49	T1: 82%/96% T2: 81%/94% T3: 91%/94% T4: 92%/97%	EUS: 85%/85% EUS-FNP: 97%/96%
Van Vliet et al. (2008)^82^	1841/31^b^	≥T2: 97% T4: -/99%	EUS: 80%/70% Celiac lymph nodes (formerly ‘M1a’): 85%/96%
Tranchemontagne (2009)^83^	n.s./n.s.	76%/67% Celiac lymph nodes (formerly ‘M1a’): 75%/94%	76%/67% Celiac lymph nodes (formerly ‘M1a’): 75%/94%
Luo et al. (2016)^78^		T1a: 84%/91% T1b: 81%/89% T4: 84%/96%	n.s.
Qumseya et al. (2018)^79^	Meta-analysis 11 studies only Barrett’s, HGD and early ca. (pT1)	Over-staging in 9.1% False negative: 9.2% Accuracy: 75%	n.s.

FNP, fine needle biopsy; HGD, high-grade dysplasia; n.s., not specified.

a In this literature work the accuracy is given for early carcinomas.

b Five studies on celiac lymph nodes.

The additional use of EUS-guided fine needle biopsy (FNP) can increase the nodal staging accuracy.^80^ EUS is—in potentially curative situations—complementary to CT as the basic diagnosis tool for staging esophageal cancer. It influences patient management and should be used generously to allow early selection of patients for endoscopic, primary surgical, primary neo-adjuvant, or palliative therapy. Comparative and interdisciplinary interpretation of EUS and CT results improve staging information over individual findings. The guideline group is therefore of the opinion that the limitations of the individual staging procedures can be overcome by the individually appropriate use of a combination of the available diagnostic procedures to achieve an overall good, preoperative clinical staging (Figure 2).

Limitations for EUS diagnostics exist on the one hand due to examiner dependency, on the other hand the distinguishability of small, mucosal processes is methodologically limited and EUS diagnostics is only possible to a limited extent in the case of highly stenosing tumors. EUS cannot differentiate between HGIEN and mucosal early cancers, but can be used in this setting before planned endoscopic mucosal resection (EMR) or endoscopic submucosal dissection (ESD) to exclude previously unsuspected, wall-spanning tumor processes in presumed early cancer and, if necessary, to diagnose regional lymph node metastases, which are very rare in early cancers. In up to 8%-12% of cases in practice, EUS can be used to identify previously undescribed ‘occult’ liver foci in the left liver lobe as well as other suspicious pathological findings such as ascites or pleural thickening, and in some cases can be further clarified using EUS-FNP. Optionally, ‘distant’ (tumor-remote) LN metastases can be confirmed paraaortally or parathyroidally by EUS-FNP, especially celiac LN metastases in squamous-cell carcinoma (see Figure 2). The use of contrast endosonography (KM-endosonography, CE-EUS) and ultrasound elastography are methods to improve the detection of occult and small metastases and, if necessary, to improve FNP; larger data collections are not yet available on this.

**Recommendation 7** (**EC**)**:** The assessment of a ‘complete remission’ after neoadjuvant tumor treatment is not possible with sufficient sensitivity and specificity using the current examination methods (endoscopy/biopsy, EUS-FNP, CT/MRI, and PET–CT).

*Level of evidence: 2a.*

*Consensus strength: strong consensus*

‘Restaging’ after successful neo-adjuvant therapy of esophageal cancer and AEG tumors is considerably limited in clinical practice, as tumors often show severe regression changes (necrosis, scars) post-therapeutically and inflammatory local factors may lead to false-positive results in all imaging modalities.^84^, ^85^, ^86^

**Recommendation 8:** B-scan ultrasonography ought to be used as the first imaging modality to exclude liver metastases.

*Consensus strength: consensus*

**Recommendation 9** (**EC**)**:** B-scan ultrasonography of the neck can be used adjunctively to exclude cervical lymph node metastases for staging.

*Consensus strength: consensus*

As a risk-free, noninvasive, available, and patient-accepted method, abdominal ultrasonography is the preferred initial imaging modality in staging diagnosis. B-scan ultrasonography has a sensitivity of 53%-81% and a specificity of 59%-98% in the detection of liver metastases, regardless of the underlying tumor disease.^87^ For GI tumors, the pooled sensitivity on sonographic detection of liver metastases is 66% [95% confidence interval (CI) 54% to 77%].^88^ More recent studies have reported a sensitivity of 77% and 81%,^89^^,^^90^ respectively, for the detection of liver metastases of GI tumors by B-scan ultrasonography. Contrast-enhanced sonography (CEUS) is comparable to CT and MRI in terms of specificity and sensitivity in the detection of liver metastases.^90^, ^91^, ^92^ The differentiation of metastases from primary malignant and benign tumors of the liver is achieved by contrast-enhanced sonography with an identical accuracy of >90% compared with CT or MRI.^93^, ^94^, ^95^, ^96^

Cervical lymph node metastasis occurs in 10%-28% of patients with esophageal cancer, especially when the tumor is cervical or highly intrathoracic.^97^ Detection of nonpalpable cervical lymph node metastases is possible both sonographically and with CT.^98^ B-scan sonography is equivalent or slightly superior to CT in the detection of cervical lymph node metastases.^99^, ^100^, ^101^ Ultrasound-guided fine-needle aspiration biopsy is additionally suitable for confirming metastatic lymph node involvement morphologically.^102^, ^103^, ^104^

**Recommendation 10** (**EC**)**:** The determination of circulating tumor markers for diagnosis or therapy monitoring of esophageal cancer shall not be carried out.

*Consensus strength: strong consensus*

Currently, there is no guideline- or data-based recommendation on the diagnostic use of circulating tumor markers for the primary diagnosis or monitoring of esophageal cancer.

**Recommendation 11** (**EC**)**:** The X-ray swallow shall not be used to diagnose esophageal cancer.

*Consensus strength: strong consensus*

**Recommendation 12** (**EC**)**:** For the diagnosis of local tumor complications (fistulas), an X-ray examination with oral, water-soluble contrast medium can be carried out.

*Consensus strength: strong consensus*

Routine chest X-ray examinations or pap swallow tests (X-ray contrast imaging of the esophagus) should not be carried out if staging by endoscopy, endosonography, and CT has been carried out because no new information is being obtained. The sensitivity of chest CT is 90% higher than chest X-ray at 68% in detecting pulmonary metastases from esophageal and cardiac carcinomas.^101^ X-ray contrast imaging of the esophagus can be an option in the imaging diagnosis of esophagotracheal fistulas, but again chest CT is preferable.^105^ This examination should be carried out with 50 ml of water-soluble contrast medium in cases of suspected fistula or perforation.

**Recommendation 13:** Patients with newly diagnosed esophageal cancer ought to undergo multidetector CT (MDCT) of the neck/thorax and abdomen (with multiplanar reconstructions and wall distension with oral negative contrast) and additional i.v. contrast for primary staging.

*Level of evidence: 4. Grade of recommendation: B*

*Consensus strength: consensus*

In order to be able to reconstruct in all spatial planes during MDCT, the minimum requirement for the scanner type is a multidetector CT with at least 16 lines (isotropic voxels). Typically, current generations of scanners use at least 64 lines to arrive at a spatial resolution of <1 mm to allow adequate T-staging. CT should always be carried out with wall distension,^106^ primarily negative, oral KM, ideally 1-1.5 l of water under spasmolysis in the following manner (so-called ‘hydro technique’) to improve T-staging as a protocol optimization: The patient should drink about 1 l of water over a period of about 25-40 min before the start of the examination. Immediately before the start of the CT scan, a further ∼150-200 ml of water is administered on the scanner table. Depending on the location of the tumor, a supine or prone position may be considered. The recommended slice thickness is 2.5-3 mm.

In addition to negative contrasting with water, i.v. administration of contrast medium containing iodine is obligatory. In addition to complete imaging of the esophagus, the liver should be imaged in the portal venous phase.^107^ Gas-forming granules (such as tartaric acid tartrate) can be administered for maximum wall distension, but usually oral water alone is sufficient. Several studies demonstrate that it is not necessary to carry out pelvic CT.^108^^,^^109^ Isolated pelvic metastases beyond the skeletal system in esophageal cancer are a rarity, so pelvic CT may not be necessary for dose reduction and cost savings.

The height localization and craniocaudal extension of the carcinoma is nowadays well possible by coronal and sagittal reformatting of the CT, which is why this typical question to the X-ray contrast imaging of the esophagus is obsolete. Due to the multislice technology in CT, longer scans of the entire esophagus from the cervical part to the esophagogastric junction are nowadays easily possible in a few seconds. It is therefore advisable to include the neck in the field of view, provided that no other examination, e.g. ultrasound, has been carried out. In this case, an additional ultrasound examination of the neck is no longer necessary. If, on the other hand, a CT of the abdomen and thorax is already available without the cervical parts of the esophagus, an additional ultrasound examination of the neck should be carried out. Regardless of the height localization of the carcinoma, a complete clarification of the entire esophagus should always be sought because of the possibility of distant metastases. The cranial border of the field of view is therefore marked by the upper jaw, and the scanning distance should always extend to the lower border of the liver.

T-staging: MDCT is limited in T-staging in the early stages. Nevertheless, sensitivities of 95% and a positive predictive value of 96% can be achieved using the ‘hydro technique’.^110^ T-staging could be carried out correctly in up to 76% of cases in this study.

N-staging: In lymph node staging, EUS is more sensitive (68%) than CT (33%), but less specific (58% versus 75%), except when combined with FNP, as stated above in Recommendation 6.^111^ In combination with EUS, a sensitivity of nodal involvement of 91% is achieved. A combination of PET–CT, MDCT, and EUS achieves the highest accuracy for determining LN status.^44^ The sensitivity for correct N-staging was reported with different limitations, e.g. 42% for EUS, 49% for PET, and 35% for CT.^112^ The specificity was 91%, 87%, and 93%, the correctness 66%, 68%, and 63%, respectively. There were no statistically significant differences. See Table 4 for further results.

**Table 4:** Direct comparison of the staging methods used in the detection of lymph node metastases

Method	Pooled sensitivity (95% CI)	Pooled specificity (95% CI)	Pooled accuracy (95% CI)
EUS-FNP	81% (0.76-0.85)	73% (0.63-0.80)	77% (0.72-0.81)
MDCT	54% (0.48-0.61)	87% (0.79-0.92)	65% (0.60-0.70)
FDG–PET	52% (0.44-0.60)	82% (0.65-0.92)	69% (0.60-0.77)

CI, confidence interval; EUS, endoscopic ultrasound; FDG–PET, fluorodeoxyglucose positron emission tomography; FNP, fine needle biopsy; MDCT, multidetector computed tomography.

Source reference:^80^^,^^81^^,^^83^

M-staging: CT has a significant variation in the accuracy of detecting a metastatic situation (sensitivity between 37% and 46%, specificity between 63% and 80%). Multiphasic CT of the liver increases sensitivity for the detection of liver metastases. However, hematogenous or peritoneal distant metastases may escape CT diagnosis, which is why sensitivities of only 46%-81% and specificities of 63%-82% are achieved here.^113^

### MRI for staging of esophageal cancer

In cases when CT cannot be carried out (e.g. contrast allergies), or as a complementary investigation to CT/EUS, MRI can be carried out. MRI is comparably accurate to CT for TNM staging,^114^ especially for tumors of the GE junction,^115^ but less accurate for pulmonary lesions.^116^ It is not superior to CT in any region.^117^ The diagnostic value for T-staging of esophageal cancer with MRI has steadily increased over the past few years. Currently, the available number of studies is still too small for definite conclusions.

In the near future, MRI has the potential to improve tumor delineation and real-time control for radiotherapy in tumor staging. The same is true for treatment response, especially in individualized tumor therapy. Furthermore, functional MRI as so-called diffusion MRI^118^ can provide valuable information beyond pure morphology. In addition, there are current developments in MRI technology to also image the esophageal wall more precisely and thus to better depict the depth infiltration—and thus further to improve T-staging if necessary.^119^ Regarding the role of MRI in response assessment after neoadjuvant therapy, reference may be made to the aforementioned recommendation 7.

**Recommendation 14:** In locally advanced tumors (cT 2-4 and cN+), a PET–CT examination may additionally be used for M-staging if the patient is potentially curatively treatable or the result has clinical consequences.

*Level of evidence: 1b. Grade of recommendation: 0*

*Consensus strength: strong consensus*

Diagnostic CT is the current standard for M-staging. The combination of PET with diagnostic CT has the highest sensitivity for M-staging and usually covers the trunk of the body (PET–CT: skull base to proximal femora and diagnostic CT of neck, thorax, and abdomen). The clinical value of FDG–PET or FDG–PET–CT for staging esophageal cancer was demonstrated.^112^^,^^120^, ^121^, ^122^, ^123^, ^124^ The Society of Thoracic Surgeons Guidelines on the Diagnosis and Staging of Patients with Esophageal Cancer^125^ recommends PET–CT as the best procedure for M-staging with a mean sensitivity of 71% and a mean specificity of 93%. Two meta-analyses investigated the role of PET–CT in the context of primary staging.^126^^,^^127^ Both confirm the known high diagnostic specificity but the low sensitivity, especially with regard to locoregional lymph node metastases. Although the false-negative rate was not insignificant, the detection of locoregional lymph node metastases on PET–CT nevertheless entails the clinical consequence of expanding the radiation volume or extending the lymph node dissection.

**Recommendation 15:** Flexible bronchoscopy ought to be carried out for locally advanced tumors in contact with the tracheobronchial system at the level of—or above—the carina.

*Level of evidence: 4. Grade of recommendation: B*

*Consensus strength: strong consensus*

**Recommendation 16** (**EC**)**:** For staging of esophageal cancer, rigid endoscopy of the upper air and food passages should not be carried out.

*Consensus strength: consensus*

Previous data suggest an association of squamous-cell esophageal cancer with synchronous neoplasms in the bronchial tree/oropharynx, but these are generally case series and observational studies.^128^ In patients with squamous-cell carcinoma of the esophagus, flexible diagnostic bronchoscopy may well be considered and used on the basis of these data—and the high level of safety of the procedure that is common today. In contrast, the previously common ‘pan-endoscopy’ of the entire accessible hollow systems in the head and the respiratory tract is unnecessary as a routine measure for the staging of esophageal cancer due to the lack of evidence.

If there is clinical suspicion of a tracheoesophageal or bronchoesophageal fistula and/or higher-grade infiltration of the tracheobronchial system, diagnostic bronchoscopy ± endobronchial ultrasound (EBUS)/biopsy may be clinically useful in individual cases.^129^^,^^130^ However, the evidence base for this is rather small, as systematic studies are lacking.

General bronchoscopy staging ± EBUS use can currently only be recommended in defined patients with locally advanced, (supra-) bifurcated squamous-cell carcinoma in whom the clinic/imaging suggests possible invasion into the tracheobronchial system and the findings would result in a clinical consequence.

### Diagnostic laparoscopy and thoracoscopy (staging)

**Recommendation 17:** Diagnostic laparoscopy may be carried out for adenocarcinomas of the distal esophagus and EGJ to exclude metastases to the liver and/or peritoneum in advanced stages (especially in the case of cT3, cT4 category).

*Level of evidence: 1b. Grade of recommendation: 0*

*Consensus strength: strong consensus*

Although there is evidence of diagnostic benefit in certain situations, the value of diagnostic laparoscopy and/or thoracoscopy in the staging of esophageal cancer and carcinoma of the EGJ is not clearly established. The guideline on the diagnosis and therapy of adenocarcinomas of the stomach and EGJ comments on diagnostic, pretherapeutic laparoscopy as follows: staging laparoscopy improves treatment decisions in locally advanced gastric cancer (especially cT3, cT4) and should be carried out before initiation of neoadjuvant therapy (recommendation grade B, level of evidence 1b).^131^ Due to its high diagnostic accuracy, it improves treatment decisions in locally advanced gastric cancer. This also applies to adenocarcinomas of the EGJ.^132^ In contrast, there is currently no evidence for the routine performance of diagnostic laparoscopy in distal adenocarcinoma of the esophagus (AEG type I according to Siewert), because here—compared to AEG II-III tumors—the incidence of peritoneal carcinomatosis is very rare.^132^ Diagnostic laparoscopy in AEG type I changed the management only in a negligible proportion of cases, so diagnostic laparoscopy in AEG type I cannot be routinely recommended.

## Pathology

**Recommendation 18 (EC):** Dysplasia/IEN should be graded according to the current WHO classification into negative, unclear/questionable, LGD or HGD.

*Consensus strength: strong consensus*

**Recommendation 19 (EC):** In case of histological diagnosis of IEN/dysplasia in Barrett’s esophagus, the process of a competent (documented) pathological second opinion should be carried out in the sense of a four-eyes-principle. In case of dissent or uncertainty regarding the diagnosis of dysplasia, an external opinion should be obtained.

*Consensus strength: strong consensus*

### Squamous epithelium

Intraepithelial neoplasia (IEN/dysplasia) of the squamous epithelium in the esophagus are immediate precursor lesions of esophageal squamous-cell carcinoma. The risk of carcinoma increases with the severity of IEN/dysplasia at: 2.9% for LGIEN/LGD, 28.3% for HGIEN/HGD, and 34.4% for carcinoma *in situ*.^133^ In addition, squamous-cell carcinoma can be detected in ∼20% of cases with IEN/dysplasia, which is usually located directly in the tumor margin. According to the current WHO classification, a two-tiered grading system should be used^134^; carcinoma *in situ* does not differ from HGD/HGIEN in terms of biological behavior and can therefore be subsumed in HGD/HGIEN.

### Barrett’s mucosa

Intraepithelial neoplasia/IEN or dysplasia in Barrett’s esophagus is defined as clearly neoplastic epithelium without evidence of infiltrative growth.^134^ Dysplasia (IEN) is classified as negative, indeterminate/questionable, or positive (low- or high-grade).^134^ Currently, evidence of dysplasia is the most valid marker of increased malignancy risk in Barrett’s esophagus. Therefore, the WHO classification should be mandatorily reported with any finding of Barrett’s mucosa.

Histologic dysplasia diagnosis in Barrett’s esophagus is subject to not inconsiderable interobserver variability, especially at the lower (regenerate versus LGD/LGIEN) and upper ends of the spectrum (HGD/HGIEN versus adenocarcinoma),^135^, ^136^, ^137^, ^138^, ^139^ but it is significantly higher in the assessment of endoscopic mucosal resections than in biopsies.^140^ Due to this pronounced interobserver variability, the German guideline on GE reflux disease/Barrett’s esophagus recommends that the diagnosis of dysplasia should be confirmed by a reference pathologist to reduce misdiagnosis.^42^ This diagnostic issue is also addressed in the European Society of Gastrointestinal Endoscopy (ESGE) guidelines and American College of Gastroenterology guidelines.^141^^,^^142^ Before endoscopic resection is carried out, ESGE recommends additional confirmation by an external pathologist.

**Recommendation 20 (EC):** The histopathological findings on the biopsy material shall include the following information:

• Type of neoplastic lesion (LGD/LGIEN, HGD/HGIEN, carcinoma), in particular whether an invasive carcinoma is present (in the case of HGD/HGIEN: classification on the biopsy material as Tis according to UICC).
• Histological type according to WHO (in particular differentiation between squamous-cell versus adenocarcinoma)
• For invasive adenocarcinomas: differentiation grade (grading) according to current WHO classification
• For lesions in the distal esophagus: is a goblet cell-containing Barrett’s mucosa present?

*Consensus strength: strong consensus*

The minimum number of biopsies required to reliably diagnose esophageal malignancy has not been defined to date. In our view, there is no evidence-based recommendation on the optimal number of forceps biopsies required to detect and diagnose Barrett’s carcinoma or squamous-cell carcinoma as confidently as possible. According to the results of Harrison et al.,^143^ one would postulate as follows: the more biopsies, the more likely the diagnosis. However, this is problematic as subsequent endoscopic ablation may be difficult after deep/large biopsies. Therefore, it has been proven in practice to take at least four mucosal biopsies from macroscopically suspicious areas.

Carcinoma in the esophagus is defined as neoplastic epithelial proliferation that infiltrates beyond the basement membrane into the mucosal stroma (intramucosal carcinoma) or the submucosa and deeper. Due to the different biological behavior, the distinction between squamous and adenocarcinoma is particularly clinically relevant. In the case of poorly differentiated or undifferentiated (G 3/4) tumors, immunohistological phenotyping should be carried out under this aspect, furthermore also for the identification of rare cancer types, such as neuroendocrine carcinoma, as well as for differentiation from secondary infiltration (p63, CK5/6, CK7, CK8/18, synaptophysin, chromogranin, TTF-1, etc.).

**Recommendation 21 (EC):** The histological classification and staging of esophageal cancers should be carried out according to the current WHO and TNM classification of the UICC. The pathological-anatomical assessment shall always be carried out completely and in a standardized form.

*Consensus strength: strong consensus*

The current TNM classification^144^ defines that a tumor whose center lies within 2 cm of the EGJ and extends into the esophagus is classified as an esophageal carcinoma. Tumors that involve the EGJ and whose center is located within the proximal 2 cm of the cardia (Siewert types I/II) are also classified according to the scheme for esophageal carcinomas. Tumors whose centers are located >2 cm from the EGJ (Siewert type III) are classified as gastric cancers (even when the EGJ is included) (Supplementary Tables S3-S5, available at https://doi.org/10.1016/j.esmogo.2024.100112).

### Anatomical subdivisions

Subdivision of each section of the esophagus and stomach is according to the ICD-O, topographic section,^145^^,^^146^ classification.

C15.0 Cervical esophagus

C15.3 Upper thoracic segment of intrathoracic esophagus

C15.4 Middle thoracic segment of intrathoracic esophagus

C15.5 Lower thoracic segment of intrathoracic esophagus

C16.0 Esophagogastric junction

### Regional lymph nodes

Regardless of the site of the primary tumor, the regional lymph nodes are those located in the lymphatic drainage area of the esophagus, including the celiac lymph nodes and paraesophageal lymph nodes of the neck, but not the supraclavicular lymph nodes. It should be noted that in esophageal cancers—and especially in carcinomas of the EGJ that grow into the stomach—the lymph nodes of the stomach are also counted as regional lymph nodes.^144^

**Recommendation 22 (EC):** The histopathological findings on local excisional data (ER) shall include the following information:

• Size of the neoplastic lesion (in three dimensions if possible)
• Type of neoplastic lesion (LGD/LGIEN, HGD/HGIEN, carcinoma)—in particular, whether an invasive carcinoma is present (in the case of HGD/HGIEN: classification on the resectate as pTis according to UICC)
• In case of carcinoma detection: histological type according to WHO (especially differentiation squamous-cell versus adenocarcinoma, other rare types)
• In case of invasive adenocarcinoma: degree of differentiation (grading) according to current WHO classification
• Maximum depth of infiltration: pT1a (m1, m2, m3, m4)/pT1b (sm1, sm2, sm3) plus infiltration depth in μm (or higher pT category)
• Lymphatic vessel and/or vein invasion (L0 versus L1, V0 versus V1)
• Summary assessment of the risk of lymph node metastasis: low-risk versus high-risk resection margins with regard to the neoplasia (for ER *in toto* circular and basal resection margin for ‘piecemeal’ ER basal resection margin, as here the circular resection margin must usually be evaluated histopathologically as RX)

*Consensus strength: strong consensus*

### Squamous-cell carcinoma

For the histopathological staging of early squamous-cell carcinoma of the esophagus, the depth of infiltration within the mucosa or submucosa should be determined according to the guidelines of the Japanese Society for Esophageal Diseases (m1-m3 or sm1-sm3), as there is a direct correlation between infiltration depth and the risk of locoregional lymph node metastasis (for m1: 0%, m2: 3.3%, m3: 12.2%; sm1: 26.5%, sm2: 35.8%, sm3: 45.9%).^147^ In a surgical collective, it was 0% for mucosal carcinomas versus 50% for submucosal infiltration: sm1: 33%, sm3: 78%.^148^

Therefore, the specified depth of infiltration (and probably the evidence of angioinvasion) is the most important parameter for the clinical question whether local tumor resection is sufficient or oncological resection is required. Moreover, grading has also been shown to be an independent risk factor.^148^

### Barrett’s adenocarcinoma

Since lymphatic and blood vessels are present in the lamina propria mucosae in the esophagus, lymph node metastases may occur even in adenocarcinomas confined to the mucosa,^149^ but this is much less frequent than in squamous-cell carcinoma. Here, too, there is a direct correlation to the microscopic infiltration depth, with a four-level subdivision of the mucosa due to the neo-muscularis mucosae typically occurring in Barrett’s esophagus: pT1a (m1, m2, m3, m4)/pT1b (sm1, sm2, sm3) plus infiltration depth in μm. However, the accuracy of the histopathological determination of the infiltration depth in layers m1-m4 and sm1-sm3 cannot always be precisely defined due to preparation inadequacies.

**Recommendation 23 (EC):** The histopathological findings on surgical resected specimens shall include the following information:

• Size of the neoplastic lesion (in three dimensions if possible)
• Localization of the tumor center in relation to the EGJ and whether the tumor crosses the EGJ (if possible)
• Type of neoplastic lesion (LGD/LGIEN, HGD/HGIEN, carcinoma)—in particular, whether a carcinoma is present (for HGD/HGIEN: classification as pTis according to UICC)
• If carcinoma is detected: histological type according to current WHO classification (especially differentiation between squamous-cell versus adenocarcinoma, other rare types)
• Degree of differentiation (grading)
• Maximum depth of infiltration (pT)
• Lymphatic and/or venous invasion (L0 versus L1, V0 versus V1)
• Resection margins: oral, aboral, and circumferential: R0 versus R1
• Status of regional lymph nodes according to current UICC classification (pN) and ratio of number of affected and examined lymph nodes (…/…lymph nodes)

*Consensus strength: strong consensus*

**Recommendation 24:** The histopathological findings on resected specimens ought to include additional statements on the regression score after preoperative therapy (neoadjuvant therapy).

*Level of evidence: 2b. Grade of recommendation: B*

*Consensus strength: strong consensus*

The indication of the localization of the tumor center in relation to the EGJ and whether the tumor crosses the EGJ can be difficult and is therefore not possible in all cases: the EGJ is defined differently, the only interpretable definition for pathological anatomy is the transition from squamous epithelium to cylindrical epithelium (definition according to the American Heritage Medical Dictionary).^150^ In carcinomas, especially AEG II according to Siewert,^151^ this borderline is often no longer recognizable in the pathohistological preparation because it is overgrown or replaced by tumor tissue. All other definitions,^147^, ^148^, ^149^ refer exclusively to the physiological boundary between the esophagus and the stomach, a boundary which is no longer detectable in the surgical specimen.

### Histological determination of tumor regression after neoadjuvant therapy

The tumor regression score is important and should be included in every report if preoperative therapy has been carried out. Patients with complete tumor regression show a significantly better course than patients with residual tumor. Therefore, to evaluate the effect of therapy, the entire tumor bed should be embedded and examined histologically. Various systems have been published for the classification of tumor regression grade, none of which has been generally accepted to date. In German-speaking countries, the regression gradings according to Mandard^152^ and Becker^153^, ^154^, ^155^ are frequently used (Table 5).

**Table 5:** Tumor regression score for adenocarcinomas according to Becker et al.^153^, ^154^, ^155^, ^156^

Degree of regression	Definition
1a	Complete regression
1b	Subtotal regression (1%-<10% residual tumor/tumor bed)
2	Partial regression (10%-50% residual tumor/tumor bed)
3	Low/no regression (>50% residual tumor/tumor bed)

The prognostic significance of complete and almost complete histopathological tumor regression after neoadjuvant therapy has been demonstrated in patients with squamous-cell and adenocarcinoma of the^157^^,^^158^ esophagus in several studies.^154^^,^^157^, ^158^, ^159^, ^160^ The ypTNM stage was the best predictor of survival in patients with locoregional esophageal cancer after neoadjuvant radiochemotherapy,^161^ where the nodal status has been identified as the prognostically dominant factor.^162^^,^^163^ In a cohort study, 400 of 584 operated patients with esophageal or transitional carcinoma received neoadjuvant chemotherapy.^164^ Tumor downstaging after neoadjuvant chemotherapy resulted in improved survival [hazard ratio (HR) 0.43, 95% CI 0.31-0.59] and was the strongest independent predictor of survival—stronger than clinical pretherapeutic tumor stage.

**Recommendation 25 (EC):** Before the use of palliative systemic tumor therapy in adenocarcinoma, the HER2 status should be determined as a positive predictive factor for a potential therapy with trastuzumab. The histopathological determination on tumor tissue should be carried out in a quality-assured manner.

*Consensus strength: strong consensus*

The results of a randomized phase III trial (ToGA trial) in patients with advanced carcinoma of the stomach (82%) or gastroesophageal junction (18%) showed that the addition of the HER2 antibody trastuzumab to standard chemotherapy significantly and clinically relevantly improved median survival from 11.1 to 13.8 months in patients with positive HER2 status.^165^

The results of the ToGA study lead to the fact that the drug therapy of metastatic gastric cancer and adenocarcinoma of the EGJ is defined for the first time on the basis of a predictive molecular-biological factor. For an indication for therapy with trastuzumab, HER2 positivity is defined (according to the European Medicines Agency guideline) as IHC3+ or IHC2+/FISH+. The rate of grade 3 or 4 adverse events did not differ between the two treatment groups (trastuzumab plus chemotherapy versus chemotherapy alone). Chemotherapy consisted of a combination of capecetabine/cisplatin or 5-fluouoracil/cisplatin.^165^ Compared with chemotherapy alone, the combination of trastuzumab and chemotherapy also improved quality of life.^166^

## Nutritional medical care

**Recommendation 26 (EC):** Nutritional medical care of patients with esophageal cancer should be an integral part of oncological diagnostics, therapy, and follow-up and should be an interdisciplinary task.

*Consensus strength: strong consensus*

Patients with esophageal cancer show weight loss,^167^ very early, not least due to mechanical obstruction of food passage. Here, the extent of dysphagia correlates with the nutritional deficit.^168^ The weight loss can already exceed 5% of the usual body weight in more than half of the patients on admission to hospital for surgery and exceed 10% of the usual body weight in 40%.^169^

Despite disease-associated weight loss, the body mass index (BMI) in patients with pre-existing obesity may be significantly above the WHO critical limit of 18.5 kg/m^2^ preoperatively. However, weight loss in itself implies a change in body composition, which entails a ‘metabolic risk’ that must be taken into account in patients before and during treatment, especially during surgery.^170^

Metabolically, we are talking about high-risk patients.^171^ This is even more true in the case of palliative therapy, where halting weight loss can increase treatment tolerance, reduce side-effects and fatigue, and improve quality of life (Non-Surgical Oncology Guideline). All these suggest a recording and observation of the nutritional status, starting with the inpatient admission or the first patient contact and requires local clear responsibilities with transparent clinical procedure standards (SOP) as for the indication for percutaneous endoscopic gastrostomy (PEG).^172^

## Curative intended therapy

### General therapy decision

**Recommendation 27 (EC):** Therapy recommendations should be made in an interdisciplinary tumor conference. Staging information, patient comorbidities, nutritional status, and patient preference should be taken into account as the basis for the therapy recommendation.

*Consensus strength: strong consensus*

The diagnosis and treatment of esophageal cancer is very demanding for all involved disciplines. On the one hand, this can be explained by the anatomical conditions—adjacency to the esophagus and to the bronchial system and the lung—but on the other hand also by the particularities of the patient collective to be treated (especially in the case of squamous-cell carcinoma). Therefore, a high degree of interdisciplinarity is required in order to assign patients to a stage-appropriate therapy after subtle diagnostics. This particularly includes deciding which patients should be treated by surgery alone and which patients should be treated by a combination of neoadjuvant radiochemotherapy plus surgery or which patients should be treated by definitive radiochemotherapy alone. The therapy recommendations to be made should therefore be decided in an interdisciplinary tumor conference involving the specialist disciplines involved.

### Endoscopic therapy

#### Endoscopic resection (ER) and local ablative procedures

**Recommendation 28 (EC):**

(i) If HGIEN or mucosal carcinoma (L0, V0, no ulceration, grading G1/G2) is detected in Barrett’s esophagus, endoscopic resection should be carried out, as this provides staging of the lesion with the question of deep infiltration in addition to therapy.
(ii) Therefore, an endoscopic complete resection with curative intention should be aimed for.
(iii) In patients with superficial submucosal infiltration of adenocarcinoma and no risk criteria (pT1sm1; <500 μm deep invasion, L0, V0, G1/2, <20 mm, no ulceration), endoscopic resection can be regarded as a sufficient alternative to surgery.
(iv) After successful resection of neoplasms in Barrett’s esophagus, the non-neoplastic Barrett’s mucosa should be thermally ablated to decrease the rate of metachronous neoplasms.

*Consensus strength: strong consensus*

The term ER includes both EMR, which is carried out using suction and cutting techniques, and ESD. In Germany, EMR is usually carried out using a ligation set or cap technique.

In the meantime, ER in the form of EMR has been established in many Western countries as a standard therapy procedure for HGIEN and mucosal adenocarcinomas.^98^^,^^173^^,^^174^ Numerous cohort studies have shown ER to be safe and effective, with lower morbidity and mortality than esophageal resection at the same curation rate.^175^, ^176^, ^177^, ^178^, ^179^, ^180^, ^181^, ^182^ Ideally, the ER ought to be used to remove the neoplastic lesion R0-en bloc to ensure accurate histologic staging.

By carefully processing the resection, the pathologist can make an accurate assessment of the depth of infiltration, the degree of differentiation, and the possible presence of lymphatic and vene infiltration. This information allows risk stratification so that either ER is the definitive therapeutic measure or the decision must be made to proceed with surgical therapy.

Esophageal resection should be considered whenever any of the following are present:

• Lymphatic (L1) or blood vene infiltration (V1)
• Poor differentiation (≥G3)
• Deep submucosal infiltration (≥500 μm)
• Tumor remnant at the basal resection margin (R1 basal)^183^, ^184^, ^185^, ^186^

In case of an incomplete ER or ‘piecemeal’ ER of a neoplastic lesion with evidence of tumor at the lateral resection margin (R1 lateral), another endoscopic therapy attempt can be made. During the next follow-up, a careful evaluation of the resection site and, if necessary, resection in the presence of neoplastic residues is indicated.^187^ The limit up to which neoplastic lesions can be resected using EMR R0-en bloc is ∼15 mm. Larger neoplastic lesions are resected using the piecemeal technique. However, a disadvantage of ‘piecemeal’ EMR is the higher recurrence rate than with resection of smaller lesions that can be resected en bloc.^177^

Endoscopic submucosal dissection can be used for en bloc resection of larger lesions. With this technique, an R0 resection, which is desirable from an oncological point of view, can be carried out regardless of lesion size. For squamous-cell carcinoma, numerous studies have shown ESD to be superior to EMR in terms of en bloc resection rate, curative resection rate, and local recurrence rate.^188^

However, little data exist for Barrett’s carcinoma. In a prospective study of 30 patients with HGIEN or focal Barrett’s carcinoma, complete resection with tumor-free resection margins was achieved in only 38.5% of patients despite ESD.^189^ Data from Japan show that R0 resection is possible in 85% of cases of Barrett’s esophagus.^190^ Apparently, the better data from Japan are related to the fact that the resections are larger and a higher safety distance to the side is chosen. Probst et al. showed that ESD for Barrett’s adenocarcinoma is also possible in Germany with an en bloc resection rate of 95.4% and an R0 resection rate of 84%.^191^

In patients with superficial submucosal infiltration of adenocarcinoma, endoscopic resection may be a sufficient alternative to surgery in selected cases. Manner et al. treated 66 patients with low-risk lesions (infiltration sm1, L0, V0, G1/2, no ulceration). Complete remission was achieved in 53 patients. After a median follow-up of 47 + 29.1 months, the estimated 5-year survival rate was 84%.^183^^,^^184^

One problem with EMR of Barrett’s neoplasms is the high rate of recurrence and metachronous lesions, reported as high as 30%.^192^ Reasons for this are that ‘piecemeal’ resections are by definition not R0 resections, but multifocal lesions in the Barrett’s esophagus may also be missed. Another problem is the existing genetic alterations in the Barrett’s mucosa, which cannot be eliminated by focal therapy of HGIEN or adenocarcinoma and lead to metachronous neoplasia in the course.

In a retrospective analysis, ablation [photodynamic therapy (PDT) or argon plasma coagulation (APC)] of the remaining, non-neoplastic Barrett’s mucosa after prior therapy of an HGIEN or mucosal carcinoma significantly reduced the rate of metachronous neoplasia.^177^ Meanwhile, several studies demonstrate that a two-stage approach consisting of initial ER followed by ablation of the non-neoplastic Barrett’s mucosa is most effective and has the fewest complications.^181^^,^^182^

Several procedures are available for ablation. Currently, only RFA and APC play a clinical role. PDT—used for many years as a standard procedure^193^, ^194^, ^195^—has almost been superseded due to its complex handling and its side-effects (stenosis and phototoxicity) and currently no longer plays a role.^196^, ^197^, ^198^ Long-term results on RFA show that recurrence of Barrett’s mucosa and neoplasia may occur in a relevant number of patients.^199^^,^^200^

Another ablation procedure that has become well established is APC therapy. Due to its ease of use, high availability, and low cost compared with RFA, APC therapy is mainly used for ablation of short-segment Barrett’s esophagus.^201^, ^202^, ^203^ The use in long-segment Barrett’s esophagus is certainly more complex and costlier than RFA. However, no prospective randomized studies comparing the two methods exist to date.

There are only sparse data on cryotherapy from the United States.^204^^,^^205^ In Germany, it is therefore not being used.

**Recommendation 29 (EC):**

(i) If there is evidence of HGIEN or mucosal carcinoma (L0, V0, no ulceration, grading G1/G2, infiltration depth m1/m2) in the squamous epithelium, endoscopic en bloc resection should be attempted, as this will provide staging of the lesion with the question of deep infiltration in addition to therapy.
(ii) Therefore, resection with curative intention and R0 resection should be aimed for.

*Consensus strength: strong consensus*

In analogy to Barrett’s adenocarcinoma, ER is the standard procedure for the treatment of mucosal carcinomas in the squamous epithelium. Only through the exact assessment of the pathologist is it possible to clarify whether an R0 resection or a low-risk situation is present and thus also whether the resection meets curative requirements.

In contrast to Barrett’s adenocarcinoma, there are numerous studies from Japan on the question of the resection procedure. Here, ESD has clear advantages over EMR. Especially for lesions >15 mm the rate of en bloc resections and curative R0-en bloc resections is significantly better. Ishihara et al. had a curative resection rate of 95% (20/21 patients) in lesions >15 mm with ESD, whereas with EMR the curative resection rate was significantly worse at 52% (16/31).^206^ Cao et al. also conclude in their meta-analysis that ESD is superior to EMR with regard to en bloc resection rate, R0-en bloc resection rate, and recurrences.^207^

**Recommendation 30 (EC):**

(i) If endoscopically nonlocalizable LGIEN is detected in Barrett’s esophagus and confirmed by a reference pathologist, follow-up visits shall occur at 6 months and then annually.
(ii) RFA of the entire Barrett’s segment to prevent progression of the LGIEN may be carried out.
(iii) If HGIEN cannot be localized endoscopically, an ablative procedure ought to be used.

LGIEN is a diagnosis with a certain rate of progression.^208^ Hvid-Jensen et al.,^39^ showed that patients with LGIEN have a fivefold higher risk of developing Barrett’s adenocarcinoma than patients without LGIEN. Curvers et al.,^67^ showed that if LGIEN was confirmed by a second pathologist, the incidence of developing HGIEN or carcinoma was 13.4% per patient per year. If the LGIEN was not confirmed and was declared a non-dysplastic Barrett’s esophagus, the incidence rate was 0.49%. However, Wani et al.,^37^ found no increased risk of progression in patients with LGIEN compared with patients without LGIEN.

Nevertheless, control endoscopies are recommended internationally after 6-12 months, during which all visible lesions are re-biopsied and additional quadrant biopsies are taken every 1-2 cm. If LGIEN is confirmed, either annual follow-up endoscopies or RFA are indicated.

RFA of Barrett’s epithelium with LGIEN is safe and effective. In a prospective randomized sham-controlled trial, RFA eliminated 95% of LGIEN.^196^ Interestingly, elimination also occurred in 26% in the sham therapy group. Phoa et al.,^209^ investigated whether patients with Barrett’s esophagus and LGIEN should be monitored or treated by RFA in a prospective randomized study. One hundred and thirty-six patients with LGIEN were randomized 1 : 1 to the RFA or control arm. Complete remission of LGIEN was achieved in 98% of patients in the treatment arm. In 37% of the patients in the observation group, no LGIEN could be found during the course. With regard to progression, there was a highly significant difference between the two groups after a median follow-up period of 21 months: progression occurred in 1.5% of patients in the therapy group and in 25% in the observation group. This difference suggests that RFA seems to be a good alternative for follow-up after 6 months. However, no conclusions on the long-term outcome can be drawn from this study.

Ablative procedures are considered the second-choice therapy after ER or surgery for early Barrett’s carcinoma. However, if areas with HGIEN changes are not macroscopically visible despite repeat examination at a center, targeted resection of the area cannot be carried out. In these cases, one must consider whether an ablative procedure, a resection of the entire Barrett’s area using the piecemeal technique or ESD, or a surgical procedure should be chosen. In such a case, the risk of overlooking an advanced neoplasia is very low, making undertreatment very unlikely. In this case, RFA is a sufficient and safe therapeutic option.^196^^,^^197^ Alternatively, ER can also be used successfully, especially in short-segment Barrett’s esophagus. This would ensure sufficient therapy as well as histological correlation with staging.^178^^,^^182^

The significance of ablative procedures in squamous-cell carcinoma is of secondary importance. PDT could gain some importance in the 1990s but plays almost no role today, at best as salvage therapy, if other procedures are contraindicated.^210^

Data on APC and RFA therapy are sparse. While the data on RFA are rather disappointing (50% CR for dysplasia in 20 patients), APC performs much better (CR of 95% in 19 patients).^211^, ^212^, ^213^ Cryotherapy has not been of any importance so far.

### Procedure for local recurrences after endoscopic therapy

**Recommendation 31 (EC):** Local recurrence confined to the mucosa (crT1a cN0 cM0) after previous endoscopic resection of mucosal carcinoma in Barrett’s esophagus can be treated again endoscopically. If R0 resection is not possible with this, a surgical procedure should be chosen.

*Consensus strength: consensus*

Pech et al. report on 1000 mucosal Barrett’s adenocarcinomas treated endoscopically. Metachronous or recurrent carcinomas occurred in 14.5% during follow-up of 56.6 ± 33.4 months.^187^ Of these 140 patients, 114 could be successfully treated again endoscopically. This concerns especially the cases with R1 situation at the lateral margin. In those cases where endoscopic therapy ultimately fails, surgery is still possible under curative conditions. This also includes the R1 situation at the basal resection margin.^176^^,^^177^^,^^214^

### Follow-up after endoscopic therapy

**Recommendation 32 (EC):** After successful endoscopic therapy of HGIEN or early carcinoma, regular control endoscopies should be carried out (after 3 months, then every 6 months for 2 years, and then annually).

*Consensus strength: strong consensus*

The problem of endoscopic therapy of Barrett’s neoplasia is the occurrence of recurrences or metachronous lesions, which amount up to 30%.^215^ Because recurrences are often amenable to repeat endoscopic therapy, follow-up should be close during the first 2 years after therapy. The evidence for this approach is low and is largely based on the practice carried out in studies.

### Surgical therapy

#### Hospital volume

**Recommendation 33 (EC):** Surgical treatment of esophageal tumors should be carried out in hospitals with at least 20 complex esophageal surgeries per year and a center for surgeons who are experienced in this type of procedure.

*Consensus strength: consensus*

A systematic review with meta-regression analysis showed that a minimum number of 20 complex esophagectomies per year is necessary to achieve a significant reduction in post-operative mortality.^216^ Current literature data confirm this minimum number and define low-volume clinics as those performing <20 esophagectomies per year.^217^ The number of cases per surgeon also plays a significant role.^218^ Furthermore, there is evidence that both ‘hospital volume’ and ‘surgeon volume’ have an impact on the prognosis of patients with esophageal cancer and that a higher long-term survival is achieved in hospitals with a high number of cases than in hospitals with a low number of cases.^217^^,^^219^ A clear correlation between the number of cases and post-operative mortality has now also been demonstrated nationwide for Germany.^220^

The expert commission of the guideline group recommends a minimum number of 20 complex esophageal operations per year and center. This is the most commonly used definition for ‘high-volume’ centers in the international literature. In the Netherlands, a quality program for esophageal and gastric surgery was launched in 2011—the Dutch Upper Gastrointestinal Cancer Audit (DUCA). The defined minimum volume of 20 esophageal resections per year and center led to a centralization of esophageal and gastric resections in specialized centers over the next 4 years, as well as to a demonstrable improvement in quality. The quality parameters were comparable with those of the Esophagectomy Complications Consensus Group (ECCG)—an international group of specialized centers that define the international benchmark.^221^

### Preoperative risk analysis

**Recommendation 34 (EC):** Before planned esophagectomy, a risk analysis of important organ functions of the patient should be carried out. In case of functional inoperability despite oncological resectability, other therapeutic procedures should be used.

*Consensus strength: strong consensus*

Due to the abdominothoracic approach and the necessary one-sided ventilation, esophagectomy represents a burden for the patient even in minimally invasive procedures. Therefore, a preoperative assessment of the patient’s functional capacity should be carried out. This should assess cardiac, respiratory, hepatic, and metabolic function as well as the patient’s ability to cooperate.^221^^,^^222^ Various studies have shown that when the risk of post-operative complications is systematically recorded, there is a good correlation with post-operative mortality and morbidity. For this purpose, various score systems are available that have been validated specifically for complex esophagectomy procedures, e.g. Cologne risk score,^221^ O-POSSUM (adapted POSSUM score for esophagectomy).^223^^,^^224^

### Surgical technique

#### Aim of the resection

**Recommendation 35 (EC):** The goal of surgical resection for squamous-cell carcinoma and adenocarcinoma is complete removal of the tumor (oral, aboral, and circumferential) and regional lymph nodes.

*Consensus strength: strong consensus*

### Resection extent

**Recommendation 36 (EC):** In case of localization of the tumor

• in the EGJ (AEG type III), a transhiatal extended gastrectomy with distal esophageal resection should be carried out.
• in the EGJ (AEG type II), a transhiatal extended gastrectomy with distal esophagectomy, a right transthoracic subtotal esophagectomy, and alternatively a transhiatal abdominocervical subtotal esophagectomy can be carried out. Extensive infiltration of the lower esophagus favors esophagectomy more, whereas extensive infiltration of the subcardiac stomach favors gastrectomy more. In case of long-distance infiltration of both organs, a total esophagogastrectomy may be necessary.
• in the distal (including AEG type I) and middle thoracic esophagus, a right transthoracic subtotal esophagectomy should be carried out.
• in the upper thoracic esophagus, the extent of resection should be extended to the cervical region in order to maintain the safety distance to the oral region.
• in the cervical esophagus, the indication for a surgical procedure should be discussed in comparison with definitive radiochemotherapy, with a detailed benefit/risk assessment (see also recommendation 58). The surgical procedure can be either a total esophagectomy or, in suitable cases, a cervical esophagectomy via a cervical approach with upper sternotomy.

*Consensus strength: consensus*

### Extent of lymphadenectomy

**Recommendation 37 (EC):** The extent of the lymphadenectomy depends on the location of the primary tumor, whereby three fields (abdominal, thoracic, and cervical) are distinguished. Two-field lymphadenectomy (abdominal, thoracic) is the standard approach.

*Consensus strength: strong consensus*

### Reconstruction

**Recommendation 38 (EC):** Reconstruction after transhiatal extended gastrectomy and distal esophagectomy should include end-to-side Roux-en-Y esophagojejunostomy. Subtotal esophagectomy should be followed by gastric elevation with high intrathoracic esophagogastrostomy and total esophagectomy with cervical anastomosis. In case of unsuitable gastric interposition or after total esophagogastrectomy, colonic interposition should be carried out.

*Consensus strength: consensus*

### Minimally invasive procedures (MIP)

**Recommendation 39 (EC):** Esophagectomy and reconstruction of the esophagus should be carried out minimally invasive or in combination with open procedures (hybrid technique) if there are no contraindications against this approach.

*Consensus strength: strong consensus*

### Carcinomas of the middle and distal third of the esophagus including AEG type I

Surgical resection represents a standardized therapy with curative intent for all potentially resectable esophageal cancers of the middle and distal third. An exception are carcinomas limited to the mucosa (T1a N0 M0), if they can be completely resected R0 endoscopically.^173^

The following arguments speak in favor of surgical therapy:

• It provides the best local control of the tumor.
• Locoregional freedom from recurrence is better for both adenocarcinoma and squamous-cell carcinoma than with definitive radiochemotherapy.^225^
• Radiochemotherapy is possible after surgery in the presence of local recurrence.
• Surgery after definitive radiochemotherapy is high-risk.^226^^,^^227^

The goal of curative resection is complete removal of the carcinoma orally, aborally, and circumferentially. In order to achieve tumor-free resection margins, i.e. an R0 resection, a proximal and distal safety margin of 2-4 cm must usually be maintained, except in mucosal carcinoma (T1a M0 N0).^228^

The extent of resection is determined by tumor location, TNM category, histology, and extent of any Barrett’s esophagus. Functional considerations of the reconstructing organ (stomach/colon) are also important in determining the extent of resection. The standard procedure is right transthoracic subtotal esophagectomy with resection of the proximal stomach and reconstruction with gastric elevation and high intrathoracic anastomosis.^229^, ^230^, ^231^ From a functional point of view, the high intrathoracic anastomosis with extensive transposition of the gastric interposition into the thoracic cavity results in reduced post-operative reflux compared to a lower anastomosis. In this case, the stomach would be half in the abdomen and half in the chest, so the positive abdominal pressure provokes reflux toward negative intrathoracic pressure.

With this subtotal esophagectomy and proximal gastric resection, a wide safety distance from the tumor is achieved for carcinomas of the middle and distal third orally and aborally. If the carcinoma is located in the upper thoracic esophagus, the extent of resection must be extended orally, possibly with cervical anastomosis. With en bloc resection, the necessary circular distance to the aorta and the tracheobronchial system can also be maintained for the targeted R0 resection. There is no firm evidence,^232^^,^^233^ for the principal removal of the azygos vein and thoracic duct together with the esophagus, but this may be necessary for reasons of local tumor resection in healthy tissue. Extension of en bloc resection to the pericardium or an infiltrated lobe of the lung is also reasonable if this can achieve R0 resection. T4a carcinomas with infiltration of the pleura, diaphragm, or pericardium are considered resectable, whereas T4b carcinomas with infiltration of the aorta, vertebral body, or trachea are considered non-resectable.^234^

The AEG classification^235^ is used to determine the extent of resection of adenocarcinomas of the EGJ. AEG type I carcinomas that are clearly esophageal cancers are treated with the described subtotal transthoracic esophagectomy. Resection of distal type I adenocarcinoma can alternatively be carried out by transhiatal (transmediastinal) blunt esophagectomy with cervical esophagogastrostomy. However, this procedure has two disadvantages. Firstly, lymphadenectomy is less radical.^236^ The prospective, randomized study by Omloo et al.^237^ demonstrated the resulting significantly worse prognosis of patients compared with transthoracic esophagectomy.^237^ This has been confirmed in the study by Kutup et al.^236^ with comparison groups formed according to the propensity score.^236^ The second disadvantage is that transhiatal esophagectomy always requires the creation of a cervical anastomosis. This has a higher rate of insufficiency than the intrathoracic anastomosis. Strictures requiring bougienage also occur more frequently as a result. In minimally invasive esophagectomy, patients with intrathoracic esophagogastrostomy have better functional outcomes than those with cervical anastomosis.^238^

Similar results are provided by the meta-analysis of 14 studies with 3468 patients comparing intrathoracic esophagogastrostomy for minimally invasive (MIS) Ivor-Lewis versus cervical anastomosis for MIS McKeown esophagectomy for carcinoma of the esophagus or cardia.^239^ Post-operative 30-day mortality as the main outcome criterion was not significantly different at 1% and 1.8%, respectively. The rate of anastomotic insufficiencies as the second main target criterion was significantly higher cervically (12.9%) than intrathoracically (5.7%) with, however, a lack of standardized recording.^240^^,^^241^ According to McKeown complications were also more frequent: the rate of pulmonary complications, recurrent paresis and anastomotic strictures, and blood loss and surgery lasted longer. Therefore, the authors favor Ivor-Lewis surgery when it is oncologically and functionally adequate in accordance with the above recommendations of this guideline.

Comparable results for cervical or intrathoracic anastomosis were obtained by the Dutch registry analysis of 2086 patients.^242^ The known criteria ASA III or higher score, chronic obstructive pulmonary disease, cardiac arrhythmias, diabetes mellitus, and proximally localized esophageal cancer are described as independent risk factors for anastomosis insufficiency.

In a prospective randomized trial of circular (*n* = 49) versus new triangular (*n* = 51) stapler anastomosis for cervical esophagogastrostomy, the triangular (19%) versus circular (17%) variant did not reduce the stricture rate 3 months post-operatively.^243^

### AEG type II and III

In AEG type II carcinomas, by definition with a tumor center of 1 cm above to 2 cm below the EGJ, the extent of resection depends on the exact topography and location of the main tumor mass. In type II carcinoma, transhiatally extended gastrectomy plus distal esophagectomy competes with esophagectomy and upper gastric resection. The latter is particularly indicated when transhiatally extended exposure of the esophagus is insufficient to achieve a safe R0 resection, or intraoperative frozen section at the esophageal margin shows infiltration. In the prospective randomized study by Omloo et al.,^237^ there was no prognostic improvement with transthoracic versus transhiatal resection for type II carcinomas.^237^ A prospective randomized study in type II/III has shown that in type II carcinomas, extending the extent of resection orally via a costal arch incision does not improve prognosis over transhiatal extended resection when R0 can be resected with the latter procedure.^244^ The 10-year results of this study, which are now available, confirm the results of 2006.^245^ The 5- and 10-year survival rates of all randomized patients with type II and III carcinoma with a maximum 3 cm invasion of the distal esophagus and cT2-4 category were 51% and 37% for the transhiatal approach and 37% and 24% for the approach with extension through the left costal arch, respectively. The log-rank test showed marginal differences between both groups: two-sided *P* = 0.060 and one-sided *P* = 0.970 with a HR for left thoracic versus transhiatal of 1.42.

For type III carcinoma, the prognosis of patients in this study was better even after the transhiatal procedure than after the extension via transection of the left costal arch. Therefore, for type III carcinomas that are considered gastric cancers, the procedure of choice is transhiatal extended gastrectomy with distal esophageal resection.^244^^,^^246^

In very advanced transitional tumors with extensive infiltration of the esophagus and stomach, total esophagogastrectomy with reconstruction by colonic interposition may be required to completely remove the tumor on all sides. Regarding limited resection of the EGJ for early carcinomas, see section Limited distal esophageal and proximal gastric resection.

### Squamous-cell carcinomas

In squamous-cell carcinomas, a distinction must be made between intrathoracic and cervical carcinomas (see below); in particular, the relationship to the trachea, tracheal bifurcation, and main bronchi is important. This close positional relationship requires, especially in advanced tumors, an exact dissection technique to spare the pars membranacea of the tracheobronchial tract combined with a radical clearance of the lymph nodes at the bifurcation and paratracheally. These requirements can only be fulfilled via transthoracal esophageal resection from the right. In a prospective randomized study of 286 patients with squamous-cell carcinoma of the middle or distal esophagus, overall or disease-free survival (DFS) was significantly higher with right thoracic approach (74% versus 62%) than after left thoracic incision (60% versus 52%).^247^ This difference is due to the better exposure of the esophagus and upper mediastinum from the right side in the more radical lymphadenectomy and was accordingly particularly pronounced in the subgroup with lymph node involvement in DFS. Transhiatal esophageal dissection results in a lower number of resected lymph nodes and a significantly worse prognosis than right thoracic approach.^236^

### Lymphadenectomy

**Figure 3:**
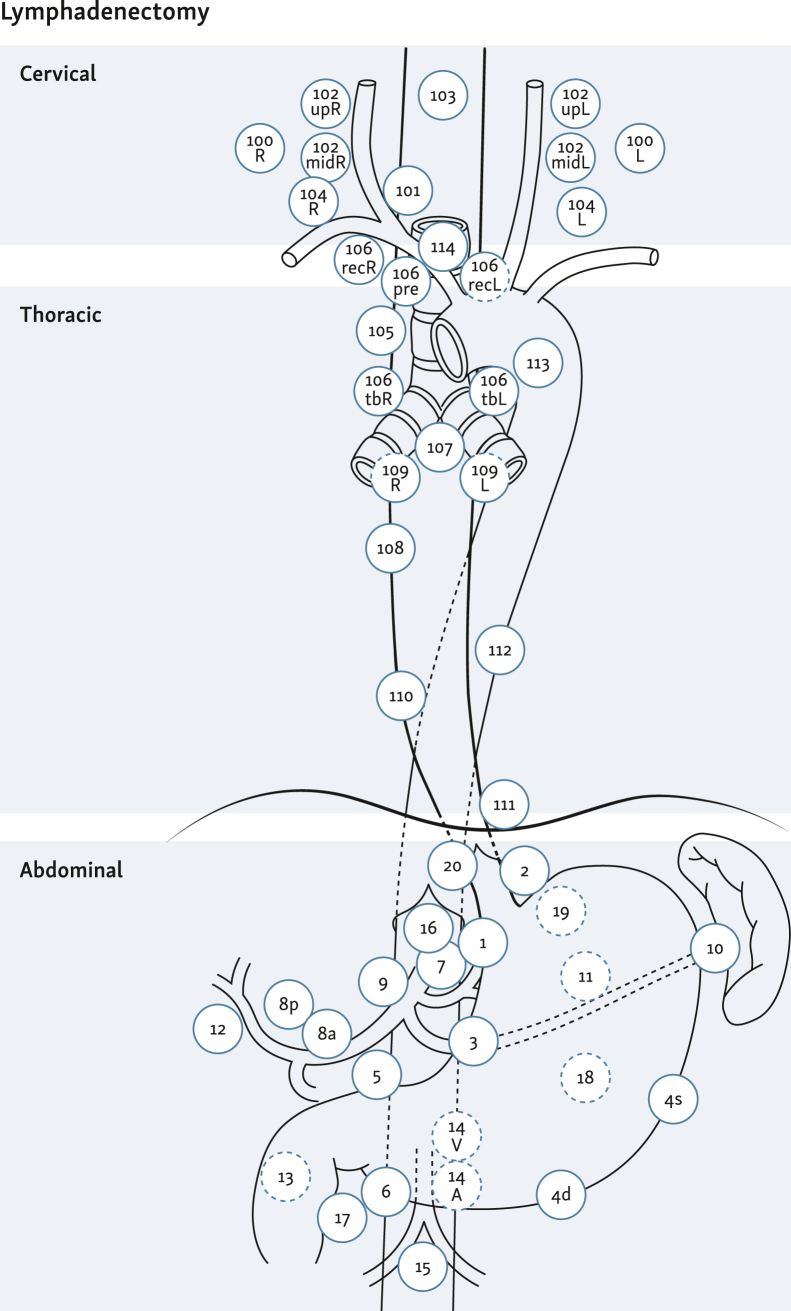
Illustration of the different fields of lymphadenectomy and the localization of lymph nodes in esophagectomy (from: Guidelines for Clinical and Pathologic Studies of Carcinoma of the Esophagus. *Jap Soc for Esophageal Diseases*, Tokyo 2001).

Two-field lymphadenectomy means lymph node excision of the thoracic and abdominal fields (Figure 3). Three-field lymphadenectomy also involves clearing out the lymph nodes of the cervical field (Figure 3). The regional lymph nodes in the upper abdomen and mediastinum are cleared out in esophagectomy not only directly peritumorally, but also in the corresponding lymphatic drainage area.^248^, ^249^, ^250^, ^251^

The removal of the regional lymph nodes, i.e. thoracic and abdominal—D2 and partial D1, possibly cervical—represents the standard for surgical treatment in curative intention. Removal of the cervical lymph nodes is not sufficiently justified in tumors of the middle and distal third of the esophagus.^250^, ^251^, ^252^ The currently accepted standard is the so-called two-field lymphadenectomy (LAD), i.e. of the thoracic and abdominal compartments. For cervical esophageal cancer, the third field, i.e. the cervical lymph nodes, should also be resected.

In thoracic and abdominal lymphadenectomy, >20 regional lymph nodes are usually removed and examined histopathologically. It has been shown in several studies that a median of 27 lymph nodes are removed in transthoracic esophagectomy and two-field LAD, whereas a median of only 17 are removed in transhiatal resection.^148^^,^^236^ According to a randomized study, significantly more lymph nodes (median 22 versus 18) are removed during transthoracic subtotal esophagectomy according to Ivor-Lewis with high intrathoracic esophagogastrostomy than during transthoracic Sweet esophagectomy with anastomosis in the middle mediastinum.^231^ Also, in a multicenter study with >1500 patients included, the median number of lymph nodes removed varied from 20 to 33,^249^ depending on the surgical approach.

In three further studies the prognostic relevance of the number of removed lymph nodes was investigated. In a multicenter study, a significant survival advantage was demonstrated with a cut-off value of 23 removed lymph nodes.^248^ A meta-analysis showed that an increasing number of extirpated lymph nodes improves overall and tumor-free survival after esophagectomy even after neoadjuvant therapy.^253^ Patients without lymph node metastases also have a better prognosis when >15 lymph nodes are removed than those with fewer resected lymph nodes.^148^

The number of lymph nodes removed and histologically examined may be lower as a result of neoadjuvant radiochemotherapy with adequate lymphadenectomy than without pretreatment, approximately by two examined lymph nodes,^254^^,^^255^ according to relevant studies.

Formally, for the pTNM classification of esophageal cancer, the removal and histological examination of at least seven regional lymph nodes is required to describe pN.^234^ For the pTNM classification of gastric cancer, a minimum of 16 lymph nodes is required for the pN0 category. Because there is a great deal of overlap between the stomach and esophagus, particularly in adenocarcinoma of the EGJ, the number of 16 or more lymph nodes seems much more representative for this determination of the N category. The formal requirements of the TNM committee, based on international consensus, represent minimum numbers for establishing the N classification. In surgical practice, the above numbers of 20-30 lymph nodes are realistic.^236^^,^^248^ Therefore, as a guide, the minimum number of 20 lymph nodes removed in two-field lymphadenectomy seems plausible. This statement should be evaluated as expert consensus.

### Limited distal esophageal and proximal gastric resection

In endoscopically non-removable T1 mucosal carcinoma, limited distal esophagectomy with proximal gastric resection can be carried out instead of subtotal esophagectomy after evaluation in a specialized high-volume center. This is especially true for AEG II and III, but also AEG I in UICC stage cT1N0M0 with low risk, i.e. G1, G2, L0, V0, intestinal type, without signet ring cells or non-poorly cohesive.^256^ Data from Japan indicate in AEG tumors with <4 cm diameter a very low rate of lymph node metastases of only 1%-2% at the distal stomach (lymph node stations 4, 5, and 6).^257^^,^^258^ The distal stomach could therefore be preserved just as it remains in subtotal esophagectomy and proximal gastric resection with gastric elevation, e.g. in AEG II, even two-thirds to three-quarters. Lymphadenectomy in compartment II can nevertheless be carried out completely, whereas it is limited in compartment I and is carried out only with resection of the upper part of the stomach.^259^ This limited resection of the EGJ should preferably be carried out abdominotranshiatally. If there is insufficient exposure, an abdominothoracic approach with separate right deep intercostal incision can also be carried out. Widening the abdominal approach through the left costal arch increases post-operative pain and often leads to costal healing problems. There are four reconstruction options for limited resection.^259^ Direct esophagogastrostomy is unfavorable because of the high rate of post-operative reflux symptoms and esophagitis. This problem can be reduced by a special technically complex anastomosis.^260^ However, this leads in about 20% to strictures requiring dilatation. The jejunum interposition with esophagojejunostomy and jejunogastrostomy (operation according to Merendino) is often burdened with problems due to reflux and gastric emptying disorders, especially if the vagus nerve cannot be preserved. Quality of life is not better after Merendino surgery than after subtotal esophagectomy—even worse in the first year post-operatively.^261^ Double-tract reconstruction is possibly better, as it is said to be associated with fewer reflux symptoms and, because of the two channels, rarely with voiding disorder.^262^ However, it is explicitly pointed out that there are no data for Europe proving a functional superiority of this technique. There are also no studies that demonstrate an improvement in quality of life compared with a minimally invasive, classic esophageal resection.

### Special case of cervical esophageal cancer

In cervical esophageal cancer, cervical esophageal resection can be carried out via a cervical approach with upper sternotomy in appropriate cases, and the regional lymph nodes are removed. Reconstruction is carried out via a free jejunal interposition with microvascular connection to venous and arterial neck vessels. Alternatively, a total esophagectomy can be carried out, with reconstruction by gastric elevation or colonic interposition.

Esophagectomy close to the superior esophageal sphincter is associated with a high rate of post-operative dysphagia and a tendency for aspiration. Furthermore, the rate of laryngeal recurrent nerve paresis is high. The long transposition distance of the gastric or colonic interposition often leads to vascularization disorders in the tip region of the elevated organ and thus to anastomosis insufficiency with subsequent strictures requiring bougienage. In free small bowel interposition with microvascular anastomoses, similar post-operative problems are to be expected, especially with thrombosis of the anastomosed vessels requiring revision.

Therefore, definitive radiochemotherapy of squamous-cell carcinoma in this location is an increasingly preferred treatment approach. The indication for surgical treatment of such highly seated tumors has to be considered very carefully due to the risks mentioned above and has to be discussed in detail with the patient including further therapy options.

### Reconstruction

After gastrectomy with distal esophageal resection, continuity is most commonly restored with a Roux-Y jejunum sling and end-to-side esophagojejunostomy transhiatally in the lower mediastinum.

The procedure of choice for reconstruction after subtotal esophagectomy is gastric elevation. Colon interposition may be considered as a second-choice procedure, especially in patients who have undergone previous gastric surgery. In exceptional cases, with very favorable vascular anatomy, the jejunum can be used as an interposition. As a rule, however, the vascular pedicle of a jejunum interposition to be obtained is too short. Intrathoracic anastomosis has the advantage over cervical esophagogastrostomy of a lower rate of insufficiency and better post-operative swallowing function.^229^^,^^230^^,^^263^ Because cervical anastomosis also requires additional access to be created, it should only be carried out if an esophageal resection to the neck must be carried out for oncologic reasons. The best route for reconstruction is the posterior mediastinum. In special conditions, such as high risk of recurrence in the posterior mediastinum or planned radiation to the posterior mediastinum, or in the two-stage procedure with delayed reconstruction, the anterior mediastinum can be used retrosternally to bring the interpositioner up.

The reconstruction route through the anterior mediastinum is ∼5 cm longer than in the posterior mediastinum and always requires a cervical anastomosis. There is a tendency for siphon formation at the upper thoracic aperture during reconstruction in the anterior mediastinum retrosternally. This often results in a worse swallowing function. Reconstruction antesternally subcutaneously is reserved for a few exceptional cases.

The most common procedure is single-stage resection and reconstruction. In patients with limited functional capacity or questionable vascularization of the prepared gastric interposition, a two-stage reconstruction may be useful.^230^^,^^264^

### Pyloroplasty

Functional pylorospasm with gastric retention often occurs post-operatively due to the transection of the vagus nerve during esophagectomy. The formation of the gastric interposition can be carried out with or without pyloroplasty. However, there is no clear evidence for carrying out pyloroplasty during gastric pull-up in either meta-analysis or systematic reviews.^265^^,^^266^ To reduce post-operative gastric emptying disorders due to functional pylorospasm, endoscopic balloon dilatation of the pylorus to 3 cm can be carried out prophylactically preoperatively.^267^

### Hiatoplasty

Because of the possible development of paragastric hiatal hernias and the danger of incarceration of the small intestine or colon, a hiatoplasty should be carried out after gastric retraction or colon interposition if the diaphragm is too wide.^268^, ^269^, ^270^

In laparoscopic gastrolysis it is advantageous to present the suture at the diaphragm and to move it unknotted to the thoracic region. This suture can be adapted to the diameter of the interposition device and knotted in the thoracic part after gastric elevation.

### Minimally invasive esophagectomy (MIS)

Esophagectomy and reconstruction with gastric elevation can be carried out minimally invasively. The following variants are possible: totally minimally invasive with laparoscopic gastrolysis and thoracoscopic esophagectomy, with either intrathoracic or cervical esophagogastrostomy; hybrid procedures; laparoscopic gastrolysis and transthoracic open esophagectomy, with either intrathoracic or cervical anastomosis; open gastrolysis and thoracoscopic esophagectomy, with either intrathoracic or cervical anastomosis; and laparoscopic gastrolysis and transhiatal esophagectomy with cervical esophagogastrostomy.

Studies such as the TIME trial and MIRO trial have shown that MSI can lead to lower rates of pulmonary complications, blood loss, shorter hospital stays, and better short-term quality of life compared to open surgery.^246^^,^^263^^,^^271^^,^^272^ However, both MIS and open surgery face challenges such as asymptomatic anastomotic stenoses, which may require further endoscopic dilatation, sometimes multiple times. The TIME trial revealed no significant difference in 3-year long-term survival rates between MSI and open surgery.^273^ Long-term results of non-randomized studies of MIS esophagectomy do not allow an accurate assessment of prognosis compared to open procedures.^274^, ^275^, ^276^

The MIRO trial favored hybrid procedures for lower morbidity, pulmonary complications, and post-operative Clavien-Dindo score II-IV. The results are confirmed by a retrospective study with propensity matching and a meta-analysis of 2397 patients.^277^^,^^278^ Another meta-analysis suggests lower 5-year all-cause mortality with minimally invasive techniques.^279^

The comparison between hybrid technique and total MIS is described in the study by Bonavina et al.,^280^ which found no significant differences in post-operative morbidity/mortality, number of resected lymph nodes, R0 resection rate, and 1-year survival between both groups. Only the duration of surgery was significantly longer with MIS.

Overall, MIS or hybrid technique shows advantages over the open procedure. This concerns the reduction of post-operative, in particular pulmonary, complications, a trend towards improved prognosis, and a medium-term improvement in health-related quality of life. It should be noted, however, that individual series of total MIS show increased rates of anastomotic insufficiency, recurrent hiatal hernia, and post-operative hiatal hernia.^271^^,^^275^^,^^281^, ^282^, ^283^ If there are no contraindications to the MIS approach such as adhesions due to previous surgery or foreseeable difficulties of resectability, the hybrid or total minimally invasive technique ought to be used for esophagectomy.

### Perioperative complications

Due to the high coincidence of concomitant diseases and the frequent presence of various risk factors in patients with esophageal cancer on the one hand, and the invasiveness of radical surgical therapy on the other, perioperative complications cannot be completely avoided. Prevention, early detection, and appropriate consistent management of complications after esophagectomy represent the most important starting points for the successful treatment of esophageal cancer.

The Esophagectomy Complications Consensus Group (ECCG) has standardized the accurate documentation of esophagectomy complications to make data from different centers comparable.^241^ The first over 2700 documented cases show a good reproducibility of this documentation system for recording complications and reliable data from large centers.^241^ This documentation system with exact definition and graduation of complications, created by experts, is recommended. It is used to review one’s own results in morbidity and mortality conferences and for quality assurance.

### Procedure for oligometastasis

**Recommendation 40 (EC):** In case of preoperative evidence of distant metastases, surgery shall not be carried out. In case of intraoperative findings of previously unknown, very limited distant metastases, these can be removed together with the primary tumor.

*Consensus strength: consensus*

Esophagectomy and simultaneous resection of distant metastases do not confer a prognostic advantage according to small retrospective series. Therefore, esophagectomy should be avoided in preoperative M1 situation. However, if a small resectable metastasis, e.g. to the lung or liver, is discovered intraoperatively, metastasis resection can be carried out within the scope of esophagectomy for histological confirmation on the one hand and for complete tumor resection on the other hand.^284^^,^^285^ The results of the AIO-FLOT 3 trial changed the therapeutic approach for the small group of limited metastatic gastric and AEG tumors, which appeared to benefit from neoadjuvant chemotherapy followed by resection of tumor and metastases.^286^ This approach is currently being tested in an ongoing, prospective randomized trial (AIO-FOLT5).^287^ A review of literature published up to 2019 summarized the limited evidence known to date from 14 retrospective studies on the management of oligometastatic esophageal cancer.^288^ Lung and liver are the most common localizations, and an individualized therapeutic approach is advocated by most studies.

### Perioperative nutrition

**Recommendation 41 (EC):** Screening for malnutrition should be carried out as part of the preoperative risk stratification.

*Consensus strength: strong consensus*

The impact of nutritional status on post-operative complication rates after major surgery has been widely demonstrated.^288^^,^^289^ Despite disease-associated weight loss, the BMI in patients with obesity preoperatively may be well above the WHO critical threshold of 18.5 kg/m^2^. Weight loss itself represents a change in body composition that entails a ‘metabolic risk’ that must be considered in patients when planning major tumor surgery.^170^

The disease-associated metabolic risk can be easily assessed with the Nutritional Risk Score (NRS).^290^ This screening tool has also been validated for surgical patients in recent studies.^291^^,^^292^ In a large cohort study, decreased food intake in the week before hospital admission as recorded by the NRS has even been shown to be the sole risk predictor in abdominal surgical patients.^293^ For older surgical patients (aged >65 years) in a systematic review of 15 studies from 1998 to 2008, only weight loss and the reduction in serum albumin associated with poor nutritional status could be found as predictive parameters of post-operative morbidity.^170^

According to the European Society for Clinical Nutrition and Metabolism (ESPEN),^288^ high metabolic risk is defined as the presence of any of the following criteria:

• Weight loss >10%-15% within 6 months
• BMI <18.5 kg/m^2^
• Subjective global assessment (SGA) grade C or NRS >5
• Serum albumin <30 g/l (if liver or kidney insufficiency is excluded)

A preoperatively low serum albumin level is often associated with a poor nutritional status and is a prognostic factor for post-operative complications and mortality.^294^^,^^295^ However, it is not possible to directly increase albumin levels through nutritional therapy.^296^

**Recommendation 42 (EC):** Regardless of nutritional status, nutritional counseling should be offered concomitantly during neoadjuvant therapy.

Consensus strength: strong consensus

Intensive perioperative nutrition therapy (INS) in patients with esophageal cancer during neoadjuvant therapy demonstrated its favorable impacts, including lower major complication rate and shorter hospital stay in a Dutch prospective study.^297^ These findings underscore the benefit of intensive perioperative nutritional medical co-treatment especially in major tumor operations.

The indication for PEG during neoadjuvant therapy should be made extremely critically and only in consultation with the surgeon in charge, especially in cases of planned gastric tube formation. In this case, direct puncture should be attempted to prevent tumor cells from being carried away by the pull-through technique.^298^ More favorable is the placement of a fine-needle catheter jejunostomy, e.g. laparoscopically, which can also be left in place during resection.

**Recommendation 43 (EC):** Patients with severe malnutrition i.e. high metabolic risk should receive nutritional therapy before surgery, even if surgery has to be postponed.

Level of evidence: 1a. Grade of recommendation: A

Consensus strength: strong consensus

Many patients do not meet their energy needs preoperatively through their normal diet; these patients should be motivated to take an oral nutritional supplement regardless of their nutritional status. Although the benefits of an immunomodulating diet have been shown in many studies for patients with tumors in the GI tract, the data for patients with esophageal cancer are inconclusive.^299^^,^^300^

Postponing surgery with the aim of nutritional conditioning is only justified in patients with a high metabolic risk, i.e. severe malnutrition. Primarily, enteral nutrition should always be given preference—if possible before hospital admission—in order to avoid nosocomial infection (guideline adaptation gastric cancer and clinical nutrition).^246^^,^^301^

Parenteral nutrition results in recovery of physiological function and total body protein within 7 days. However, further significant improvement may be expected in the second week.^302^ A recent Cochrane analysis of preoperative parenteral nutrition in GI surgery patients showed significant reduction in complications from 45% to 28%.^303^ These authors discussed a bias as three of the included studies were >20 years old. However, two important studies^304^^,^^305^ with positive results were not included. When parenteral nutrition in patients with weight loss >10% was carried out for 10 days preoperatively and continued post-operatively for 9 days, the complication rate was significantly lower by 30% with a tendency to reduce mortality.^304^

**Recommendation 44 (EC):** After esophageal resection, enteral nutrition should be started within 24 h due to metabolic risk, if the patient’s clinical condition allows this. Parenteral supplementation may be recommended if <50% of the energy can be supplied by enteral means.

Consensus strength: strong consensus

Post-operative medical nutrition therapy (artificial nutrition) is indicated in patients with malnutrition and those at nutritional risk. It should be also initiated if it is anticipated that the patient will be unable to eat for >5 days. The indication also exists for patients expected to have low oral intake and who cannot maintain >50% of the recommended amount of energy for >7 days (guideline adaptation: clinical nutrition surgery).^306^^,^^307^

The benefits of post-operative early enteral nutrition starting within 24 h have been shown in several meta-analyses in terms of reduction in infection rates, hospital length of stay, and even mortality.^307^, ^308^, ^309^ The results of a randomized study have shown that after minimally invasive esophagectomy, an early oral nutrition can be given without risk for anastomosis insufficiency.^310^ However, most patients will not cover calorie requirement for a longer time period. It may be recommended to place a feeding tube intraoperatively either duodenally/jejunally or as a fine-needle catheter jejunostomy (FCJ).^301^

For patients after esophageal resection, an observational study showed significant benefits of safe longer-term enteral feeding via FCJ, especially in the presence of anastomotic problems.^289^ The complication rate of FCJ was low (1.5%).^311^ For early enteral feeding after esophagectomy and catheter-associated complications, a prospective RCT found no significant difference between the use of a nasoduodenal tube or an FCJ.^312^

Because nasojejunal and nasoduodenal tubes dislodge significantly more frequently, earlier^313^^,^^314^ FCJ is superior for long-term enteral nutrition.^314^

Parenteral supplementation of enteral intake should be carried out if <50% of the energy can be supplied by the enteral route.^306^

### Procedure for R1/R2 resection

**Recommendation 45 (EC):** In the case of an intraoperatively proven R1 resection, the possibility of a curative resection should first be examined independently of preoperative therapy. A retrospective study indicates that adjuvant chemo(radio)therapy improves survival and reduces distant recurrence in patients with R1 resection, but without affecting locoregional recurrence.^315^ If this is not possible, post-operative radiochemotherapy should be carried out after discussion in the interdisciplinary tumor conference. In the case of a post-operatively detected R1 resection, radiochemotherapy should be given because the conditions for a postresection are unfavorable. In individual cases, a ‘wait and see’ strategy may be recommended.

*Consensus strength: strong consensus*

**Recommendation 46 (EC):** In case of a locoregional R2 resection, post-operative radiochemotherapy can be carried out after discussion in the interdisciplinary tumor conference.

*Consensus strength: strong consensus*

It is considered certain that an incomplete removal of the primary tumor will result in tumor recurrence, i.e. to be considered palliative. This is undisputed for R2 resection (macroscopic tumor remnant remains at surgery). Recent retrospective data from Great Britain, however, cast doubt on the fact that an R1 resection with microscopic tumor remnant in the so-called circumferential resection margin is undoubtedly associated with a poor prognosis.^316^^,^^317^ Nevertheless, every surgeon will strive to achieve an R0 resection not only longitudinally but also circumferentially, if necessary by means of frozen-section diagnostics.

If this has not been achieved primarily, a follow-up resection is usually discussed in the treatment team in case of a longitudinal R1 resection, although this is usually associated with (too) much effort or (too) high risk in esophageal cancer. The optimal approach after incomplete resection for esophageal cancer is not well supported by data. Prospective studies from the 1980s suggest that additive radiotherapy may improve local recurrence rates but not relapse-free or even overall survival (OS).^318^^,^^319^ There are no robust data on additive chemotherapy.

### Procedure for local recurrence after surgery

**Recommendation 47 (EC):** In case of an isolated local recurrence after curatively intended surgery, surgery can be carried out again after discussion in an interdisciplinary tumor conference. Careful evaluation of operability and resectability should be carried out by a treatment team experienced in esophageal surgery. Alternatively, radiochemotherapy should be offered if there has been no previous irradiation in the recurrent area or if there is sufficient normal tissue tolerance.

*Consensus strength: strong consensus*

Prospective and retrospective studies have shown that isolated local recurrences or lymph node metastases of squamous-cell carcinoma of the esophagus after surgery without preoperative or post-operative radiochemotherapy have a curative chance with definitive radiotherapy or radiochemotherapy in recurrence.^320^, ^321^, ^322^ In this situation, simultaneous radiochemotherapy was also found to be more effective. The 5-year survival rates after salvage radiochemotherapy ranged from 14% to 45% in the studies mentioned, so they do not differ significantly from those in primary therapy. Anastomotic recurrences had a worse prognosis than regional lymph node metastases in two of the studies. As the number of affected regional lymph node metastases in the recurrence increases, the prognosis decreases. The time interval between primary therapy and recurrence therapy was not important in any of the studies.

Radiochemotherapy is also considered for isolated local recurrences of adenocarcinoma of the esophagus. Long-term survivors were also observed here.^323^^,^^324^ In some cases, patients received salvage radiochemotherapy after previous neoadjuvant radiochemotherapy in recurrence. Isolated local recurrences are rare after neoadjuvant therapy and resection at <15%.^324^^,^^325^ In the study by Sudo et al.,^324^ 5.4% of 518 patients with locally advanced adenocarcinoma of the EGJ had isolated local recurrence after neoadjuvant radiochemotherapy and surgery. Recurrence treatment was highly dependent on individual patient risk factors and recurrence location. Forty-four percent of patients with isolated locoregional recurrence received radiochemotherapy for recurrences outside the initial irradiated volumes. The 3-year survival rate of these patients was 25% after recurrence therapy. The majority of patients developed distant metastases during the course.

Retrospective studies discussed the potential benefits of surgical resection for isolated recurrent lesions after radical surgery, showing that well selected patients may benefit from re-resection.^326^^,^^327^ However, no prospective comparative data are available. We recommend personalized decision making in such scenarios after discussion in interdisciplinary tumor conferences.

### Multimodal therapy concepts

**Recommendation 48 (EC):** If neoadjuvant therapy is planned, a risk analysis of important organ functions and a screening for malnutrition should be carried out in patients before starting therapy.

*Consensus strength: strong consensus*

Before any therapy start, the expected benefit must be weighed against the possible risk. Therefore, the risk of a therapy should be assessed beforehand by examining the organ functions. Similarly, malnutrition is associated with increased surgical morbidity (recommendation 40).

The data on preoperative conditioning as prehabilitation after major abdominal surgery for carcinoma are heterogeneous and inconsistent. Patients with esophageal and gastric resections appear to be particularly suitable. A meta-analysis showed that prehabilitation led to a reduced incidence of post-operative pneumonia and decreased overall morbidity (Clavien-Dindo score ≥ II). Perioperative or post-operative rehabilitation alone led to a lower pneumonia rate, shorter intensive care stay, and better health-related quality-of-life scores for dyspnea and functionality. It is clear that physical exercise and physiotherapy should be continued post-operatively.^328^

### Preoperative radiotherapy

**Recommendation 49:** Preoperative radiotherapy alone may not be recommended in operable patients with resectable esophageal cancer.

*Level of evidence: 2a. Grade of recommendation: 0*

*Consensus strength: strong consensus*

Two large meta-analyses analyzed randomized trials of preoperative radiotherapy versus surgery alone for esophageal cancer, and no survival benefit was demonstrated from preoperative radiotherapy.^328^^,^^329^ A comparative retrospective analysis of preoperative radiotherapy for esophageal cancer from the Surveillance, Epidemiology, and End Results (SEER) database^330^ has showed a significant survival advantage in patients with preoperative radiotherapy. However, the SEER database lacks data on concomitant chemotherapy, thus the potential inclusion of simultaneous chemotherapy in a large number of patients cannot be ruled out. In the opinion of the working group, the results of the studies mentioned have no influence on recommendation 49, which does not recommend preoperative radiotherapy for resectable squamous-cell carcinoma of the esophagus.

### Preoperative radiochemotherapy and perioperative chemotherapy

**Recommendation 50:** For localized adenocarcinomas of the esophagus and EGJ of category cT2, preoperative chemotherapy may be given and continued post-operatively.

*Level of evidence: 1b. Grade of recommendation: 0*

*Consensus strength: consensus*

In the available randomized trials of preoperative or perioperative chemotherapy for esophageal carcinoma, the proportion of patients with an initial T1/2 category is either not reported^331^, ^332^, ^333^, ^334^ or, as far as can be extrapolated from the primary operated patient group, very small.^335^^,^^336^ There are no separate data on the benefit of preoperative or perioperative therapy in this small subgroup of patients. Due to a lower rate of lymph node metastasis and occult distant metastases, the T2 category was prognostically more favorable than T3/4 and an expected effect of neoadjuvant therapy is probably lower. Nevertheless, patients with T2 tumors were also part of the study population in whom a survival gain could be achieved by perioperative chemotherapy.^335^^,^^337^

However, the strength of recommendation for perioperative chemotherapy is weaker (‘can’ recommendation) due to the small number of patients. Because the best evidence for the benefit of chemotherapy comes from studies with perioperative application of chemotherapy,^335^^,^^337^ post-operative continuation of chemotherapy is recommended.

Before initiating post-operative chemotherapy as part of a perioperative treatment plan, metastasis should be excluded by simple means (chest X-ray, abdominal sonography). In case of evidence of metastasis, it is not advised to continue the chemotherapy concept started preoperatively with curative intention. In published phase III trials of perioperative chemotherapy for adenocarcinoma, chemotherapy was terminated if metastases were detected.^335^^,^^337^

The following considerations should be taken into account when making decisions regarding preoperative therapy for T2 esophageal cancer.

(i) In the positive studies on preoperative therapy, T2 tumors were also included in each case and also showed a positive effect in subgroup analyses.^337^, ^338^, ^339^
(ii) Due to a staging error, it can be assumed that almost 50% of the tumors estimated preoperatively as T2N0 actually have a higher stage in the resected specimen. Markar et al.,^340^ was able to show that 34.7% in the T category and 48.1% in the N category were preoperatively attributed to a very low stage. In Speicher et al.,^341^ 41.6% were preoperatively staged too low, and in Crabtree et al.^342^ of 482 patients who were preoperatively assessed as T2N0, 27.4% were correctly staged compared with pathologic staging, 29.9% were staged lower, and 46.7% were staged higher. A more recent meta-analysis also revealed imprecise staging, with combined T-N category accuracy of only 19% and T category alone at 29%.^343^ The proportion of patients who underwent T downstaging after the histopathological result was available was 41%, while upstaging was necessary in 34%. This means that endoscopic therapy might have been sufficient for a significant proportion of patients (T1 sub-stages were not specified) and a third of patients would have benefited from neoadjuvant therapy. Because of this inaccuracy in preoperative staging there is a clinical dilemma, as patients may be deprived of chemotherapy which could have improved their cure rate.

**Figure 4:**
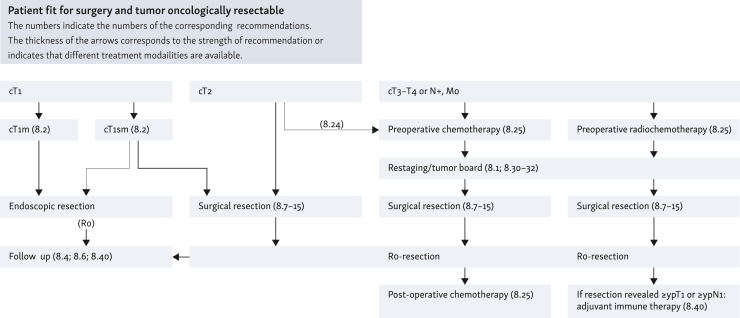
Therapy algorithm for functionally operable and oncologically resectable adenocarcinomas of the esophagus and gastroesophageal junction.

**Recommendation 51:** In operable patients with a locally advanced adenocarcinoma of the esophagus or EGJ (category cT3/T4 resectable or category cN1-3), perioperative chemotherapy or preoperative radiochemotherapy should be given (Figure 4).

*Level of evidence: 1a. Grade of recommendation: A*

*Consensus strength: consensus*

**Recommendation 52:** The use of neoadjuvant chemotherapy alone without simultaneous radiotherapy for squamous-cell carcinoma of the esophagus cannot be recommended.

*Level of evidence: 1a. Grade of recommendation: B*

*Consensus strength: strong consensus*

**Recommendation 53 (EC):** In operable patients with cT2 squamous-cell carcinoma of the esophagus, preoperative radiochemotherapy followed by complete resection can be carried out.

*Consensus strength: strong consensus*

**Recommendation 54:** In operable patients with a locally advanced squamous-cell carcinoma of the esophagus (category cT3/T4 resectable or category cN1-3), preoperative radiochemotherapy followed by complete resection should be carried out. See also recommendation 59 ‘Indication for definitive radiochemotherapy’.

*Level of evidence: 1a. Grade of recommendation: A*

*Consensus strength: consensus*

### Preoperative radiochemotherapy

From large meta-analyses, statistically significant survival advantages for combined preoperative radiochemotherapy over surgery alone were observed.^339^^,^^344^, ^345^, ^346^, ^347^, ^348^, ^349^ As stated below, many of the underlying papers are of low quality. As a result, the validity of these meta-analyses also suffers. The individual randomized trials have used cisplatin/5-fluorouracil (5-FU), plus a third agent sometimes, or carboplatin/paclitaxel simultaneously with radiotherapy at a dose of 40-50.4 Gy in conventional fractionation, corresponding to 1.8-2.0 Gy per fraction and was applied to locally advanced tumors in both squamous-cell and adenocarcinoma. However, the advantages of this treatment regimen are different in the two tumor entities, so a differentiated consideration is indicated. It often remains unclear as to which patient groups benefit.

Analogous to chemotherapy for advanced esophagogastric adenocarcinoma, cisplatin can apparently also be replaced by oxaliplatin in combined radiochemotherapy. In any case, a randomized phase II/III study including adenocarcinomas (14%) and squamous-cell carcinomas (86%) was able to prove the equivalence of FOLFOX compared with cisplatin/5-FU in the context of definitive radiochemotherapy (without surgery). Toxicity was also not different.^350^^,^^351^

### Adenocarcinoma

In locally advanced adenocarcinoma of the esophagus (category cT3/cT4 or cN+), based on four randomized phase III trials,^352^^,^^354^, ^355^, ^356^ the following parameters are relevant to the decision for or against neoadjuvant radiochemotherapy:

#### Overall survival

OS was significantly prolonged in two studies: a United States study, which was terminated prematurely due to poor recruitment (*n* = 23 versus 19) and in which squamous-cell carcinomas and all tumor stages were analyzed together.^355^ Secondly, prolonged survival was shown by the CROSS study,^352^^,^^357^ which demonstrated that the survival advantage for adenocarcinomas was lower than for squamous-cell carcinomas (median 43.2 versus 27.1 months), and a transhiatal resection was generally applied, which is no longer considered oncologically adequate for esophageal carcinoma today. Another study showed no significant advantage of neoadjuvant therapy over surgery alone^344^ (*n* = 80 versus 78), and here both chemotherapy (only one course of cisplatin/5-FU) and radiotherapy (35 Gy) were below the doses usually used. Whether stage cT2 adenocarcinomas also benefit from neoadjuvant radiochemotherapy remains controversial. While stage cT2 tumors were included in the CROSS study, the FFCD 9901 trial did not observe a survival benefit for UICC stage IIa tumors.^358^

#### Recurrence-free survival

In two studies, recurrence-free survival was prolonged by trimodal therapy.^359^, ^360^, ^361^ Recurrence-free survival was better in the discontinued study after trimodal therapy for the mixed collective of squamous-cell and adenocarcinoma at 1.01 versus 3.47 years.^359^ Recurrence-free survival was also prolonged after trimodal therapy in the CROSS study (median 17.7 versus 29.9 months).^352^^,^^357^ One study showed no benefit in progression-free survival by trimodal therapy for adenocarcinomas.^354^

#### R0 resection rate

R0 resection rate was improved in three studies, from 59% to 80% (squamous-cell and adenocarcinomas),^354^ and from 69% to 92% (squamous-cell and adenocarcinomas),^352^^,^^357^ respectively. In the third study, which examined only adenocarcinomas of the GE junction, the R0 resection rate was improved from 80% to 100%.^356^

### Squamous-cell carcinoma

#### Overall survival

Two studies did not detect significant survival benefit from trimodal therapy.^354^^,^^362^ One combined all resectable tumors (category cT1-3 and cN0-1).^354^ The other study, which included tumors of category >cT1 and/or >cN0, was terminated prematurely due to an unexpectedly high rate of conversion to surgery (31%).^362^ Conversely, two studies demonstrated a marked increase in OS following trimodal therapy: the median follow-up for survival was 21.1 versus 81.6 months (*n* = 41 versus 43) for tumors in the category of >cT1 and/or >cN0,^352^^,^^357^ and 66.5 versus 100.1 months (*n* = 227 versus 224) for cN+ or cT4 N0 tumors.^363^ One study showed a trend toward better OS with chemoradiotherapy in long-term follow-up (41.5 versus 56.5 months) (*n* = 64 versus 76).^364^ This benefit was independent of whether chemoradiotherapy was given before or after resection, although only 40% of patients were able to receive post-operative therapy according to protocol.

#### Recurrence-free survival

In only one study was recurrence-free survival not prolonged by trimodal therapy (at 2 years 51% versus 49%).^362^ In the other studies, recurrence-free survival in squamous-cell carcinoma was significantly prolonged by neoadjuvant radiochemotherapy (11.6 versus 74.7 months^352^^,^^357^; 39.5 versus 48 months^364^; 41.7 versus 100.1 months).^363^

#### R0 resection rate

Trimodal therapy was also reported to improve the R0 resection rate compared with surgery group: the difference was 87.5% versus 100%^362^; 69% versus 92%^352^; 91.2% versus 98.4%,^363^ respectively. It should be mentioned here again that the studies differed significantly in therapeutic technique and inclusion criteria as described above.

Overall, a positive effect of preoperative chemoradiotherapy can be demonstrated, especially in squamous-cell carcinoma. However, the question of which subgroups of patients benefit from this in clinical reality remains unresolved. This uncertainty arises in particular from the lack of accuracy of preoperative staging, especially with regard to tumor-involved lymph nodes. For example, a study in early squamous-cell carcinoma (stages I-IIb) showed that of the patients in the arm with primary surgery, 39% actually had stage III, i.e. were ‘understaged’.^358^ Surgical technique and radicality also play a role and are not standardized. In addition, the experience of a center must be considered (hospital volume) because it influences post-operative lethality and long-term outcomes. It is conceivable that tumors in the cT3N0 category could be treated equally well with primary surgery, i.e. patients would be overtreated by the standard of trimodal therapy. However, we know from older studies that tumors of category cT3 develop at least regional lymph node metastases in >80% of cases,^365^ and patients in this case have a very poor prognosis even after optimal surgery.^249^

Few studies precisely differentiate the included stages and only one study investigates tumor stages I and II. The study by Mariette et al.^358^ investigated T1-2, N0-1 and T3N0, M0, but included both adenocarcinomas and predominantly squamous-cell carcinomas. There was no survival benefit shown by neoadjuvant therapy. An analysis of T3 squamous-cell carcinomas was previously published from the same group. Here, enhanced survival benefit was shown by neoadjuvant radiochemotherapy in tumors of the category T3N0.^366^ The center-specific, often low reliability of preoperative staging with respect to lymph node involvement, the clearly pronounced variability in the radicality of surgery, and the associated different R0 resection rates make it difficult in this situation to draw up generally valid recommendations for this particular tumor situation (clinical stage T3 N0 M0).

Montagnani et al.^346^ reviewed all forms of multimodality therapy for squamous-cell carcinoma of the esophagus and found a significant advantage over surgery alone for preoperative chemoradiotherapy followed by surgery and for definitive chemoradiotherapy. In the rank probability analysis, preoperative chemoradiotherapy had the highest probability of improving prognosis over surgery alone. The data of this comprehensive analysis confirm the strategy of preoperative radiochemotherapy plus surgery as a standard recommendation for (locally advanced) squamous-cell carcinoma of the esophagus.

**Recommendation 55:** SEMS ought not to be used due to an increased complication rate with planned neoadjuvant radiochemotherapy or as a bridge to surgery.

*Level of evidence: 4. Grade of recommendation: B*

*Consensus strength: Strong Consensus*

Preoperative SEMS insertion as a bridge to surgery or before planned perioperative radiochemotherapy is not recommended because it is associated with an increased incidence of complications and valid alternatives exist with the insertion of feeding tubes. A systematic meta-analysis of nine studies involving 180 patients showed that there was a high rate of severe complications (stent migration in 32% and pain in 51.4%).^367^ A European cohort study of 2944 patients yielded similar negative results.^368^ Preoperative mortality was 13.2% in the stent group versus 8.6% in the control group and morbidity was 63.2% versus 59.2%. The R0 resection rate, median time to recurrence, and 3-year survival were also significantly worse in the SEMS group than in the no-stent group. Accordingly, the ESGE in its 2016 published recommendations^369^ does not recommend stent implantation in concurrent radiotherapy as well as a bridge to surgery or before preoperative radiochemotherapy.

Recent systematic reviews^370^^,^^371^ also show that implantation of a stent can worsen oncological outcomes. In a retrospective observational study from Finland and Sweden,^372^ preoperative implantation of a stent increased 30-day lethality (3.9% versus 1.6%) and 90-day lethality (11.8 versus 7.0%) without these differences reaching statistical significance. A retrospective study from the Netherlands^373^ shows that radiochemotherapy carried out earlier leads to increased stent complications.

### Restaging after preoperative multimodal therapy

**Recommendation 56 (EC):** After completion of a preoperative therapy, a new exclusion of distant metastases should be carried out. Restaging of the local findings can be carried out for planning the surgery.

Consensus strength: strong consensus

**Recommendation 57 (EC):** If clinical signs of tumor progression occur during preoperative therapy, symptom-based diagnostics should be carried out. If endoscopic or imaging evidence of local tumor progression is present, surgery should be carried out early.

*Consensus strength: strong consensus*

In the phase III trials of perioperative therapy, neoadjuvant therapy was carried out as planned in the absence of evidence of tumor progression and led in this form to an improvement in survival for the entire patient group.^335^^,^^337^^,^^374^

However, if during the course of neoadjuvant therapy there are clinical signs of tumor progression (worsening of tumor-related symptoms or general condition), it seems reasonable to carry out a symptom-oriented diagnosis with a renewed CT and endoscopy. In case of local tumor progression during neoadjuvant therapy, early surgery should be carried out, as patients are unlikely to benefit from continuing this therapy. To date, there are no sufficient data to justify a change in therapy or intensification of therapy. However, preoperative therapy should not be discontinued if there is no tumor progression.^246^

### Response prediction

**Recommendation 58 (EC):** The clinical utility of FDG–PET for response assessment of chemotherapy or radiochemotherapy before surgery is controversial, which is why FDG–PET/CT should not be routinely carried out in this setting.

*Consensus strength: strong consensus*

The value of FDG–PET in predicting response to neoadjuvant chemotherapy has been investigated by several research groups.

The sequential investigation of tumor glucose uptake during neoadjuvant chemotherapy or radiochemotherapy was examined using FDG–PET. It was observed that a decrease in the standard uptake value (SUV) by ≥35% in relation to baseline within 2 weeks after starting neoadjuvant chemotherapy has a high accuracy for predicting histopathological response after neoadjuvant chemotherapy.^375^, ^376^, ^377^ In particular, the correct detection of non-responders (negative predictive value) was remarkably high,^375^ in up to 95% of patients studied. Metabolic response during neoadjuvant chemotherapy was further found to be associated with overall prognosis in patients with adenocarcinomas of the esophagus and EGJ. Early metabolic response (PET response) was found to be more accurate in predicting histologic regression than morphologic response using high-resolution multislice CT.^378^ Based on these observations, the MUNICON trial was able to demonstrate for the first time that early PET response can be the basis of an individualized treatment plan in adenocarcinoma of the esophagus and EGJ.^379^^,^^380^ However, it has not yet been convincingly demonstrated that early PET-based treatment algorithms can improve the overall prognosis of patients with esophageal cancer, or avoid ineffective chemotherapy.

The CALGB phase II trial investigated whether the metabolic response to chemotherapy in early PET–CT can guide preoperative therapy.^381^ Patients with esophageal and EGJ adenocarcinoma received either FOLFOX or carboplatin/paclitaxel (CP) as initial chemotherapy. If metabolic response was observed (≥35% decrease in SUV), the same chemotherapy continued during preoperative chemoradiotherapy. Otherwise, chemotherapy was switched to an alternative during chemoradiotherapy. Patients with PET response after FOLFOX and subsequent chemoradiotherapy with FOLFOX had the highest rate of pathological complete response (pCR) at 40.3%. This was significantly higher than with PET response after CP and subsequent chemoradiotherapy with CP (14.1%). Switching chemotherapy to preoperative radiotherapy after a lack of metabolic response led to similarly low pCR rates as with the continuous administration of CP (FOLFOX→CP 18.0%, CP→FOLFOX 20.0%), but could not compensate for the non-response to induction chemotherapy. The survival rate after 5 years was also significantly higher for FOLFOX responders (53.0%) than for CP responders (43.9%) or for non-responders after FOLFOX or CP and switching from chemotherapy to radiotherapy (37.5% versus 40.4%). The study confirms the role of early PET–CT as a predictor of histologic response but not as a tool for improving prognosis in non-responders.

Furthermore, the predictive power of early PET-based response during neoadjuvant chemotherapy cannot be transferred to concurrent radiochemotherapy without trade-offs. The positive and negative predictive power with regard to histopathological regression proved to be significantly weaker in several studies.^382^, ^383^, ^384^, ^385^ This is attributed, among other things, to radiotherapy-induced inflammatory responses, which can lead to signal alterations in FDG–PET that are difficult to interpret.^386^

### Indication for definitive radiochemotherapy

**Recommendation 59:** Definitive radiochemotherapy should be given irrespective of the histological entity of the esophageal cancer if the tumor is deemed surgically/endoscopically unresectable at an interdisciplinary tumor conference or if a patient is functionally inoperable, or declines surgery after detailed explanation.

*Level of evidence: 1b. Grade of recommendation: A*

*Consensus strength: strong consensus*

For patients who are medically inoperable or whose esophageal cancer is assessed as unresectable, there is a curative chance with definitive radiochemotherapy, provided there are no distant metastases. Long-term survival rates of 10%-35% at 5 years have been observed in prospective studies^387^^,^^388^ and in large registry studies,^387^^,^^388^ for both stage II-III squamous-cell carcinoma and adenocarcinoma. Therefore, long-term survival depends on the T- and N category after endoscopic ultrasound and CT, as well as the general condition of the patients.^388^, ^389^, ^390^, ^391^

Definitive radiochemotherapy is more effective than radiotherapy alone, so the combination should always be preferred in patients without contraindications to cisplatin-, carboplatin-, or oxaliplatin-containing chemotherapy.^353^^,^^392^ For treatment regimens, please refer to Table 6.

**Table 6:** Possible chemotherapy regimens for neoadjuvant preoperative radiochemotherapy

Substances	Dosage	Application	Days
**1. 5-fluorouracil (5-FU)/cisplatin**			
5-FU	1000 mg/m^2^	24 h infusion	d 1-4, 29-32
Cisplatin	75 mg/m^2^	i.v. (60 min)	d 1, 29
**2. Carboplatin/paclitaxel**			
Carboplatin	AUC 2	i.v. (60 min)	d 1, 8, 15, 22, 29
Paclitaxel	50 mg/m^2^	i.v. (60 min)	d 1, 8, 15, 22, 29
**3 FOLFOX**			
Oxaliplatin Folinic acid 5-FU 5-FU	85 mg/m^2^ 200 mg/m^2^ 400 mg/m^2^ 1600 mg/m^2^	i.v. 2 h i.v. 2 h i.v. 10 min i.v. 46 h	Day 1 Day 1 Day 1 Day 1-2 Repeat every 2 weeks, 3 cycles during neoadjuvant radiochemotherapy

5-FU, 5-fluorouracil; AUC, area under the curve; i.v., intravenous.

Source:^350^^,^^352^^,^^353^

In clinical stage I, long-term outcomes after definitive radiochemotherapy are 60%-70% better for T1 tumors with 5-year survival rates compared to higher stages.^391^^,^^393^ There are also sufficient data on local control and long-term survival after percutaneous radiotherapy ± brachytherapy alone for T1 N0 M0 tumors. In these tumors, local tumor control rates of 45%-90% and 5-year survival rates of 38%-76% have been achieved in several studies, so if there are contraindications to concomitant chemotherapy, definitive radiotherapy can be used in inoperable patients.^394^^,^^395^

It is currently unclear whether patients with clinically complete remission (varying definitions in the literature) after curatively intended chemoradiotherapy benefit from surgical resection.^396^ A meta-analysis summarized four retrospective studies (648 patients) that followed predominantly patients with squamous-cell carcinoma.^397^ Patients with surgical resection did have significantly better DFS at 2 years. However, at 5 years, neither DFS (HR 1.78, 95% CI 0.87-3.66) nor OS (HR 1.36, 95% CI 0.57-3.24) were significantly different. Clinical trials on this topic are currently active in France (squamous-cell carcinoma only) and the Netherlands. The problem lies, among others, in the prediction of histologically complete destruction of the tumor by clinical methods including PET–CT, MRI,^84^ and biopsies in the former tumor region. However, the aforementioned data of retrospective studies allow a cautious approach (watch and wait) after clinically complete remission in case of patient request for organ preservation or increased surgical risk. Regular follow-up with endoscopy and CT is useful if evidence of localized tumor progression in the esophagus may lead to a prompt surgical resection (so-called salvage surgery). Salvage surgery opens up the chance of long-term survival, especially if a clinically complete remission was diagnosed primarily after chemoradiotherapy.^398^

**Figure 5:**
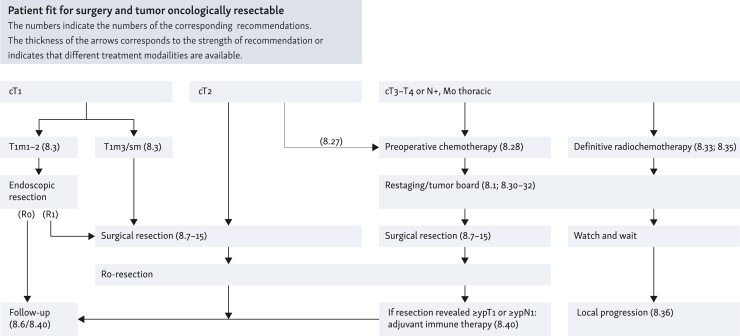
**Therapy algorithm for functionally operable and oncologically resectable squamous-cell carcinoma of the esophagus.** For therapy using definitive radiochemotherapy for localized squamous-cell carcinoma of the cervical esophagus, see Recommendation 60.

**Recommendation 60:** In patients with localized squamous-cell carcinoma of the cervical esophagus, definitive radiochemotherapy should be preferred over primary surgical resection (Figure 5).

*Consensus strength: strong consensus*

Long-term survival rates of 17%-55% are achieved with definitive radiochemotherapy in squamous-cell carcinoma of the cervical esophagus.^398^^,^^399^ The best results were observed in series with a high proportion of stage I and IIA patients.^400^ Simultaneous chemotherapy of definitive radiochemotherapy was carried out in squamous-cell carcinoma of the cervical esophagus as in carcinoma of the thoracic esophagus. The total radiation dose in the larger retrospective case series was mostly above 50 Gy at 60-66 Gy in conventional fractionation,^398^^,^^399^ and thus higher than in patients with thoracic esophageal cancers. Regarding target volumes, the macroscopic primary tumor was included in the clinical target volume with craniocaudal safety margins of 3.0-4.0 cm and radial safety margins of 1.0-1.5 cm, but not beyond intact anatomical borders, up to a base dose of 50 Gy. Macroscopically affected lymph nodes were included in the clinical target volume with a margin of 1.5 cm.^395^^,^^401^^,^^402^ To account for setup deviations in the daily setting, a margin of 1.0-1.5 cm in all spatial directions was added around the clinical target volume (CTV). The resulting planning target volume then included the macroscopic tumor in the rule with a margin of 4-5 cm craniocaudally and 1.5-2.0 cm radially. For carcinomas of the cervical esophagus, lymph node stations with a high risk of involvement, the cervical parajugular lymph nodes of level III-IV, the paratracheal and paraesophageal lymph nodes of the neck and in the upper mediastinum, and the medial supraclavicular lymph nodes were usually also included electively in the target volume up to a base dose of 50 Gy.^403^^,^^404^ However, the evidence for elective co-irradiation of clinically unaffected lymph node stations is low.^404^ Above 50 Gy, the clinical target volumes are reduced to ∼1.0 cm around the macroscopic tumor.^405^

Comparative studies between surgical approach with neoadjuvant radiochemotherapy and definitive radiochemotherapy exist only in squamous-cell carcinoma of the thoracic esophagus. They showed no difference in survival there.^225^ In cervical esophagus, the morbidity of surgery with and without pharyngolaryngectomy is higher than in thoracic esophagus.^399^^,^^406^^,^^407^ Therefore, surgery should be carried out only in specialized centers. The 5-year survival rates after surgery with or without neoadjuvant or adjuvant radiochemotherapy are 14%-47% in the larger series, a range also covered by definitive radiochemotherapy studies.^399^

**Recommendation 61:** In patients with resectable squamous-cell carcinoma of the intrathoracic esophagus of category cT3/cT4, definitive radiochemotherapy ought to be carried out as an alternative to surgical resection. See also recommendation 54.

*Level of evidence: 1a. Grade of recommendation: B*

*Consensus strength: consensus*

Randomized trials comparing definitive radiochemotherapy with surgery were conducted in patients with resectable squamous-cell carcinoma of the thoracic esophagus category cT3/cT4 without distant hematogenous metastases. In the majority of studies, surgery was preceded by neoadjuvant radiochemotherapy in the surgical arms.^225^^,^^347^ None of the studies showed a significant survival benefit in the surgery arm. Also, the meta-analyses showed no differences in survival.^225^^,^^347^ Locoregional recurrences, on the other hand, were more frequent after definitive radiochemotherapy than after an additional surgery. Furthermore, the need for bougienage was more frequent. After neoadjuvant radiochemotherapy and surgery, distant metastases were the predominant location of recurrence.^408^ Treatment-related lethality was higher in the patient groups with surgery than after definitive radiochemotherapy without surgery. Thus, there are differences in locoregional efficacy and in the incidence of severe side-effects, which are important for recommending therapy in individual patients with similar survival.

Two meta-analyses compared definitive and preoperative chemoradiation therapy. One meta-analysis indicated that trimodal therapy conferred an advantage in local recurrence rate (HR 0.35, CI 0.22-0.57) and OS (HR 0.65, CI 0.56-0.76).^409^ Another review compared individual therapy strategies and carried out rank probability analysis.^346^ A significant advantage over surgery alone was found for preoperative chemoradiotherapy followed by surgery and for definitive chemoradiotherapy. The highest risk reduction of 38% was achieved by definitive chemoradiotherapy: HR 0.62, CI 0.41-0.96. In the rank probability analysis, definitive radiochemotherapy, followed by neoadjuvant radiochemotherapy and surgery, had the highest probability of improving prognosis compared with surgery alone (82.8% versus 54.9%). Neoadjuvant radiochemotherapy had the most robust results concerning survival benefits. Hence, the trimodal therapy seems to have the greatest advantage as compared with surgery alone.

The data confirm the strategy of preoperative radiochemotherapy plus surgery as a standard recommendation for (locally advanced) squamous-cell carcinoma of the esophagus. Furthermore, definitive radiochemotherapy is a well-documented treatment alternative (especially for high-grade tumor, questionable resectability of the tumor, increased risk of surgery for the patient, patient age >70 years, desire for organ preservation).

In definitive radiochemotherapy, chemotherapy regimens simultaneous to radiotherapy including cisplatin and a fluoropyrimidine,^408^^,^^410^^,^^411^ carboplatin and paclitaxel,^352^^,^^412^ cisplatin and paclitaxel,^413^ or the FOLFOX4 regimen^413^ are effective and tolerable. Regarding the total dose of radiotherapy to be applied for definitive radiochemotherapy, the North American Intergroup Study INT0123 showed no survival benefit after the higher total radiation dose of 64.8 Gy compared to the lower total dose of 50.4 Gy with conventional fractionation.^410^ However, this one study may be criticized as indicating an imbalance of prognostic factors because of clustered protocol violations and higher mortality in the high-dose arm during a period when therapy is the same in both arms. Since this study, a total radiation dose of 50.4 Gy with concurrent chemotherapy has been the standard dose for definitive radiochemotherapy in North America.^401^ However, the randomized trials comparing neoadjuvant radiochemotherapy and surgery with definitive radiochemotherapy for squamous-cell carcinoma of the esophagus all used total radiation doses of ≥60 Gy.^236^^,^^408^^,^^414^ These studies show that higher total doses are safe to apply during definitive radiochemotherapy. Retrospective analyses indicate a dose–effect relationship for local tumor control using definitive radiochemotherapy.^390^

In the ARTDECO study,^415^ 50.4 Gy [standard dose (SD); 1.8 Gy/fraction] was prospectively randomized compared with 61.6 Gy (HD; 1.8 Gy/fraction plus boost to tumor at 0.4 Gy/fraction) in combination with weekly carboplatin and paclitaxel (*n* = 260 pat.). The primary study endpoint was locoregional progression-free survival (LPFS); secondary study endpoints were locoregional progression-free survival (LRPFS), OS, and toxicity. Patients with squamous-cell carcinoma (61.9%) and adenocarcinoma (38.2%) of the esophagus and EGJ were included in the study based on medical inoperability, technical irresectability, or patient preference (T1-4N0-3M0 or M1 in supraclavicular lymph nodes). Staging also included PET–CT. After a median follow-up of 50 months, oncologic outcomes were as follows.

From the results it can be concluded that an increase in the radiation dose from 50.4 to 61.6 Gy does not lead to an improvement in local control or survival, so a dose of 50.4 Gy is conclusively considered the standard dose by the authors (Table 7).

**Table 7:** Oncological outcomes in the ARTDECO definitive radiochemotherapy trial comparing the 50.4 Gy standard dose with 61.6 Gy in combination with carboplatin and paclitaxel

	SD 50.4 Gy			HD 61.6 Gy			*P* value
	Total	SCC	AC	Total	SCC	AC	
3-year LPFS	71%			73%			0.62
		75%	61%		79%	61%	0.59
3-year LRPFS	53%			59%			0.24
		58%	42%		64%	49%	0.26
3-year PFS	33,1%			25.4%			0.31
3-year OS	42%			39%			0.22
Grade 4/5 toxicity	13%/3%			14%/8%			NS

AC, adenocarcinoma; HD, high dose; LPFS, local progression-free survival; LRPFS, locoregional progression-free survival; OS, overall survival; NS, not significant; PFS, progression-free survival; SCC, squamous-cell carcinoma; SD, standard dose.

Source: ^415^

In terms of target volumes, the macroscopically affected primary tumor with craniocaudal safety margins of 3.0-4.0 cm and radial safety margins of 1.0-1.5 cm, but not beyond intact anatomic borders, is included in the clinical target volume up to a base dose of 50 Gy. Macroscopically affected lymph nodes are included in the clinical target volume with a margin 0.5-1.5 cm.^395^^,^^401^^,^^402^ Above 50 Gy, clinical target volumes are reduced to ∼1.0 cm around the macroscopic tumor. Dose-volume limits for the lung are chosen lower in some institutions for radiotherapy of esophageal carcinoma than for lung carcinoma.^416^^,^^417^

Intensity-modulated radiotherapy can reduce the burden on the heart compared with three-dimensional conformal radiotherapy.^416^ There is no higher-grade evidence for elective irradiation of clinically unaffected lymph node stations and therefore its necessity is controversial.

### Procedure for tumor persistence/local recurrence after radiochemotherapy

**Recommendation 62 (EC):** In case of tumor persistence or local recurrence without distant metastases after radiochemotherapy, salvage surgery can be attempted with curative intent. Careful evaluation of operability and resectability should be carried out by a treatment team experienced in esophageal surgery.

*Consensus strength: strong consensus*

For a long time, it was considered an unwritten law that esophageal resection should not be carried out later than 6 weeks after completion of radio(chemo)therapy because the onset of fibrosis would not only make surgery technically more complex but also significantly increase post-operative lethality.

Retrospective data from large centers in Italy and the United States have shown, however, that relevant post-operative complications and lethality do not increase significantly when surgery is carried out later than 8 weeks after therapy.^418^^,^^419^ This is true for squamous-cell and adenocarcinomas, provided there is considerable experience of the treatment team.

These findings are the basis for pursuing the concept of salvage surgery (surgery for selected patients only) and contrasting it with the results of planned surgery (surgery as a rule). In salvage surgery, two different scenarios must be distinguished: esophagectomy immediately following ineffective radiochemotherapy (histologically proven tumor persistence) and surgery for histologically proven, isolated local recurrence after primary clinically complete remission by radiochemotherapy (tumor recurrence).

Numerous groups, especially from Asia, have published retrospective data of their mostly unicentric experiences.^420^ From these publications it can be seen that post-operative complications and prognosis differ significantly depending on whether patients with tumor persistence or tumor recurrence after CR are involved. Furthermore, R0 resection is the most important prognostic factor,^421^ and salvage surgery is only useful if complete resection is successful. Hospital mortality ranges from 6% to 22%.

A more recent literature search from 2007 to 2017 selected 28 studies in which 1046 patients with persistent or recurrent esophageal cancer underwent salvage esophageal resection after definitive radiochemotherapy.^422^ Patients with persistent esophageal cancer had a significantly higher post-operative complication rate: respiratory (36.6% versus 22.7%) and cardiovascular (10.4% versus 4.5%). Pooled 30- and 90-day mortality rates were 2.6% and 8%, respectively. The 3-year and 5-year OS were 39% and 19.4%, respectively. Statistically, OS did not differ significantly whether persistent or recurrent tumor had been present. The authors concluded that salvage surgery would be a potentially curative treatment option for patients in whom surgery was not initially carried out but who are in an operable state.^422^

Marks et al. demonstrated the outcomes of salvage surgery after definitive chemoradiation (CRT) in patients with adenocarcinoma of the esophagus^423^: 65 patients who received trimodal therapy (chemoradiotherapy + 50 Gy followed by surgery) were compared with 65 patients who had similar risk factors but received surgery after the same chemoradiotherapy (definitive CRT with 50 Gy) only if local tumor persistence or local tumor recurrence occurred. The median interval between the end of CRT and salvage surgery was 31 weeks. The rate of R0 resections was higher after planned surgery (99% versus 91%). Complication rates (e.g. anastomotic insufficiency 16.9% versus 18.5%), hospital mortality (7.7% versus 4.6%), and survival at 3 years (55% versus 48%) did not differ significantly.

A prospective study from Japan^424^ treated patients with localized squamous-cell carcinoma of the esophagus on the basis of so-called informed decision. Patients chose either chemoradiotherapy (cisplatin/5-FU + 60 Gy) followed by surgery (*n* = 48) or the same chemoradiotherapy followed by salvage surgery only for isolated local tumor remnant/tumor progression (*n* = 51). From the first group 46/48 patients received surgery, and from the second group only 13/51 patients (26%) received surgery. Median survival time (41.2 versus 52.9 months) and survival rate at 5 years (51% versus 76%) were significantly higher in the group with salvage surgery.

A meta-analysis comparing planned versus selective surgery after chemoradiotherapy for esophageal cancer found that salvage surgery was associated with significantly more complications (e.g. anastomotic insufficiency) and higher post-operative mortality, although the rate of incomplete resections was not different.^226^ A registry analysis by the French FREGAT group, involving 2944 patients with esophageal cancer, highlighted that 308 patients who underwent salvage surgery had an 8.4% hospital mortality and 34.7% morbidity rate.^425^ Squamous-cell carcinoma and radiation dosages ≥55 Gy were independent predictors of higher morbidity. Anastomotic insufficiency occurred in 12.7% of cases, notably more frequent with cervical anastomosis. Thirty-four percent of patients were alive after 5 years; a radiation dose ≥55 Gy, the occurrence of post-operative complications, ypTNM stage III, and R1 resections correlated significantly with poorer long-term survival. It is important to note that the radiation dose in the setting of definitive radiochemotherapy should not exceed 54 Gy in order to keep the complication rate of salvage surgery within acceptable limits.

In summary, the concept of selective salvage surgery after chemoradiotherapy is feasible. The procedure is particularly suitable for patients with increased surgical risk and/or high tumor site (above the bifurcatio tracheae). The equivalence with a trimodal therapy with regard to prognosis has not yet been proven.

### Importance of targeted therapies for preoperative concepts

**Recommendation 63 (EC):** Antibodies and small molecules shall not be used in preoperative therapy.

*Consensus strength: consensus*

In the palliative treatment of advanced adenocarcinomas of the stomach or of the gastroesophagogastric junction, phase III data have been published demonstrating a significant survival benefit for patients with HER2-positive tumors when they received the HER2 antibody trastuzumab,^426^ in addition to chemotherapy consisting of cisplatin and a fluoropyrimidine. However, despite first results from (randomized) phase II studies, it is still unclear whether trastuzumab in combination with chemotherapy improves the results of preoperative therapy in localized HER2-positive carcinomas.^246^ Trastuzumab should therefore not be used for localized tumors.

Even in preoperative chemoradiation therapy for HER2-positive adenocarcinoma, the addition of trastuzumab to chemotherapy with carboplatin and paclitaxel did not improve survival times (DFS 19.6 versus 14.2 months, HR 0.99, *P* = 0.97). The use of trastuzumab is not indicated in this situation.

There are no positive results of targeted therapy or therapy with monoclonal antibodies in squamous-cell carcinomas of the esophagus. The use of targeted agents is therefore not indicated outside of clinical trials.

### Value of post-operative adjuvant radiotherapy or radiochemotherapy

**Recommendation 64:** After R0 resection of squamous-cell carcinoma, adjuvant radiotherapy or radiochemotherapy ought not to be carried out.

*Level of evidence: 1a (radiotherapy); 4 (radiochemotherapy). Grade of recommendation: B*

*Consensus strength: consensus*

Recommendation 64 applies to a small group of patients who, on the one hand, were staged too low preoperatively and, on the other hand, are capable of receiving further therapy post-operatively. For those patients who already have locally advanced squamous-cell carcinoma at initial staging (cT3-4 or cN1-3), this recommendation explicitly does not apply (see instead recommendation 54).

According to the American Society of Clinical Oncology (ASCO) expert guideline, patients with locally advanced carcinoma of the esophagus should be offered multimodal therapy.^427^ This also applies to patients with locally advanced squamous-cell carcinoma of the esophagus. Risk factors for poor outcomes include a high number of affected^428^ or a low number of removed lymph nodes,^239^ as well as an unfavorable ratio (>20%) from the quotient of the number of affected lymph nodes to the number of histopathologically examined lymph nodes.^429^^,^^430^ Randomized studies on the effectiveness of ‘adjuvant (post-operative) radiotherapy’ alone for squamous-cell carcinoma of the esophagus after complete resection were summarized by Malthaner et al.^431^ Post-operative radiotherapy was not associated with any significant survival benefit, and there was no trend toward better survival with post-operative radiotherapy. Thallinger’s study of 45 randomized patients^432^ compared post-operative radiotherapy with 50 Gy in conventional fractionation and simultaneous cisplatin/5-FU with post-operative chemotherapy alone. No trend toward a survival benefit with post-operative radiochemotherapy was found. In a more recent meta-analysis, Liu et al. included a total of 8198 patients from 6 RCTs and 13 retrospective studies of adjuvant radiotherapy for squamous-cell carcinoma of the esophagus (surgery 5419, surgery + radiotherapy 2779).^433^ While only the retrospective studies (with a high risk of bias) show an advantage in OS, all RCTs evaluable for this purpose (414 patients) only show a significant advantage for DFS (HR 0.69, CI 0.54-0.88). The weakness of the meta-analysis is the low quality of the included studies and the fact that it is unclear how many patients also received adjuvant chemotherapy in addition to radiotherapy.

For ‘post-operative radiation chemotherapy’, Lv et al. carried out one randomized study in the unicenter study from China,^364^ which was included also in the systematic ASCO guideline.^427^ A total of 238 patients with squamous-cell carcinoma of the esophagus were randomized into three arms: surgery alone (*n* = 80), preoperative radiochemotherapy (*n* = 80), and post-operative radiochemotherapy (*n* = 78). Progression-free survival (PFS) was significantly better with both preoperative and in the arm with post-operative radiochemotherapy than after surgery alone. However, the follow-up methods (telephone or outpatient service, i.e.) raise concerns and render the primary endpoint vulnerable. Improved OS was also demonstrated in multimodality therapy compared to surgery alone, although 20% of patients in the arm with post-operative radiochemotherapy had received no resection or only incomplete resection. A network meta-analysis of 25 prospectively randomized and retrospectively comparative studies^346^ also found a trend for improved prognosis with post-operative radiochemotherapy compared with surgery alone, but with wide confidence intervals. Other meta-analyses have shown inconsistent findings, with some showing no benefit and others suggesting advantages for post-operative chemoradiotherapy in terms of OS and recurrence probability.^434^^,^^435^

Overall, the evidence for post-operative radiochemotherapy must be considered weak. However, given the unfavorable prognosis after surgery alone of patients with thoracic squamous-cell carcinoma in post-operative stage III with the stated risk factors, the indication for post-operative radiochemotherapy can be made in individual cases with an increased risk of local recurrence. This can be carried out, for example, following the scheme of the CROSS study,^352^^,^^357^ up to a total dose of 50.4 Gy. Careful minimization of toxicity by selecting individualized target volumes to high-risk regions should be considered. Affected lymph node stations should be included in the target volume, although the anastomotic region and primary tumor bed need not be obligatorily included if no affected lymph nodes have been detected in the immediate vicinity.^436^

Regarding toxicities from post-operative radiotherapy, small randomized trials from the 1980s showed more pronounced toxicities. These studies were carried out either with high single doses of 3.5 Gy per fraction,^318^ which have been abandoned today in favor of fractionation with 1.8 Gy per fraction, or with very large target volumes.^319^ Such toxicities have not been seen, or have been seen to a much lesser extent, in more recent studies of post-operative radiotherapy with current radiotherapeutic technology.^437^^,^^438^

Because of the higher effectiveness of radiochemotherapy compared with radiotherapy alone, which has been demonstrated particularly in preoperative therapy, it appears reasonable to combine radiotherapy with simultaneous chemotherapy if the indication for post-operative therapy is made in individual cases. This recommendation is supported by a small randomized study from China.^439^ There, adjuvant radiotherapy or radiochemotherapy significantly improved DFS in pathologic stage IIb and III squamous-cell carcinoma compared to observation (DFS after 3 years 53.8% versus 36.7%). The benefit was greater for 64 patients with adjuvant radiochemotherapy compared with 54 patients with adjuvant radiotherapy. However, a comparison of the adjuvant treatment strategies is only formally exploratory due to the small comparison groups.

**Recommendation 65:** After primary R0 resection of a non-pretreated adenocarcinoma in the EGJ, adjuvant radiochemotherapy or chemotherapy may be carried out if there is an increased risk of recurrence (pN1-3).

*Level of evidence: 1b. Grade of recommendation: 0*

*Consensus strength: strong consensus*

The evidence supporting benefit from preoperative radiochemotherapy or perioperative chemotherapy is higher than that for post-operative radiochemotherapy, so preoperative treatment should be clearly preferred. If no preoperative therapy was skipped because of preoperatively underestimated tumor expansion (‘understaging’), post-operative radiochemotherapy or post-operative chemotherapy may be carried out. However, post-operative therapy alone is not an equivalent alternative to preoperative therapy, so neoadjuvant therapy ought to be used whenever possible and indicated.

Before starting adjuvant radiochemotherapy or chemotherapy, the continuation of the curative intention to treat must be verified and distant metastases must be excluded. In case of distant metastases, a strategy of combined radiochemotherapy is no longer useful. Instead, conversion to palliative therapy is indicated.

A meta-analysis of eight randomized trials found that post-operative radiochemotherapy, compared with surgery alone, led to enhanced progression-free survival (HR 0.66, 95% CI 0.55-0.78) and OS (HR 0.75, 95% CI 0.63-0.89).^439^ It also showed improved progression-free survival (HR 0.77, 95% CI 0.65-0.91) compared with surgery with post-operative chemotherapy. The proportion of carcinomas of the EGJ and cardia in the studies was 6%-23%.^359^^,^^440^, ^441^, ^442^ Therefore, as with gastric cancer, post-operative radiochemotherapy may be carried out for adenocarcinomas of the EGJ if an increased local recurrence risk is assumed. This may be the case with multiple affected lymph nodes or limited lymphadenectomy.^359^^,^^441^ Adjuvant post-operative radiochemotherapy involves a total dose of 45-50.4 Gy applied in conventional fractionation with 1.8-2.0 Gy per fraction, five fractions per week. Concerning the original scheme in the intergroup 0116 (INT-0116) study, the National Comprehensive Cancer Network guideline for esophageal cancer voiced concerns regarding safety issues. Simultaneous chemotherapy, exemplified by capecitabine as in the ARTIST1 study, may be more appropriate.^359^ This is even more so since the phase III Alliance trial^443^ showed no benefit when intensified therapy with epirubicin/cisplatin/5-FU (ECF) was used instead of chemotherapy with 5-FU/folinic acid before and after adjuvant chemoradiotherapy.

In the ARTIST 1 trial, the benefit of radiochemotherapy appeared to be particularly significant in lymph node-positive patients.^359^ In the phase III Artist 2 trial, therefore, only patients with gastric cancer and positive lymph nodes were included and treated post-operatively with chemotherapy or radiochemotherapy alone.^444^ The addition of radiotherapy to effective chemotherapy with S1 and oxaliplatin did not improve the rate of recurrence and DFS compared with chemotherapy with S1 and oxaliplatin alone. Adenocarcinomas at the EGJ were only 10% of cases in this study. Nevertheless, this study supports the rationale that effective post-operative platinum/fluoropyrimidine chemotherapy alone without radiotherapy can also be administered after primary R0 resection of an adenocarcinoma in the EGJ that was incorrectly not pretreated.

The recommendations regarding adjuvant chemotherapy alone for adenocarcinoma of the GE junction are based on the S3 guideline on gastric cancer.^246^ The meta-analyses carried out in gastric cancer do not distinguish between gastric cancer proper and adenocarcinoma of the GE junction. A primary R0 resection (without preoperative chemotherapy) followed by adjuvant chemotherapy is not an adequate replacement for preoperative treatment and should be avoided as a planned therapy concept.

This relative negative recommendation is based on the guideline group’s belief that a preference for perioperative chemotherapy or preoperative radiochemotherapy should be established as the primary treatment strategy for adenocarcinomas of the EGJ. Adjuvant chemotherapy alone is not recommended as a primary treatment strategy because (i) adjuvant therapy alone is feasible in fewer patients due to their post-operative general condition^335^; (ii) the benefit of a perioperative therapy approach is better established by multi-center studies; and (iii) the survival improvement with perioperative therapy is greater than with adjuvant therapy alone (13.8% versus 5.8%).^445^

However, if no preoperative therapy has been initiated in patients due to inadequate staging or emergency surgery, adjuvant chemotherapy based on oxaliplatin and a fluoropyrimidine can be considered and offered for patients with primarily locally advanced tumor stage, and with positive lymph nodes, according to the available evidence.^246^

### Value of adjuvant immunotherapy

**Recommendation 66:** If residual tumor cells can still be detected histologically in the resection specimen (≥ypT1 or ≥ypN1) following neoadjuvant radiochemotherapy and R0 resection of squamous-cell carcinoma in the esophagus or adenocarcinoma in the esophagus or GE junction, adjuvant immunotherapy with nivolumab ought to be carried out for 1 year.

*Level of evidence: 2. Grade of recommendation: B*

*Consensus strength: strong consensus*

Adjuvant therapy measures, especially after multimodal therapy, have hardly been carried out in patients with esophageal cancer so far. This has changed with the introduction of immunotherapy. The international phase III CheckMate 577 trial investigated whether simple checkpoint blockade with nivolumab reduces the risk of recurrence after preoperative radiochemotherapy and R0 resection for those patients who did not achieve pathologically complete remission.^446^ This study enrolled patients with stage II/III esophageal cancer or carcinoma of the gastroesophageal junction (71% adenocarcinoma, 29% squamous-cell carcinoma). If tumor findings ≥ypT1 or ≥ypN1 were still present in the resected specimen after neoadjuvant RCT and surgery, patients were randomized to the nivolumab arm (*n* = 532; 240 mg every 2 weeks × 8, then 480 mg every 4 weeks, total duration of therapy 1 year) or the placebo arm (*n* = 262). The study showed that adjuvant immunotherapy is feasible over 12 months (median duration of therapy 10.1 months) and that patients’ quality of life is not negatively affected compared with placebo. The primary endpoint was changed during the study (before evaluation) from the combination of DFS and OS to DFS. The analysis showed a significant prolongation of DFS from a median of 11.0 months with placebo to 22.4 months with nivolumab (*P* < 0.001, HR 0.69, CI 0.56-0.86). Nivolumab primarily reduced the proportion of distant recurrences (29% versus 39%). Patients with carcinomas of both histologies benefited significantly (HR 0.61 for squamous-cell carcinomas, HR 0.75 for adenocarcinomas). Outcomes did not differ between PD-L1-positive (72% of patients) or -negative tumors. In a departure from other studies in the upper GI tract, only tumor cells were considered for this study (TPS score ≥1% or <1%).

DFS in the control arm appears unusually short at a median of 11 months. In a registry study from the Netherlands, the DFS for patients without pCR after radiochemotherapy and surgery is still a median of 19.2 months.^447^ The unfavorable DFS in the CheckMate-577 study could be due to the high proportion of high-risk patients with absent downsizing (ypT3-4) or persistently positive lymph nodes (ypN+), which was close to 60%. These data are missing from the preliminary publication of the Dutch study, making comparability difficult.

Based on the CheckMate-577 trial, nivolumab was approved in Europe for the adjuvant treatment of cancer of the esophagus or GE junction in adults with pathohistologically confirmed residual tumor disease after prior neoadjuvant radiochemotherapy and R0 resection.

### Aftercare and rehabilitation

**Recommendation 67 (EC):** Patients with esophageal cancer who have undergone potentially curative treatment should be offered structured follow-up care, provided that treatment decisions can be derived from this. In other cases, symptom-oriented follow-up care should be provided.

*Consensus strength: strong consensus*

Follow-up examinations should be adapted to the stage of the disease, the personal life situation, and the needs of the patient. There is a rationale for symptom-based follow-up in patients with esophagogastric carcinoma:

To detect dysfunction associated with recurrence or as benign complications of treatment.

To assess nutritional status and manage nutritional problems if necessary.

To offer psychological support for the patient and family, with appropriate medical interventions in conjunction with palliative care.

As part of symptom-based follow-up, a medical history, physical examination including weight, and blood count determination (supplemented with iron, ferritin, transferrin saturation, if necessary) should be carried out. These examinations are carried out at short notice at the beginning in order to detect complications more quickly and to ensure a balance of the nutritional intake.^448^^,^^449^

Despite no evidence of improved prognosis for all tumor stages, there are good reasons to recommend structured, holistic follow-up care in certain situations. For example, the early detection of locoregional recurrences after definitive radiochemotherapy in patients who are candidates for salvage resection is only possible as part of structured follow-up care. Patients with limited metastasis (oligometastasis) could also benefit from multimodal therapy including resection of the metastases or local ablative procedures. A meta-analysis indicated that the local treatment of oligometastasis appeared to be associated with survival benefits.^450^ Another reason for structured follow-up care is the justified and frequent desire of patients for information about their state of health.

There are no study-based recommendations on the scope and frequency of structured follow-up care. In line with a guideline adaptation to the S3 guideline on gastric cancer (2.0—August 2019), a semi-annual check-up with endoscopy and cross-sectional imaging (CT) is recommended in the first 2 post-operative years, and then at annual intervals until the end of the 5th perioperative year.

**Recommendation 68 (EC):** In the first 6 months, regular follow-up checks of the nutritional status including dietary advice should be carried out. Supplementation of oral energy intake with oral nutritional supplements or even tube feeding via an initially left fine-needle catheter jejunostomy can be recommended.

*Consensus strength: strong consensus*

After esophagectomy, 30% of patients lose >15% of their weight within 6 months.^360^ Adequate intake of micronutrients will be only achieved in 10% of esophagectomy patients who underwent reconstruction as gastric pull-up.^451^ The causes correspond to those of bariatric gastric tube formation as sleeve gastrectomy: loss of appetite, decreased enteral tolerance, possibly with dumping syndrome, meteorism, and diarrhea.

Severe weight loss 3 months after esophageal resection significantly influenced 5-year survival rate,^452^ and the weight loss could not be prevented even by enteral nutrition.^453^ Stabilization of body weight was only achieved after 4-6 months with continued enteral supplementation. In accordance with the guideline, nutritional counseling is strongly recommended and most patients are happy to accept it.^301^

The ESPEN guidelines recommend the implantation of a feeding tube during surgery, with fFCJ offering an option for longer-term supplementation.^306^ Therefore, it may be beneficial not to remove FCJ upon hospital discharge. If necessary, supplemental enteral nutrition can be provided via the FCJ, e.g. 500-1000 kcal/day overnight for several months. Appropriate instruction of the patient and family will in most cases allow care to be provided without nursing service. Although further weight loss is often unavoidable, at least an attenuation can be expected.^169^^,^^454^ Benefits were observed for continued oral nutritional supplementation at home for 8 weeks in terms of BMI, PG-SGA score, serum albumin, and immune parameters in elderly patients after esophageal resection.^455^

Home enteral nutrition appears superior compared with oral drink supplements in a recent meta-analysis, as a significant difference in weight loss and a reduction in the incidence of malnutrition was observed.^456^ It was also shown that weight loss was significantly lower in the home enteral nutrition group than in the control group without supplementation. Some dimensions of quality of life were also significantly better in the home enteral nutrition group.

Follow-up of nutritional status can be easily carried out with the observation of BMI. However, BMI is not sensitive to differences in body composition. Bioelectrical impedance analysis is an easily carried out noninvasive method, which can be carried out in an outpatient setting without any discomfort. The intraindividual course can be displayed and observed in a three-compartment model (extracellular mass, body cell mass, and fat mass). Of the body impedance, the ratio of extracellular mass to body cell mass and the phase angle are readily available values that provide reliable and valid information about the cellular content of the body. Ideally, the first examination is carried out at the time of diagnosis.

**Recommendation 69 (EC):** Patients with esophageal cancer should be motivated—within their means—to engage in physical activity. After completing primary therapy, all patients capable of rehabilitation should be offered follow-up treatment. The rehabilitative therapy should include medical, nursing, educational, training, and psychosocial measures adapted to the individual rehabilitation needs. In order to reduce the fatigue syndrome caused by cancer or tumor therapy, endurance training should be carried out that is geared to the individual’s ability to cope with stress.

*Consensus strength: consensus*

To maintain muscle mass in patients with esophageal cancer, light endurance training and specific muscle-building training should be recommended. There are no studies specific to patients with esophageal cancer. For cancer patients, exercises were shown to improve endurance and quality of life, preserve muscle mass, and reduce anxiety, depression, fatigue, pain and sleep disturbances.^457^, ^458^, ^459^ In order to reduce the fatigue syndrome caused by the tumor disease or therapy, endurance training should be carried out based on the individual exercise capacity.

Patients with esophageal cancer often suffer from weight loss and fatigue syndrome with physically impaired performance. Therefore, a specific rehabilitation program should be offered to improve nutritional status and cardiorespiratory endurance.^361^ The aim of rehabilitation is to eliminate as far as possible—or at least to compensate for—the consequences of tumors or therapy and to help patients to accept remaining disabilities with the goal of enabling them to participate in social life in a self-determined manner. For the rehabilitative measure, designated rehabilitation centers or clinics with GI and oncological expertise are to be preferred, which meet the standards of the quality assurance procedure of the German Pension Insurance and thus also include elements to motivate more sport and exercise.^459^

In Germany, rehabilitation is defined by the legislator as a social entitlement (SGB I, § 19). The type and scope of the required services are specified in SGB I (§ 29), SGB V (health insurance), SGB VI (pension insurance), SGB III (work entitlement), and also in the act on the harmonization of rehabilitation benefits (RehAnglG) and SGB IX. The need for rehabilitation after treatment of esophageal cancer is extremely variable and essentially depends on the type and extent of the surgical procedure and the consequences of the therapy. If there is a defined need for rehabilitation and an individual’s ability to undergo rehabilitation, rehabilitation procedures should take place as soon as possible after primary therapy.

## Palliative therapy

### Palliative systemic therapy: general

**Recommendation 70:** All patients should be offered palliative care following the diagnosis of a non-curable cancer, regardless of whether tumor-specific therapy is undertaken.

*Level of evidence: 1. Grade of recommendation: A*

*Consensus strength: strong consensus*

### Palliative first-line therapy: adenocarcinoma of the esophagus and esophagogastric junction

**Recommendation 71:** Patients with metastatic or locally advanced **adenocarcinoma of** the esophagus and EGJ that cannot be treated curatively should be offered systemic therapy. The therapeutic goal is to prolong survival and maintain quality of life.

*Level of evidence: 1a. Grade of recommendation: A*

*Consensus strength: strong consensus*

**Recommendation 72 (EC):** Before initiation of palliative systemic therapy of adenocarcinoma of the esophagus, HER2 status should be determined as a predictive factor for therapy with trastuzumab and PD-L1 CPS should be determined as a predictive factor for therapy with an immune checkpoint inhibitor.

*Consensus strength: strong consensus*

**Recommendation 73:** If HER2 status is negative and PD-L1 CPS < 5, a platinum (oxaliplatin or cisplatin)/fluoropyrimidine-containing two- or three-drug combination should be used.

*Level of evidence: 1a. Grade of recommendation: A*

*Consensus strength: strong consensus*

**Figure 6:**
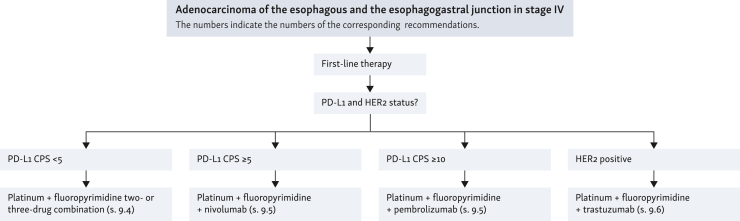
**Treatment algorithm for first-line treatment of metastatic or locally advanced, non-curatively treatable adenocarcinoma of the esophagus and gastroesophageal junction.** CPS, combined positive score; HER2, human epidermal growth factor receptor 2; PD-L1, programmed death-ligand 1.

**Recommendation 74:** In case of negative HER2 status and an elevated PD-L1 CPS cut-off value (for nivolumab PD-L1 CPS ≥ 5, for pembrolizumab PD-L1 CPS ≥ 10), a platinum (oxaliplatin or cisplatin)/fluoropyrimidine combination should be used together with one of the mentioned immune checkpoint inhibitors (Figure 6).

*Level of evidence: 1b. Grade of recommendation: A*

*Consensus strength: strong consensus*

**Recommendation 75:** For HER2-overexpressing tumors (IHC3+ or IHC2+ and FISH+), first-line cisplatin/fluoropyrimidine-based chemotherapy should be supplemented with trastuzumab.

*Level of evidence: 2. Grade of recommendation: A*

*Consensus strength: strong consensus*

In many randomized phase III trials for gastric cancer, the subgroup of adenocarcinomas of the gastroesophageal junction and distal adenocarcinomas of the esophagus represented a substantial proportion of the study population.^460^, ^461^, ^462^, ^463^, ^464^ Platinum- and fluoropyrimidine-based combination chemotherapy with docetaxel or epirubicin demonstrated significant improvement in terms of survival, time to tumor progression, and quality-of-life advantage over older chemotherapy protocols (FUP, FAMTX) (DCF versus FUP: median survival 9.2 months versus 8.6 months and progression-free survival 5.6 months versus 3.7 months and ECF versus FAMTX: median survival 8.9 months versus 5.7 months and FFS 7.4 months versus 3.4 months).^461^^,^^463^

Patients with negative HER2 status and a PD-L1 CPS < 5 should therefore be offered a platinum- and fluoropyrimidine-containing two- or three-drug combination. Among others, the following combinations can be considered: S-1/cisplatin or capecitabine/cisplatin (XP), infusional 5-FU, folinic acid, and cisplatin (PLF), epirubicin, cisplatin, capecitabine (ECX), epirubicin, oxaliplatin, capecitabine (EOX), epirubicin, cisplatin, infusional 5-FU (ECF), docetaxel, cisplatin, infusional 5-FU (DCF), infusional 5-FU/folinic acid and oxaliplatin (FLO), or the combination of 5-FU (infusional), folinic acid, oxaliplatin, and docetaxel (FLOT regimen).^460^, ^461^, ^462^, ^463^, ^464^, ^465^, ^466^, ^467^, ^468^, ^469^, ^470^, ^471^

For patients who do not qualify for platinum (oxaliplatin or cisplatin)-based therapy, infusional 5-FU, folinic acid, and irinotecan (FOLFIRI) is a treatment option. A randomized phase III trial compared ECX versus FOLFIRI. The primary endpoint of the study was TTF (time-to-treatment failure). This study showed a significant prolongation of TTF in favor of FOLFIRI (5.1 versus 4.2 months) and comparable median survival (9.5 versus 9.7 months). The side-effect profile of FOLFIRI was more favorable,^472^ compared with ECX.

General condition, age, concomitant diseases, toxicities of the therapy, and the individual situation of the patient have to be taken into account when selecting the therapy regimen. If a docetaxel-based triple combination is indicated, modified regimens should be preferred over the classic DCF regimen because the DCF regimen is associated with increased toxicity. Several phase II studies, some of them randomized, have shown that, among others, the combination of 5-FU (infusional), folinic acid, oxaliplatin, and docetaxel (FLOT regimen) has comparable activity to the DCF regimen with a more favorable side-effect profile.^427^^,^^470^

For elderly patients, several clinical trials have shown that the combination of oxaliplatin with a fluoropyrimidine (5-fluorouracil or capecitabine) can be carried out with respect to side-effects. The median age of patients in these studies ranged from 70 to 77 years. The median survival was 9.5-11.7 months.^473^, ^474^, ^475^, ^476^

Palliative chemotherapy can be initiated early after diagnosis of advanced disease due to its potential benefits in prolonging patients’ survival.^477^ In the meantime, phase III trials have been published establishing the value of immunotherapy in the systemic therapy of advanced non-curable adenocarcinoma of the esophagus, EGJ, and stomach.

In the KEYNOTE-590 trial, combining pembrolizumab with cisplatin and 5-FU showed a significant survival advantage over chemotherapy alone in HER-2 negative advanced adenocarcinoma of the esophagus and EGJ (AEG type 1) with a PD-L1 CPS ≥ 10 (HR 0.62, 13.5 versus 9.4 months).^478^ Based on these data, pembrolizumab was approved for non-curable HER2-negative adenocarcinoma of the esophagus or EGJ with a PD-L1 CPS ≥ 10 in combination with platinum- and fluoropyrimidine-based chemotherapy.

In the CheckMate-649 trial, patients with advanced HER2-negative adenocarcinoma of the esophagus, EGJ, or stomach received either oxaliplatin-based combination with a fluoropyrimidine [standard chemotherapy (*n* = 792) or nivolumab in addition to chemotherapy (*n* = 789)], or immunotherapy with nivolumab and ipilimumab alone. The majority (70%) of included patients had metastatic adenocarcinoma of the stomach.^479^ A significant improvement in survival was observed with the additional administration of nivolumab versus chemotherapy alone for carcinomas with a PD-L1 CPS ≥ 5 (14.4 versus 11.1 months; HR 0.71). Based on these data, nivolumab in combination with platinum- and fluoropyrimidine-based chemotherapy was approved for first-line treatment of advanced HER2-negative adenocarcinoma of the esophagus, EGJ, and stomach with a PD-L1 CPS ≥5.

#### HER2-overexpressing tumors

In addition to PD-L1 status, ‘HER2 status’ is considered a predictive factor. In a phase III trial (ToGA trial), the HER2 antibody trastuzumab improved OS and PFS in patients with HER2-positive advanced gastric cancers and adenocarcinomas of the EGJ whose tumors were either immunohistochemically HER2-positive (IHC 3+) or had amplification of the *HER2* gene on FISH+.^246^ Trastuzumab is formally approved only with a cisplatin/fluoropyrimidine combination. However, in the KEYNOTE-811 trial, trastuzumab was also used in combination with oxaliplatin/fluoropyrimidines in HER2-positive gastric or gastroesophageal junction adenocarcinoma.^480^

The JACOB phase III study (*n* = 780) assessed the value of pertuzumab in first-line therapy for patients with HER2-positive advanced gastric cancer and adenocarcinoma of the EGJ.^481^ The primary endpoint of significant survival extension was not met. The median survival was 17.5 months for the pertuzumab-based combination versus 14.2 months for standard therapy with trastuzumab, cisplatin, fluoropyrimidine (capecitabine or 5-FU) (HR 0.84, *P* = 0.0565). Thus, there is no indication for the additional use of pertuzumab in the therapy of HER2-positive advanced gastric cancer and adenocarcinoma of the EGJ.

The GATSBY phase III study (*n* = 345) evaluated the place of trastuzumab emtansine (T-DM1) in second-line therapy for patients with HER2-positive advanced gastric cancer and adenocarcinoma of the EGJ.^482^ There was no survival benefit for trastuzumab emtansine over therapy with a taxane (docetaxel, paclitaxel). Median survival was 7.9 months for trastuzumab emtansine and 8.6 months for the taxane-treated patients (HR 1.15, *P* = 0.86).

### Palliative first-line therapy: squamous-cell carcinoma of the esophagus

**Recommendation 76 (EC):** Before initiation of palliative systemic therapy of squamous-cell carcinoma, PD-L1 CPS should be determined as a predictive factor for therapy with an immune checkpoint inhibitor.

*Consensus strength: strong consensus*

**Figure 7:**
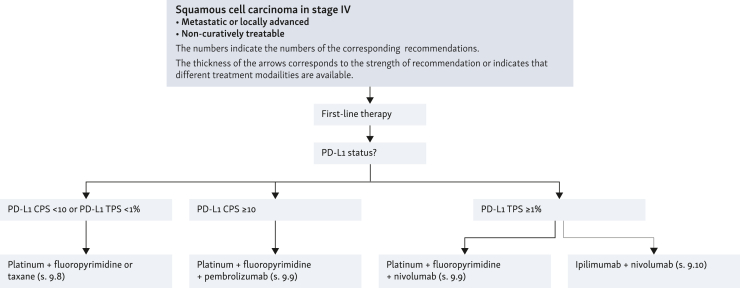
Treatment algorithm for first-line treatment of metastatic or locally advanced, non-curatively treatable squamous-cell carcinoma of the esophagus. CPS, combined positive score; PD-L1, programmed death-ligand 1; TPS, tumor proportion score.

**Recommendation 77 (EC):** Patients with metastatic or locally advanced squamous-cell carcinoma of the esophagus with a PD-L1 CPS < 10 and a PD-L1 TPS < 1% that cannot be treated curatively may be offered palliative systemic chemotherapy (Figure 7). The therapeutic goal is to maintain quality of life. Here, a combination therapy of a platinum derivative with a fluoropyrimidine or a taxane can be used.

*Consensus strength: strong consensus*

**Recommendation 78:** In patients with metastatic or locally advanced squamous-cell carcinoma of the esophagus that is not curatively treatable and has a PD-L1 CPS ≥ 10 or PD-L1 TPS ≥ 1%, platinum/fluoropyrimidine chemotherapy should be used in conjunction with an immune checkpoint inhibitor (pembrolizumab PD-L1 CPS ≥ 10, nivolumab PD-L1 TPS ≥ 1%) (Figure 7).

*Level of evidence: 2. Grade of recommendation: A*

*Consensus strength: strong consensus*

**Recommendation 79:** In patients with metastatic or locally advanced, non-curatively treatable squamous-cell carcinoma of the esophagus with a PD-L1 TPS ≥ 1%, the combination of nivolumab/ipilimumab ought to be used as the sole immunotherapy.

*Level of evidence: 2. Grade of recommendation: B*

*Consensus strength: strong consensus*

Patients with metastatic or locally advanced (non-curable squamous-cell carcinoma of the esophagus) who do not qualify for immunotherapy should be offered systemic palliative chemotherapy with the goal of maintaining quality of life. Data are very limited regarding randomized clinical trials and often refer only to a subpopulation of patients.^460^^,^^461^^,^^463^^,^^464^ In the meantime, the value of immunotherapy was also established in the first-line treatment of advanced squamous-cell carcinoma by several phase III trials.

In the KEYNOTE-590 trial, for first-line therapy of advanced squamous-cell carcinoma of the esophagus and HER-2-negative adenocarcinoma (AC) of the esophagus and gastroesophageal junction (AEG type 1), a significant survival benefit was shown for the combination of pembrolizumab with cisplatin and 5-FU compared with chemotherapy alone in tumors with a CPS ≥ 10 (survival squamous-cell carcinoma PD-L1 CPS ≥ 10, HR 0.57; 13.9 versus 8.8 months, *P* < 0.0001 in favor of the additional administration of pembrolizumab).^478^ Pembrolizumab was approved for locally advanced, non-curable squamous-cell carcinoma of the esophagus and HER2-negative adenocarcinoma of the esophagus or gastroesophageal junction with a CPS ≥ 10 in combination with platinum- and fluoropyrimidine-based chemotherapy.

The CheckMate-648 trial demonstrated a significant survival benefit for the combination of nivolumab with cisplatin and 5-FU versus chemotherapy alone for first-line treatment of advanced squamous-cell carcinoma of the esophagus. Study inclusion was independent of PD-L1 status. For PD-L1-positive (defined here as PD-L1 TPS ≥ 1%) carcinomas, the HR was 0.54 and median survival was 15.4 versus 9.1 months for chemotherapy alone. The immunotherapy-alone arm with nivolumab and ipilimumab also significantly prolonged survival in PD-L1-positive tumors compared with cisplatin and 5-FU (HR 0.64; 13.7 versus 9.1 months, *P* = 0.0010), but with survival curves crossing in the first months to the disadvantage of immunotherapy.^483^

In addition, nivolumab in combination with ipilimumab has also been approved for first-line treatment of unresectable advanced, recurrent, or metastatic squamous-cell carcinoma of the esophagus with TPS PD-L1 expression ≥ 1% in adults.^483^

In another randomized ESCORT-1 phase III study, the programmed cell death protein 1 (PD-1) immune checkpoint inhibitor camrelizumab in combination with platinum-based chemotherapy (cisplatin and paclitaxel) led to a significant improvement in survival in first-line therapy compared with chemotherapy alone [median survival: 15.3 months (95% CI 12.8-17.3 months) versus 12.0 months (95% CI 11.0-13.3 months); HR 0.70, 95% CI 0.56-0.88, *P* = 0.001].^484^

In the current literature, there are few publications addressing surgical therapy for metastatic or locally recurrent esophageal cancer. While randomized trials or cohort studies are not available, a few case series or case reports are available that have evaluated the value of surgical therapy in this oncologic setting.^44^^,^^226^^,^^351^^,^^423^^,^^485^, ^486^, ^487^, ^488^, ^489^ In the context of surgical therapy for metastatic esophageal cancer, the publications available to date are mainly concerned with the management of metachronous lung or liver metastases. The studies suggest that surgical treatment of a metachronous, solitary lung metastasis can be carried out within the framework of individual therapy planning, but that surgical treatment should rather not be carried out in the case of metachronous liver metastases or multilocular lung metastases. For example, Ichida et al.,^487^ showed in 26 patients with metachronous liver metastases or 27 patients with pulmonary metastases who previously underwent esophagectomy that although patients with solitary pulmonary metastases may have a prognostic advantage, there tends to be no relevant benefit, especially for patients with liver metastases.

In patients with locally recurrent esophageal cancer, previous analyses have almost exclusively analyzed patients after definitive radiochemotherapy who underwent salvage esophagectomy.^44^^,^^226^^,^^351^^,^^423^^,^^488^^,^^489^ Most of the studies show a relevant increase in morbidity or mortality for this patient group and therefore such surgical treatment should only be carried out in the context of individual therapy planning. The British guidelines recommend an individualized therapeutic approach for local recurrence after definitive radiochemotherapy, but a surgical approach for solitary distant metastases to the lung or liver is not discussed.^44^

### Palliative system therapy: second- and third-line therapy

**Recommendation 80:** In patients with metastatic or locally advanced adenocarcinoma of the esophagus not amenable to curative treatment and adequate general condition, second- and third-line systemic therapy ought to be given.

*Level of evidence: 1b. Grade of recommendation: B*

*Consensus strength: strong consensus*

**Recommendation 81:** In patients with metastatic or locally advanced, non-curatively treatable adenocarcinoma of the esophagus and sufficient general condition, the MSI-high and/or dMMR status should be determined in the event of tumor progression under or recurrence after first-line therapy. Due to the high efficacy of immune checkpoint inhibitors in tumors with MSI-high or with dMMR, these patients ought to be offered treatment with a checkpoint inhibitor after failure of first-line therapy if no immunotherapy has been used previously.

**Cave: Consider off-label use.** *Level of evidence: 2. Grade of recommendation: B*

*Consensus strength: consensus*

The treatment regimen depends on the respective prior therapy. The goal of therapy is to prolong survival, time to tumor progression, and preservation of quality of life.

There are now several phase III studies showing prolongation of OS and, in some cases, preservation of quality of life with second- and third-line therapy. This is true for the following single-agent cytotoxic agents irinotecan, paclitaxel, and docetaxel,^490^, ^491^, ^492^ as well as for the vascular endothelial growth factor receptor 2 (VEGFR2) antibody ramucirumab alone or in combination with paclitaxel.^493^^,^^494^

After progression under first-line chemotherapy, the VEGFR2 antibody ramucirumab was used as monotherapy (REGARD study) compared with best supportive care,^493^ or in combination with paclitaxel (RAINBOW study),^494^ compared with paclitaxel monotherapy. OS was significantly improved in both the REGARD study (5.2 months versus 3.8 months, HR 0.776) and the RAINBOW study (9.6 months versus 7.4 months, HR 0.807). Patients also appeared to benefit in terms of quality of life. Both studies included patients with gastric cancer or adenocarcinoma of the EGJ. The RAINBOW trial also showed a higher response rate in the combination group.

The trifluridine/tipiracil (TAS-102) combination showed a significant survival benefit in the TAGS trial in patients (*n* = 505) with advanced adenocarcinoma of the stomach (71% of patients) or EGJ (29% of patients), with 5.7 months versus 3.6 months in the placebo group (HR 0.69, *P* = 0.00058) in third-line or follow-up therapy. However, in a planned subgroup analysis, the difference in survival for transitional cancers was not significant. In the TAGS trial, >90% of patients in the trifluridine/tipiracil treatment group were extensively pretreated (platinum derivative 100%, fluoropyrimidine >99%, taxane 92%).^495^

#### Adenocarcinomas with MSI-high status or with a mismatch repair deficiency (dMMR)

Patients with gastric carcinoma or adenocarcinoma of the GE junction with MSI-high or dMMR status show a high response to checkpoint inhibitors regardless of the line of therapy.^443^^,^^479^^,^^496^^,^^497^

In the global KEYNOTE-061 study, pembrolizumab was evaluated against second-line therapy with paclitaxel in patients with adenocarcinoma of the stomach or GE junction.^498^ In the MSI-high subgroup (5.3% of patients, 27 of 514 assessable patients), the administration of pembrolizumab led to a significant increase in survival time and progression-free time compared to chemotherapy with paclitaxel.

Pembrolizumab has been approved by the European Medicines Agency (EMA) for patients with tumors with MSI-high status or dMMR as monotherapy for unresectable or metastatic gastric cancer (but not for esophageal adenocarcinoma) with disease progression during or after at least one prior therapy.

#### HER2-overexpressing tumors

The antibody–drug conjugate trastuzumab deruxtecan, consisting of the anti-HER2 antibody trastuzumab and a topoisomerase inhibitor, has high efficacy in patients with advanced, relapsed, or refractory HER2-positive adenocarcinoma of the stomach or GE junction.^499^ Treatment with trastuzumab deruxtecan in extensively trastuzumab-pretreated Asian patients with HER2 overexpression (IHC3+ or gene amplification) led to a significant increase in the remission rate (51% versus 14%) compared with chemotherapy (irinotecan, paclitaxel) and to a prolongation of median survival (12.5 months versus 8.4 months).^500^ The results of the DESTINY-Gastric04 phase III trial (trastuzumab deruxtecan versus ramucirumab/paclitaxel) in advanced adenocarcinoma of the stomach or GE junction with HER2 overexpression after failure of trastuzumab-based therapy are still pending.

There is currently no EMA approval for treatment with trastuzumab deruxtecan in advanced adenocarcinoma of the stomach or GE junction.

**Recommendation 82:** Patients with metastatic or locally advanced squamous-cell carcinoma of the esophagus that cannot be treated curatively and sufficient general condition ought to receive second-line therapy with an immune checkpoint inhibitor, if there has been no previous immunotherapy.

*Level of evidence: 2. Grade of recommendation: B*

*Consensus strength: strong consensus*

There are no robust data showing efficacy of second-line chemotherapy in squamous-cell carcinoma of the esophagus. Only phase II studies exist with agents such as taxanes, platinum derivatives, and irinotecan, as well as those with older agents such as mitomycin C.^501^

Several phase III trials show activity of immunotherapy in advanced squamous-cell carcinoma of the esophagus. In the Attraction-3 trial, patients with advanced squamous-cell carcinoma of the esophagus showed a survival benefit of 2.5 months for nivolumab administration after failure of 5-FU and platinum-based chemotherapy versus the control group receiving further chemotherapy with paclitaxel or docetaxel (10.9 months versus 8.4 months, HR 0.77, *P* = 0.019).^502^ One-year survival was 47% for nivolumab and 34% for the control group. However, survival curves intersected in the first months to the disadvantage of immunotherapy. Therapy with nivolumab was associated with better treatment tolerability and quality of life. It should be noted that the Attraction-3 trial included almost exclusively Asian patients (96%).

Nivolumab was approved for the treatment of patients with metastatic or locally advanced, non-curable squamous esophageal disease after prior fluoropyrimidine- and platinum-based chemotherapy regardless of PD-L1 status.

In the RATIONALE-302 phase III trial, the anti-PD-1 antibody tislelizumab showed a significant survival benefit (8.6 versus 6.3 months, HR 0.70, *P* = 0.0001) over second-line chemotherapy (paclitaxel, docetaxel, irinotecan) for patients with advanced squamous-cell carcinoma.^503^

In a large Asian phase III trial (ESCORT) of 457 patients, a significant survival benefit (8.3 versus 6.2 months; HR 0.71, *P* = 0.001) was shown for camrelizumab immunotherapy versus second-line chemotherapy (docetaxel or irinotecan).^504^

Similarly in the KEYNOTE-181 study, patients with squamous-cell carcinoma and a CPS ≥ 10 receiving therapy with pembrolizumab showed prolonged survival compared to chemotherapy with docetaxel or irinotecan in the second-line setting.^505^

### Palliative radio(chemo)therapy

**Recommendation 83 (EC):** Percutaneous radiotherapy of esophageal cancer—if necessary in combination with simultaneous chemotherapy—can be used for local symptoms (e.g. bleeding, stenosis, compression) as part of multidisciplinary care.

*Consensus strength: consensus*

The goal of palliative treatment of esophageal cancer is to relieve typical symptoms of advanced disease such as dysphagia, pain, or bleeding. Prolongation of survival is not expected from palliative therapy.^506^ In this respect, the subjective relief of symptoms from the patient’s point of view is of decisive importance for the evaluation of palliative therapy procedures. Therapy effect and toxicity of a therapy are equally captured.

Amdal et al. reviewed 28 studies on the patient assessments of palliative therapies.^505^ The superiority of radiochemotherapy was shown compared with polychemotherapy and radiotherapy alone. While accelerated radiochemotherapy was associated with high acute toxicity, normofractionated radiotherapy with simultaneous administration of 5-FU showed a good response with low toxicity.^506^^,^^507^

The decision for local palliative therapy (percutaneous radiochemotherapy, brachytherapy, or stent implantation) should be coordinated in a multidisciplinary team.^508^

### Palliative brachytherapy

**Recommendation 84:** Palliative brachytherapy ought to be offered as part of the multidisciplinary care of patients with esophageal cancer to relieve dysphagia, in combination with percutaneous radiochemotherapy, or stent implantation when appropriate.

*Level of evidence: 1a. Grade of recommendation: B*

*Consensus strength: strong consensus*

As shown in two randomized trials, palliative brachytherapy can improve dysphagia and quality of life in patients with unresectable esophageal cancer.^509^ Compared with stent implantation, the effect of brachytherapy occurs later but lasts longer. A combination of stent implantation with brachytherapy is possible and ought to be used especially in patients who have a longer projected life expectancy. In this situation, brachytherapy may prolong the duration of the stent’s effect on dysphagia.

If dysphagia is pronounced, stent implantation should be carried out first, followed by brachytherapy 1-4 weeks later.^507^ In cases of mild dysphagia, brachytherapy alone produces a sustained palliative effect and is considered the treatment of choice because of the lower complication rate compared with stent implantation.

The effect of brachytherapy can be improved by additional percutaneous radiotherapy or radiochemotherapy.^510^ The decision for palliative brachytherapy and its combination with stent implantation or percutaneous radiochemotherapy should be coordinated in a multidisciplinary treatment team.

### Endoscopic stent application

**Recommendation 85:** A self-expanding metal stent ought to be used for rapid relief of dysphagia in patients with esophageal cancer.

*Level of evidence: 1a. Grade of recommendation: B*

*Consensus strength: consensus*

The use of SEMS for rapid relief of dysphagia has been established as a standard therapy in recent years.^44^^,^^511^ This was also supported by a meta-analysis of 16 randomized trials with a total of 1027 patients.^511^ After implantation of a SEMS, about two-third of patients with initial stenosing esophageal cancer can take in solid food.^512^ However, 10% of patients had worse dysphagia than initially by 6 weeks.^513^ Small diameter stents (18 mm) appear to be effective similarly to wider stents (24 mm) but induce less pain after implantation.^513^

A variety of SEMS are available. Uncovered SEMS are initially as effective as covered stents, but there is an increased risk of tumor tissue growing in through the meshes, so tumor stenosis may soon return.^514^

However, one advantage of uncovered SEMS is a lower migration rate. The use of partially covered SEMS seems to combine the advantages of both types, but little robust data exist on this. A prospective randomized multicenter Dutch study with 98 patients showed a similar openness rate of fully covered and partially covered SEMS in palliative therapy for esophageal cancer. The complication rate was also not significantly different between the two stent types.^515^ In a recent meta-analysis of five studies with 542 patients, the equivalence of fully covered and partially covered SEMS was confirmed.^516^ Covered metal stents are the treatment of choice for esophageal cancer with fistulas and have an occlusion rate of 77%-92%.^516^^,^^517^

Self-expandable plastic stents are obsolete and no longer used.^518^ Patients with a tumor in the distal esophagus often suffer from massive GE reflux after stent implantation. SEMS with anti-reflux valve appear not to provide any benefit compared with normal SEMS. Two meta-analyses and a current prospective randomized study showed no difference between SEMS with and without anti-reflux valve in terms of clinical outcome, reflux symptoms, and complication rate.^519^, ^520^, ^521^

Initial study results on the new radioactive SEMS show a longer openness of radioactive SEMS compared with conventional SEMS. In a meta-analysis of six randomized studies with 403 patients, the duration until re-stenosis was significantly longer in the patient group with radioactive SEMS. Surprisingly, the group with radioactive SEMS showed significantly better survival. The complication rate did not differ significantly between the two stent types.^522^ In order to make a general recommendation on the use of radioactive SEMS, the results of further studies should be awaited.

**Recommendation 86:** With an inserted SEMS, simultaneous percutaneous radiotherapy ought to be avoided because it is associated with an increased complication rate.

*Level of evidence: 4. Grade of recommendation: B*

*Consensus strength: strong consensus*

The insertion of a SEMS during simultaneous radiotherapy should be avoided due to the increased complication rate. However, in special selected cases, a fully covered SEMS may be inserted to alleviate marked dysphagia and removed again when radiotherapy or radiochemotherapy is started.

In a retrospective work-up of 997 patients, prior radiochemotherapy was shown to be the only risk factor for a stent-associated complication.^373^ A small Asian study suggests that this results in a significantly increased rate of fistula.^522^ In addition, a malignant fistula arising from radiotherapy appears to be a predictive factor for therapeutic failure of fistula closure.^523^ The ESGE guideline also argues against simultaneous external radiotherapy with stent in place, taking into account the increased rate of side-effects.^524^

A prospective randomized phase III trial from the UK with 220 patients compared stent implantation alone with stent implantation and additional radiotherapy with 20-30 Gy.^525^ Concurrent palliative radiotherapy did not offer additional benefits for dysphagia. The stent-related complications were not different in the two groups. The additional radiotherapy only had a positive effect on tumor-related bleeding complications.^525^

An alternative for palliation of dysphagia in esophageal cancer is brachytherapy. Compared to stents, relief of dysphagia is delayed, but brachytherapy was superior to SEMS implantation at 3 months.^509^^,^^526^ Stent implantation is more cost-effective than brachytherapy.^527^ A combination of both methods seems to be effective and safe, but there is little prospective data for this.^528^

### Value of intraluminal local therapy

**Recommendation 87 (EC):** Intraluminal thermoablative therapy in patients with exophytic esophageal cancer in the palliative setting can be considered.

Additive brachytherapy or radiotherapy after local tumor ablation may prolong the dysphagia-free interval.

*Consensus strength: strong consensus*

Several methods are available for intraluminal tumor therapy. Palliative therapy using PDT and intratumoral alcohol injection is obsolete mainly due to the high complication rate and the availability of more effective alternative methods.^513^^,^^529^

For laser therapy, most data exist demonstrating its effectiveness^529^, ^530^, ^531^, ^532^, ^533^, ^534^; however, nowadays a laser is available in very few endoscopy departments.

The most commonly used method for thermoablative therapy in Germany today is argon plasma coagulation (APC).^535^, ^536^, ^537^ It seems to be similarly effective as laser therapy, but there are hardly any comparative studies for this.^535^^,^^538^ A mean of 2.3 sessions is required for tumor ablation. The rate of severe complications, such as perforations, was <1% in one series.^535^^,^^537^ More recent cohort studies suggest that cryospray therapy can achieve a significant improvement in dysphagia. However, this procedure has so far only been used in the United States.^539^^,^^540^

A prospective randomized study of 93 patients compared APC therapy alone with combination therapy of APC plus high-dose brachytherapy and APC plus PDT.^540^ The most effective therapy with the lowest complication rate was a combination of APC with brachytherapy. Recurrence of dysphagia occurred only after 88 days. Thus, the dysphagia-free interval was significantly longer than in the two comparison groups (59 and 35 days).

There are currently no studies on the question of whether local ablation of metastases in patients with esophageal cancer provides a demonstrable advantage over other therapeutic procedures such as palliative chemotherapy and best supportive care in terms of quality of life and survival. Only case reports or collective casuistry can be found in the literature. This is true for procedures such as transarterial chemoembolization, selective internal radiotherapy, RFA, laser-induced thermotherapy, microwave ablation, and stereotactic radiotherapy. These procedures should therefore be evaluated in controlled, prospective studies in this indication.

## Psycho-oncology

**Recommendation 88 (EC):** The psycho-oncological care of patients with esophageal cancer should be an integral part of oncological diagnostics, therapy, and aftercare and pose an interdisciplinary task for all professional groups involved in oncology.

*Consensus strength: strong consensus*

## Funding

This work was supported by the ‘Deutsche Krebshilfe’ [grant number 70112585] for Coordination and update of the guideline ‘German guidelines for the diagnosis and treatment of squamous-cell carcinoma and adenocarcinoma of the esophagus—version 4.0’ within the framework of the ‘Leitlinienprogramm Onkologie’.

## Disclosure

**CB:** Payment or honoraria for lectures, presentations, speakers bureaus, manuscript writing or educational events—Stryker, Intuitive, Medtronic, medUpdate, AstraZeneca. **WF:** Payment or honoraria for lectures, presentations, speakers bureaus, manuscript writing or educational events—Falk, Norgine. **MF:** Other financial or non-financial interests: Guideline methodologist (INGUIDE certified); Program Manager German Guideline program in Oncology. **LG:** Leadership or fiduciary role in other board, society, committee or advocacy group, paid or unpaid— Member of the board of abdominal and gastrointestinal imaging of the German Roentgen society. **JH:** Support for attending meetings and/or travel—Intuitive Surgical. **RL:** honoraria—Astellas; advisory board honoraria—MSD; advisory board (Switzerland)—MSD. **FL:** Grants or contracts from any entity—AstraZeneca, Beigene, BMS, Daiichi-Sankyo, Gilead; consulting fees—Amgen, Astellas, Bayer, Beigene, Biontech, BMS, Eli Lilly, MSD, PAGE, Roche, Servier; payment or honoraria for lectures, presentations, speakers bureaus, manuscript writing or educational events—Astellas, AstraZeneca, BMS, Daiichi-Sankyo, Eli Lilly, Medscape, MedUpdate, Merck, MSD, Roche, Servier, StreamedUp!; support for attending meetings and/or travel—Daiichi-Sankyo; participation on a data safety monitoring board or advisory board—Biontech; leadership or fiduciary role in other board, society, committee or advocacy group, paid or unpaid—European Society for Medical Oncology, International Gastric Cancer Association, International cancer Foundation. **DL:** participation on a data safety monitoring board or advisory board—CARDIA Trial DSMB. **SL:** participation on a data safety monitoring board or advisory board—MSD, BMS, BeiGene, Daiichi-Sankyo, AstraZeneca. **PL:** leadership or fiduciary role in other board, society, committee or advocacy group, paid or unpaid—CEO German Society of Gastroenterology. **AGM:** honoraria for lectures—Falk Foundation; honoraria—AbbVie, Takeda; participation on a data safety monitoring board or advisory board—Luvos. **HJM:** leadership or fiduciary role in other board, society, committee or advocacy group, paid or unpaid—Board of the German Society of Surgery, President of Professional Association of German Surgery, UEMS Surgical Section. **NHN:** research grant—Novocure; speaker honoraria—Novocure, Sun Medical, Merck; ESTRO travel grant—Merck; participation on a data safety monitoring board or advisory board—Novocure; in vitro preclinical research platform—Novocure; Xevinapant for preclinical research—Merck. **MN:** third party funding by German Cancer Aid Recipient—institutional; external lecturer honoraria—Berlin School of Public Health; other financial or non-financial interests—German Network Evidence Based Medicine (member) Guidelines International Network/GRADE Working Group (member). **OP:** payment or honoraria for lectures, presentations, speakers bureaus, manuscript writing or educational events—Medtronic, Boston Scientific, Olympus, AbbVie, Falk, Aohua, BMS. **RP:** payment or honoraria for lectures, presentations, speakers bureaus, manuscript writing or educational events—Falk Foundation, lecture and travel fee, med Update GmbH, lecture and travel fee, wikonect GmbH, lecture and travel fee; advisory Board—Bremer Krebsgesellschaft. **HS:** participation on a data safety monitoring board or advisory board—GHSG AERN Study on Nivolumab in recurrent Hodgkin’s Lymphoma. **TS:** consulting fees—Lilly, Amgen, Lilly, AstraZeneca; payment or honoraria for lectures, presentations, speakers bureaus, manuscript writing or educational events—Amgen, Merck, AstraZeneca, BMS, MSD. **MSta:** consulting fees—AstraZeneca, Bristol-Myers & Squibb, Daiichi-Sankyo, Lilly Deutschland, Merck, Sharp & Dohme, Roche Pharma, Servier; payment or honoraria for lectures, presentations, speakers bureaus, manuscript writing or educational events—Amgen, Bristol-Myers & Squibb, Lilly Deutschland, Roche Pharma; support for attending meetings and/or travel—Daiichi-Sankyo, Ipsen Pharma; participation on a data safety monitoring board or advisory board—AstraZeneca, Bristol-Myers & Squibb, Daiichi-Sankyo, Lilly Deutschland, Merck, Sharp & Dohme, Roche Pharma, Servier. **MStu:** research clinical trial to institution—AstraZeneca. **PTP:** consulting fees for advisory board—BMS, AstraZeneca, Novartis, Roche, MSD, Merck Serono, Daiichi, Lilly, Servier, Astellas; payment or honoraria for lectures, presentations, speakers bureaus, manuscript writing or educational events—BMS, MSD; support for attending meetings and/or travel—AstraZeneca, Daiichi. **JT:** consulting fees—Amgen, AstraZeneca, Bayer Healthcare, Bristol-Myers Squibb, Eisai, EXACT TherapeuIcs AS, InsItut für Qualitätssicherung und Transparenz im Gesundheitswesen (IQTiG), InsItut für Qualität und Wirtschaglichkeit im Gesundheitswesen (IQWiG), Ipsen, Lilly Imclone, Merck Serono, Merck Sharp & Dome, onkowissen.de, Roche and Servier; payment or honoraria for lectures, presentations, speakers bureaus, manuscript writing or educational events—Amgen, AstraZeneca, Bristol-Myers Squibb, Eisai, Ipsen, Merck Serono, medupdate, Merck Sharp & Dome, Lilly Imclone, NaIonale Gesundheitsakademie, onkowissen.de, Roche, Servier and streamedup!; support for aFending meetings and/or travel—Roche, Servier; partcipation on a data safety monitoring board or advisory board—Oncolytics Biotech. **UV:** payment or honoraria for lectures, presentations, speakers bureaus, manuscript writing or educational events—Roche Pharma AG, Bristol Meyers Squibb, Amgen GmbH, AstraZenaca GmbH, MSD, Forum fur Medizinische Fortbildung, Interplan Congress Management, I-Med Institute, RG-Gesellschaft fur Information und Organisation mbH, Asklepios Kliniken Hamburg GmbH, Universitatsklinikum Hamburg-Eppendorf, Universitatsklinikum Schleswig Holstein, MVZ HPH institute fur Pathologie, Tumorzentrum West-Nierdersachsen e.V., PIUS-Hospital Oldenburg; participation on a data safety monitoring board or advisory board—Servier Deutschland GmbH, Sanofi Aventis Deutschland, Amgen GmbH, Daiichi-Sankyo Deutschland GmbH. **AW:** payment or honoraria for lectures, presentations, speakers bureaus, manuscript writing or educational events— Abbott, Baxter, B. Braun, Fresenius Kabi, Falk Foundation, Memomed; receipt of equipment, materials, drugs, medical writing, gifts or other services—Seca; other financial or non-financial interests—B. Braun, Mucos. **TZ:** payment or honoraria for lectures, presentations, speakers bureaus, manuscript writing or educational events—AstraZeneca. All other authors have declared no conflicts of interest.
