# Supplementary material for: German guidelines for the diagnosis and treatment of squamous-cell carcinoma and adenocarcinoma of the esophagus—version 4.0
Source: ESMO Gastrointest Oncol. 2025 Jan 6;7:100112. doi: 10.1016/j.esmogo.2024.100112 (PMC12836548; doi:10.1016/j.esmogo.2024.100112)
Supplement: Supplemental Tables [file mmc1.docx]

# **Supplemental Materials**

# **List of Supplemental Tables**

## **Table S1:** Oxford evidence grading scheme (Version 2009)

## **Table S2:** Evidence classification according to Oxford 2011

## **Table S3:** Clinical classification of esophageal cancers, including carcinomas of the esophagogastric junction according to TNM classification

## **Table S4:** Stage grouping of the TNM classification of esophageal cancers, including carcinomas of the esophagogastric junction - Squamous cell carcinoma

## **Table S5:** Stage grouping of the TNM classification of esophageal cancers, including carcinomas of the esophagogastric junction - Adenocarcinoma

# **Supplemental Tables**

## **Table S****1. Oxford evidence grading scheme (Version 2009).**

| Level | Therapy/Prevention, Aetiology/Harm | Prognosis | Diagnosis | Differential diagnosis/symptom prevalence study |
| --- | --- | --- | --- | --- |
| 1a | SR (with homogeneity) of RCTs | SR (with homogeneity) inception cohort studies; CDR validated in different populations | SR (with homogeneity) of Level 1 diagnostic studies; CDR with 1b studies from different clinical centers | SR (with homogeneity) of prospective cohort studies |
| 1b | Individual RCT (with narrow Confidence Interval) | Individual inception cohort study with > 80 | Validating cohort study with good reference standards; or CDR tested within one clinical centre | Prospective cohort study with good follow-up |
| 2a | SR (with homogeneity) of cohort studies | SR (with homogeneity) of either retrospective cohort studies or untreated control groups in RCTs | SR (with homogeneity) of Level >2 diagnostic studies | SR (with homogeneity) of Level 2b and better studies |
| 2b | Individual cohort study (including low quality RCT; e.g., <80 | Retrospective cohort study or follow-up of untreated control patients in an RCT; Derivation of CDR or validated on split-sample only | Exploratory cohort study with good reference standards; CDR after derivation, or validated only on split-sample or databases | Retrospective cohort study, or poor follow-up |
| 2c | “Outcomes” | “Outcomes” |  | Ecological studies |
| 3a | SR (with homogeneity) of case-control studies |  | SR (with homogeneity) of 3b and better studies | SR (with homogeneity) of 3b and better studies |
| 3b | Individual Case-Control Study |  | Non-consecutive study; or without consistently applied reference standards | Non-consecutive cohort study; or very limited population |
| 4 | Case-series (and poor quality cohort and case-control studies) | Case-series (and poor quality prognostic cohort studies) | Case-control study, poor or non-independent reference standard | Case-series or superseded reference standards |
| 5 | Expert opinion without explicit critical appraisal, or based on physiology, bench research or “first principles” | Expert opinion without explicit critical appraisal, or based on physiology, bench research or “first principles” | Expert opinion without explicit critical appraisal, or based on physiology, bench research or “first principles” | Expert opinion without explicit critical appraisal, or based on physiology, bench research or “first principles” |

## **Table S2. Evidence classification according to Oxford 2011.**

| Question | Step 1 (Level 1) | Step 2 (Level 2) | Step 3 (Level 3) | Step 4 (Level 4) | Step 5 (Level 5) |
| --- | --- | --- | --- | --- | --- |
| **How frequent is the problem** | Local and current randomized samples from surveys (or censuses) | Systematic reviews of surveys that allow adjustment for local conditions. | Local non-random sample | Case series | Not available |
| Is the diagnostic or monitoring test accurate? (Diagnostic) | Systematic review of cross-sectional studies with consistently applied reference standard and blinding | Single cross-sectional studies with consistently applied reference standard and blinding | Non-consecutive studies or studies without a consistently applied reference standard. | Case-control studies, or inferior, non-independent reference standard | Mechanism-based reasoning |
| What will happen without therapy? (Prognosis) | Systematic Reviews of Inception Cohort Studies | Inception cohort studies | Cohort studies or control arms of randomized trials* | Case series or case-control studies, or inferior prognostic cohort studies | Not available |
| Does the intervention help? Treatment benefit | Systematic reviews of randomised trials or n=1 trials | Randomized trials or observational studies with dramatic effect | Non-randomized controlled cohort/follow-up studies | Case series or case-control studies, or historical controlled studies | Mechanism-based reasoning |
| What are the common harms/disadvantages of the intervention? Treatment disadvantage | Systematic reviews of randomized trials or nested case-control studies, n=1 studies, or observational studies with dramatic effect | Randomized trials or (outstanding) observational studies with dramatic effect | Non-randomized controlled cohort / follow-up studies (post-market observation), assuming sufficient case numbers to exclude common harms. (For long-term harms, the follow-up period must be sufficient) | Case series or case-control studies, or historical controlled studies. | Mechanism-based reasoning |
| What are the rare harms/harm from the intervention? Treatment disadvantage | Systematic reviews of randomized trials or n=1 trials. | Randomized trials or outstanding observational studies with dramatic effect |  | Case series or case-control studies, or historical controlled trials | Mechanism-based reasoning |
| Is the (early detection) test worthwhile? (screening) | Systematic reviews of randomized trials | Randomized trials | Non-randomized controlled cohorts / follow-up studies | Case series or case-control studies, or historical controlled studies | Mechanism-based reasoning |

## **Table S3. Clinical classification of esophageal cancers, including carcinomas of the esophagogastric junction according to TNM classification.**

| T - Primary Tumor | |
| --- | --- |
| TX | Primary tumor cannot be assessed |
| T0 | No evidence of primary tumor |
| Tis | Carcinoma in situ |
| T1 | Tumor infiltrates lamina propria, muscularis mucosae or submucosa |
| T1a | Tumor infiltrating lamina propria, muscularis mucosae |
| T1b | Tumor infiltrating submucosa |
| T2 | Tumor infiltrates muscularis propria |
| T3 | Tumor infiltrates adventitia |
| T4 | Tumor infiltrates adjacent structures |
| T4a | Tumor infiltrates pleura, pericardium, azygos vein, diaphragm or peritoneum |
| T4b | Tumor infiltrates other adjacent structures such as aorta, vertebral body or trachea |

| N - Regional lymph nodes | |
| --- | --- |
| NX | Regional lymph nodes cannot be assessed |
| N0 | No regional lymph node metastases |
| N1 | Metastases in 1 - 2 lymph nodes |
| N2 | Metastases in 3 - 6 lymph nodes |
| N3 | Metastases in 7 or more regional lymph nodes |

| M - Distant metastases | |
| --- | --- |
| M0 | No distant metastases |
| M1 | Distant metastases |
| **pTNM: Pathological classification (The pT and pN categories correspond to the T and N categories.)** | |
| pM1 | Distant metastases confirmed microscopically |
| pN0 | Regional lymphadenectomy and histological examination usually of 7 or more lymph nodes. |
| Note: pM0 and pMX are not applicable categories. | |
| Source: [[145]](#LITREF_240042) | |

## **Table S4. Stage grouping of the TNM classification of esophageal cancers, including carcinomas of the esophagogastric junction - Squamous cell carcinoma****.**

|  | T-category | N-category | M-category |
| --- | --- | --- | --- |
| **Clinical stage - Squamous cell carcinoma** | | | |
| Stage 0 | Tis | N0 | M0 |
| Stage I | T1 | N0, N1 | M0 |
| Stage II | T2 | N0, N1 | M0 |
|  | T3 | N0 | M0 |
| Stage III | T1,T2 | N2 | M0 |
|  | T3 | N1, N2 | M0 |
| Stage IVA | T4a, T4b | Any N | M0 |
| Stage IVA | Any T | N3 | M0 |
| Stage IVB | Any T | Any N | M1 |
| **Pathological stage - squamous cell carcinoma** | | | |
| Stage 0 | pTis | pN0 | M0 |
| Stage IA | pT1a | pN0 | M0 |
| Stage IB | PT1b | pn0 | M0 |
|  | pT2 | pN0 | M0 |
| Stage IIA | pT2 | pN0 | M0 |
| Stage IIB | pT1 | pN1 | M0 |
|  | pT3 | pN0 | M0 |
| Stage IIA | pT2 | pN0 | M0 |
| Stage IIIB | pT1 | pN1 | M0 |
|  | pT3 | pN0 | M0 |
|  | pT4a | pN0, pN1 | M0 |
| Stage IVA | pT4a | pN2 | M0 |
|  | pT4b | Any pN | M0 |
|  | Any pT | pN3 | M0 |
| Stage IVB | Any pT | Any pN | M1 |
| Source: [[145]](#LITREF_240042) | | | |

## **Table S5. Stage grouping of the TNM classification of esophageal cancers, including carcinomas of the esophagogastric junction – Adenocarcinoma****.**

| Stage | T-Category | N-category | M-category |
| --- | --- | --- | --- |
| **Clinical stage- Adenocarcinoma** | | | |
| Stage 0 | Tis | N0 | M0 |
| Stage I | T1 | N0 | M0 |
| Stage IIA | T1 | N1 | M0 |
| Stage IIB | T2 | N0 | M0 |
| Stage III | T2 | N1 | M0 |
|  | T3,T4a | N0, N1 | M0 |
| Stage IVA | T41-T4a | N2 | M0 |
|  | T4b | N0, N1, N2 | M0 |
|  | Any T | N3 | M0 |
| Stage IVB | Any T | Any N | M1 |
| **Pathological stage - Adenocarcinomas** | | | |
| Stage 0 | pTis | pn0 | M0 |
| Stage IA | pT1a | pN0 | M0 |
| Stage IB | pT1b | pN0 | M0 |
| Stage IIA | pT2 | pN0 | M0 |
| Stage IIB | pT1a, pT1b | pN1 | M0 |
| Stage IIIA | pT1 | pN2 | M0 |
|  | pT2 | pN1, pN2 | M0 |
|  | pT3, pT4a | pN0 |  |
| Stage IIIB | pT2 | pN2 | M0 |
|  | pT3 | pN1, pN2 | M0 |
|  | pT4a | pN1 | M0 |
| Stage IVA | pT4a | pN2 | M0 |
|  | pT4b | Any pN | M0 |
|  | Any pT | pN3 | M0 |
| Stage IVB | Any pT | Any pN | M1 |
| Source: [[145]](#LITREF_240042) | | | |
